# Measures of Malaria Burden after Long-Lasting Insecticidal Net Distribution and Indoor Residual Spraying at Three Sites in Uganda: A Prospective Observational Study

**DOI:** 10.1371/journal.pmed.1002167

**Published:** 2016-11-08

**Authors:** Agaba Katureebe, Kate Zinszer, Emmanuel Arinaitwe, John Rek, Elijah Kakande, Katia Charland, Ruth Kigozi, Maxwell Kilama, Joaniter Nankabirwa, Adoke Yeka, Henry Mawejje, Arthur Mpimbaza, Henry Katamba, Martin J. Donnelly, Philip J. Rosenthal, Chris Drakeley, Steve W. Lindsay, Sarah G. Staedke, David L. Smith, Bryan Greenhouse, Moses R. Kamya, Grant Dorsey

**Affiliations:** 1 Infectious Diseases Research Collaboration, Kampala, Uganda; 2 HealthMap, Boston Children’s Hospital, Boston, Massachusetts, United States of America; 3 IMS Brogan Health, Montreal, Quebec, Canada; 4 Uganda Malaria Surveillance Project, Kampala, Uganda; 5 School of Medicine, Makerere University College of Health Sciences, Kampala, Uganda; 6 Child Health & Development Centre, Makerere University College of Health Sciences, Kampala, Uganda; 7 Uganda Ministry of Health, Kampala, Uganda; 8 Department of Vector Biology, Liverpool School of Tropical Medicine, Liverpool, United Kingdom; 9 Department of Medicine, San Francisco General Hospital, University of California, San Francisco, San Francisco, California, United States of America; 10 London School of Hygiene and Tropical Medicine, London, United Kingdom; 11 School of Biological and Biomedical Sciences, Durham University, Durham, United Kingdom; 12 Institute for Health Metrics and Evaluation, University of Washington, Seattle, Washington, United States of America; Liverpool School of Tropical Medicine, UNITED KINGDOM

## Abstract

**Background:**

Long-lasting insecticidal nets (LLINs) and indoor residual spraying of insecticide (IRS) are the primary vector control interventions used to prevent malaria in Africa. Although both interventions are effective in some settings, high-quality evidence is rarely available to evaluate their effectiveness following deployment by a national malaria control program. In Uganda, we measured changes in key malaria indicators following universal LLIN distribution in three sites, with the addition of IRS at one of these sites.

**Methods and Findings:**

Comprehensive malaria surveillance was conducted from October 1, 2011, to March 31, 2016, in three sub-counties with relatively low (Walukuba), moderate (Kihihi), and high transmission (Nagongera). Between 2013 and 2014, universal LLIN distribution campaigns were conducted in all sites, and in December 2014, IRS with the carbamate bendiocarb was initiated in Nagongera. High-quality surveillance evaluated malaria metrics and mosquito exposure before and after interventions through (a) enhanced health-facility-based surveillance to estimate malaria test positivity rate (TPR), expressed as the number testing positive for malaria/number tested for malaria (number of children tested for malaria: Walukuba = 42,833, Kihihi = 28,790, and Nagongera = 38,690); (b) cohort studies to estimate the incidence of malaria, expressed as the number of episodes per person-year [PPY] at risk (number of children observed: Walukuba = 340, Kihihi = 380, and Nagongera = 361); and (c) entomology surveys to estimate household-level human biting rate (HBR), expressed as the number of female *Anopheles* mosquitoes collected per house-night of collection (number of households observed: Walukuba = 117, Kihihi = 107, and Nagongera = 107). The LLIN distribution campaign substantially increased LLIN coverage levels at the three sites to between 65.0% and 95.5% of households with at least one LLIN. In Walukuba, over the 28-mo post-intervention period, universal LLIN distribution was associated with no change in the incidence of malaria (0.39 episodes PPY pre-intervention versus 0.20 post-intervention; adjusted rate ratio [aRR] = 1.02, 95% CI 0.36–2.91, *p* = 0.97) and non-significant reductions in the TPR (26.5% pre-intervention versus 26.2% post-intervention; aRR = 0.70, 95% CI 0.46–1.06, *p* = 0.09) and HBR (1.07 mosquitoes per house-night pre-intervention versus 0.71 post-intervention; aRR = 0.41, 95% CI 0.14–1.18, *p* = 0.10). In Kihihi, over the 21-mo post-intervention period, universal LLIN distribution was associated with a reduction in the incidence of malaria (1.77 pre-intervention versus 1.89 post-intervention; aRR = 0.65, 95% CI 0.43–0.98, *p* = 0.04) but no significant change in the TPR (49.3% pre-intervention versus 45.9% post-intervention; aRR = 0.83, 95% 0.58–1.18, *p* = 0.30) or HBR (4.06 pre-intervention versus 2.44 post-intervention; aRR = 0.71, 95% CI 0.30–1.64, *p* = 0.40). In Nagongera, over the 12-mo post-intervention period, universal LLIN distribution was associated with a reduction in the TPR (45.3% pre-intervention versus 36.5% post-intervention; aRR = 0.82, 95% CI 0.76–0.88, *p <* 0.001) but no significant change in the incidence of malaria (2.82 pre-intervention versus 3.28 post-intervention; aRR = 1.10, 95% 0.76–1.59, *p* = 0.60) or HBR (41.04 pre-intervention versus 20.15 post-intervention; aRR = 0.87, 95% CI 0.31–2.47, *p* = 0.80). The addition of three rounds of IRS at ~6-mo intervals in Nagongera was followed by clear decreases in all outcomes: incidence of malaria (3.25 pre-intervention versus 0.63 post-intervention; aRR = 0.13, 95% CI 0.07–0.27, *p <* 0.001), TPR (37.8% pre-intervention versus 15.0% post-intervention; aRR = 0.54, 95% CI 0.49–0.60, *p <* 0.001), and HBR (18.71 pre-intervention versus 3.23 post-intervention; aRR = 0.29, 95% CI 0.17–0.50, *p <* 0.001). High levels of pyrethroid resistance were documented at all three study sites. Limitations of the study included the observational study design, the lack of contemporaneous control groups, and that the interventions were implemented under programmatic conditions.

**Conclusions:**

Universal distribution of LLINs at three sites with varying transmission intensity was associated with modest declines in the burden of malaria for some indicators, but the addition of IRS at the highest transmission site was associated with a marked decline in the burden of malaria for all indicators. In highly endemic areas of Africa with widespread pyrethroid resistance, IRS using alternative insecticide formulations may be needed to achieve substantial gains in malaria control.

## Abstract 2

In this prospective observational study, Grant Dorsey and colleagues measure changes in malaria burden after long-lasting insecticidal net distribution and indoor residual spraying at three sites of in Uganda.

## Author Summary

**Why Was This Study Done?:**

- Long-lasting insecticidal nets (LLINs), which prevent mosquitoes from biting people while they sleep, and indoor residual spraying of insecticides (IRS) in houses, which prevents mosquitoes from resting in houses, are the main tools used to prevent malaria in Africa.
- Although LLINs and IRS have been shown to be effective, changes in the behavior of mosquitoes and people as well as the emergence of mosquitoes resistant to insecticides could compromise the benefits of these interventions.
- Recently the government of Uganda distributed free LLINs throughout the country and began IRS in selected areas. In this “real world” setting, it is important to monitor for changes in the burden of malaria following the scale-up of LLIN distribution and IRS.

**What Did the Researchers Do and Find?:**

- The researchers conducted comprehensive malaria surveillance between October 1, 2011, and March 31, 2016, at three sites in Uganda that differed in the intensity of malaria transmission.
- Between 2013 and 2014, LLINs were distributed to the entire population at all three sites, and in December 2014, IRS with an insecticide different from that used in the LLINs was started in the site with the highest level of malaria transmission.
- The researchers found that following LLIN distribution, there were only modest declines in some measures of malaria burden at the three sites, and no changes in other measures.
- In contrast, following the addition of IRS at the highest transmission site, all measures of malaria burden declined dramatically.
- The research also documented a high level of resistance to the type of insecticide used in LLINS at all three sites.

**What Do These Findings Mean?:**

- These findings suggest that in countries like Uganda, which has a heavy burden of malaria, LLINs alone may not be adequate to substantially drive down the burden of malaria, and the addition of IRS using a different insecticide may be needed to have a major impact.
- However, it should be noted that IRS is more expensive and harder to implement than distributing LLINs, and generally needs to be repeated every 6–12 months to have a sustained effect.

## Introduction

Over the last fifteen years, funding for malaria control activities has increased dramatically across Africa, leading to the scale-up of proven interventions including distribution of long-lasting insecticidal nets (LLINs), indoor residual spraying of insecticide (IRS), and treatment of malaria cases with artemisinin-based combination therapy (ACT) [1]. Substantial declines in measures of malaria burden have been attributed to the expansion of these interventions at various scales, from the sub-national level to the entire continent [2–4]. Despite these advances, the burden of malaria remains high, with an estimated 215 million cases and 438,000 deaths worldwide in 2015, of which 88% of cases and 90% of deaths were in Africa [1].

LLINs have been shown to reduce malaria morbidity and mortality across a range of epidemiological settings, and the World Health Organization (WHO) recommends universal coverage of populations at risk [5,6]. IRS has also been shown to be highly effective, but it is more resource-intensive and expensive to implement than distribution of LLINs [7,8]. Historically, IRS played a key role in the global malaria elimination campaign in the 1950s and 1960s, but was not widely used in sub-Saharan Africa primarily due to limited resources [9]. More recently, the use of IRS in sub-Saharan Africa has been expanded from epidemic-prone areas with seasonal transmission to areas with more intense perennial transmission [10]. However, despite the widespread use of LLINs and IRS, and the importance of quantifying the impact of malaria control interventions in operational settings, high-quality contemporary data are limited. This is largely due to a paucity of rigorous longitudinal malaria surveillance studies that can capture the impact of interventions over time. Indeed, most estimates of the impact of population-level malaria control interventions rely on health facility records, which are often incomplete and/or inaccurate, or repeated cross-sectional surveys that measure parasite prevalence, which provides only an indirect estimate of morbidity.

We report findings from a comprehensive malaria surveillance program conducted in three areas of Uganda with varied transmission intensities from October 2011 to March 2016, a period of major expansion in population-level malaria control interventions. Uganda has reported some of the highest levels of transmission intensity and ranks fourth globally in the estimated number of annual cases of malaria [1,11]. Given that malaria is endemic in over 95% of the country and the burden remains high in many areas, efforts have focused on control and not elimination [12]. From 2012 to 2014, Uganda implemented a national universal LLIN distribution campaign, with 21 million LLINs distributed to a population of approximately 35 million people [13]. In December 2014, IRS was implemented for the first time in one of our three study areas. Evaluations included enhanced health-facility-based surveillance to estimate malaria test positivity rate (TPR), cohort studies to estimate the incidence of malaria, entomology surveys to estimate transmission intensity, insecticide susceptibility testing, and repeated cross-sectional community surveys to estimate the coverage level of key malaria control interventions.

## Methods

### Ethics Statement

Ethical approval was obtained from the Makerere University School of Medicine Research and Ethics Committee, the Uganda National Council for Science and Technology, the London School of Hygiene & Tropical Medicine Ethics Committee, the Durham University School of Biological and Biomedical Sciences Ethics Committee, and the University of California, San Francisco, Committee on Human Research.

### Study Sites

**Fig 1:**
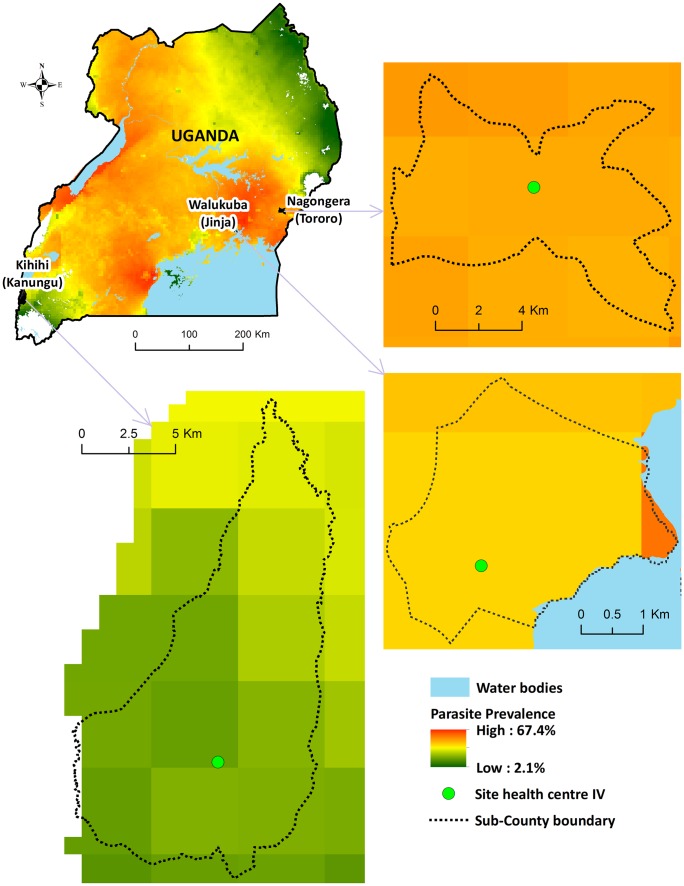
Map of Uganda showing study sites. Parasite prevalence in children 2–10 y of age from cross-sectional surveys conducted in 2012: Walukuba = 16%; Kihihi = 18%; Nagongera = 60% [14].

Comprehensive surveillance studies were conducted in three sub-counties in Uganda: Walukuba, Jinja District; Kihihi, Kanungu District, and Nagongera, Tororo District (Fig 1). These areas were chosen to represent varied malaria transmission settings. Walukuba is a relatively low transmission, peri-urban area near Lake Victoria in the south-central part of the country. Kihihi is a rural area with moderate transmission intensity bordering a national park in the southwestern part of the country. Nagongera is a rural area with high transmission intensity in the southeastern part of the country near to the border with Kenya (Fig 1). Transmission in all of these areas is perennial, with two annual peaks following the rainy seasons.

### Health-Facility-Based Surveillance

Enhanced malaria surveillance was conducted at one government-run level IV health center at each study site, as described previously [15]. Briefly, individual-level data on all patients presenting to the outpatient department of the facilities were collected electronically using a standardized data collection form. Data collected included patient age, whether symptomatic malaria was suspected (as determined by the evaluating clinician), whether laboratory testing for malaria was done, the type of laboratory testing done (either light microscopy or rapid diagnostic test), and the results of the laboratory test. Study staff visited the health facilities periodically to provide training and feedback including quality control of diagnostic testing. Enhanced surveillance was begun in 2006, but this report only includes data from October 1, 2011, to March 31, 2016, when data became available from the other malaria surveillance studies described below.

### Cohort Studies

Cohort studies were performed in children from households randomly selected from enumeration surveys conducted in each of the three sub-counties, as previously described [16]. Briefly, all eligible children aged 0.5–10 y were enrolled from 100 households from each study site between August and September 2011. The cohorts were dynamic, such that all newly eligible children were enrolled during follow-up and study participants who reached 11 y of age were excluded from further follow-up. At enrolment, written informed consent was provided by parents/guardians and study participants were given an LLIN and underwent a standardized evaluation. Cohort study participants received all medical care free of charge at designated study clinics open every day. Parents/guardians were encouraged to bring their children to the clinic any time they were ill and were reimbursed for transport costs. Children who presented with a documented fever (tympanic temperature ≥ 38.0°C) or history of fever in the previous 24 h had blood obtained by finger prick for a thick blood smear. If the smear was positive for malaria parasites, the patient was diagnosed with malaria. Episodes of uncomplicated malaria were treated with artemether-lumefantrine, and episodes of complicated malaria or recurrent malaria occurring within 14 d of prior therapy were treated with quinine. Study participants were withdrawn from the study for (a) permanent movement out of the sub-county, (b) inability to be located for >4 mo, (c) withdrawal of informed consent, (d) inability to comply with the study schedule and procedures, or (e) reaching 11 y of age. Children from 31 randomly selected additional households were enrolled between August and October 2013 to replace households in which all study participants had been withdrawn and were followed using the same procedures described above. A full description of the selection of study households and participants is provided in S1 Fig.

### Entomology Surveys

For each household participating in the cohort studies, entomological surveys were performed, as described previously [16,17]. Briefly, mosquitoes were collected once a month from each cohort study household using miniature CDC light traps (Model 512; John W. Hock Company) with the light positioned 1 m above the floor at the foot end of the bed where a cohort study participant slept. Traps were set at 19.00 h and collected at 07.00 h the following morning by field workers. Female *Anopheles* mosquitoes were identified taxonomically to species level based on morphological criteria according to established taxonomic keys [18]. Members of the *A*. *gambiae* complex were identified by PCR [19] for 30 mosquitoes randomly selected at each site each month. Sporozoites were identified in individual mosquitoes stored with desiccant using an ELISA technique [20]. All female *Anopheles* mosquitoes captured in Walukuba and Kihihi were tested for sporozoites. Due to the large numbers of female *Anopheles* mosquitoes captured in Nagongera, only up to 50 randomly selected mosquitoes per household per night of collection were tested for sporozoites.

### Cross-sectional Community Surveys

Annual cross-sectional surveys were conducted in each of the study sites in 2012, 2013, and 2015, as previously described [14]. Briefly, for each survey, households were randomly selected from our enumeration list and sequentially screened until 200 households were enrolled. The purpose of the study was discussed with the head of the household or their designate, and consent to participate in the survey was sought. Households with no adult respondent during the initial contact were revisited up to three times before excluding them from the sample selection. After obtaining written informed consent, a household questionnaire was administered to the head of the household or their designate. The questionnaire was used to capture information on the demographics of all household members and the use of malaria prevention methods. The surveys were conducted over the same 2-mo period each year in each of three sub-counties: Walukuba, Jinja District (March–April); Kihihi, Kanungu District (May–June); and Nagongera, Tororo District (January–February).

### Population-Level Malaria Control Interventions

Prior to 2012, a number of sub-national LLIN distribution campaigns were carried out in Uganda. From September 2012 through August 2014, the government of Uganda carried out a countrywide mass distribution of free LLINs. An estimated 21 million LLINs were distributed, with the goal of achieving universal coverage with at least one LLIN for every two people. For our study sites, LLINs were distributed during November 2013 in Jinja and Tororo Districts, which included Walukuba and Nagongera, and during June 2014 in Kanungu District, which included Kihihi. Data on the population at risk and the number of LLINs distributed at our study sites were obtained from the Ugandan National Malaria Control Program (NMCP).

In 2006, Uganda initiated an IRS program, initially focusing on epidemic-prone areas in the southwestern part of the country. In 2009, the IRS program was moved to ten northern districts with high transmission intensity. In 2014–2015, the IRS program was moved to 14 districts in the Lango, Bukedi, and Teso sub-regions located in the central and eastern part of the country [13]. With respect to our study sites, Walukuba sub-county has not received IRS, and Kihihi sub-county received a single round of IRS using the pyrethroid lambda-cyhalothrin in February–March 2007. In Tororo District, including Nagongera sub-county, the first round of IRS using the carbamate bendiocarb was delivered in December 2014–February 2015, a second round in June–July 2015, and a third round in November–December 2015, with plans to continue IRS every 6 mo for at least 3 y. Data on the number of households targeted and the number that received IRS in Nagongera were obtained from the Ugandan NMCP.

### Measurement of Other Covariates of Interest

Estimates of monthly rainfall were obtained from the NASA Tropical Rainfall Measuring Mission [21]. Insecticide susceptibility testing was conducted at the three study sites from January to June 2014, as described previously [22]. Briefly, mosquitoes were collected as larvae using the dipping method from a variety of breeding sites. Larvae were transferred to an insectary and reared to adulthood. Emerging adults were fed on a 10% sugar solution and identified as belonging to the *A*. *gambiae* species complex using morphological keys. Non-blood-fed female mosquitoes (3–5 d old) were exposed to insecticide-treated papers impregnated with WHO diagnostic concentrations (4% DDT, 0.05% deltamethrin, 0.75% permethrin, 0.1% bendiocarb, 1% fenitrothion, and 5% malathion). Batches of 20–25 mosquitoes were exposed to each insecticide for 1 h, and mortality scored 24 h post-exposure in accordance with standard WHO insecticide susceptibility testing procedures.

### Statistical Analysis

One of the primary objectives of establishing our comprehensive malaria surveillance study was to estimate the changes in measures of transmission intensity, infection, and disease using surveillance data at multiple sites in Uganda following the implementation of malaria control measures (as described in S1–S4 Texts). It was originally anticipated that community-level changes in the coverage of key malaria control interventions would be gradual. To make causal inferences on the effect of these interventions, a counterfactual framework was originally planned to estimate what the outcome variable of interest would be if the malaria control intervention were set to a specific level. However, following the establishment of our comprehensive malaria surveillance study, the Uganda NMCP rapidly implemented two major interventions (universal LLIN distribution and IRS) covering our study sites. Given this opportunity, we adjusted our analytical plan and performed the “before and after” comparisons described below.

All data were collected using standardized data collection forms and entered using Microsoft Access. Analyses were performed using Stata version 14 (StataCorp) and R version 3.2.1 (https://www.r-project.org/). Data for all analyses described below covered the period from October 1, 2011, through March 31, 2016. For health-facility-based surveillance, the primary metric was the TPR, defined as the proportion of patients tested for malaria who tested positive by microscopy or rapid diagnostic test. Data from health-facility-based surveillance used in this report only included children 0.5 to 10 y of age to mirror the age range included in the cohort studies. For the cohort studies, the primary metric was malaria incidence, defined as the number of new episodes of malaria per person-year [PPY] of observation. New episodes of malaria were defined as any episode of laboratory-confirmed malaria not preceded by another episode of malaria in the prior 14 d. For the entomology surveys, the primary metric was the daily human biting rate (HBR), defined as the total number of female *Anopheles* mosquitoes captured per house-night of collection from the same households participating in the cohort studies. Monthly time series were created for each of the three surveillance methods. An LLIN variable was created with time periods corresponding to the dates before and after the universal distribution campaigns were completed at each study site (cutoff at December 1, 2013, for Walukuba and Nagongera and July 1, 2014, for Kihihi). For Nagongera, an IRS variable was created with time periods corresponding the dates after the LLIN distribution campaign through 2 mo following the initiation of the first round of IRS (December 1, 2013–January 31, 2015) and to 2 mo after the initiation of each subsequent round of IRS (cutoffs at February 1, 2015, August 1, 2015, and January 1, 2016). Autoregressive integrated moving average (ARIMA) models were used and, specifically, the ARIMAX form of ARIMA models was used for this study, which is a multivariate transfer function model and includes current and/or past values of independent variables as predictors [23]. ARIMAX models were developed to estimate the adjusted rate ratios (aRRs) when comparing metrics before and after the interventions. Potential confounders included in the models were age, gender, and rainfall at a 1-mo lag. Seasonal terms were examined for each model, and candidate models were selected through the inspection of residual autocorrelation diagnostics via the autocorrelation function, the partial autocorrelation function, and the Ljung-Box test. The Akaike information criteria of all candidate models were compared for selection of the final model. Additional details of the ARIMA models used in the time-series analyses are provided in S1 Table. A *p*-value < 0.05 was considered statistically significant.

This study provided the opportunity to evaluate relationships between temporal changes in malaria incidence measured in the cohort studies and TPR measured by health-facility-based surveillance. While a direct measure of malaria incidence is considered the gold standard for estimating malaria morbidity, the TPR offers a surrogate measure [24]. Assuming that patients undergoing laboratory testing for malaria at a health facility are representative of the catchment population, the relationship between the TPR and the true incidence of malaria (Im) and non-malarial fevers (Inm) for any time interval can be defined as follows:

$$\frac{\text{TPR}}{1-\text{TPR}}=\frac{\text{Im}}{\text{Inm}}$$

From the cohort studies, the observed relative monthly change in the incidence of malaria (observed rΔIm) was defined as Im_*i+*1_/Im_*i*_, where *i* represents the month of observation. For the health-facility-based surveillance, rΔIm can be predicted from the TPR using the following formula:

$$\text{predicted r}\Delta \text{Im}=\frac{{\text{Inm}}_{i+1}}{{\text{Inm}}_{i}}\times \frac{{\text{TPR}}_{i+1}\left(1-{\text{TPR}}_{i}\right)}{{\text{TPR}}_{i}\left(1-{\text{TPR}}_{i+1}\right)}$$

For health-facility-based surveillance, the incidence of non-malarial fevers in the catchment populations was unknown. Therefore, estimates of the incidence of non-malarial fevers from the cohort studies (defined as the number of new episodes of fever with a negative blood smear PPY of observation) were used when calculating the predicted rΔIm. Relationships between relative monthly changes in the incidence of malaria observed from the cohort studies and those predicted from health-facility-based surveillance (log transformed) were investigated using the Pearson correlation coefficient.

## Results

**Fig 2:**
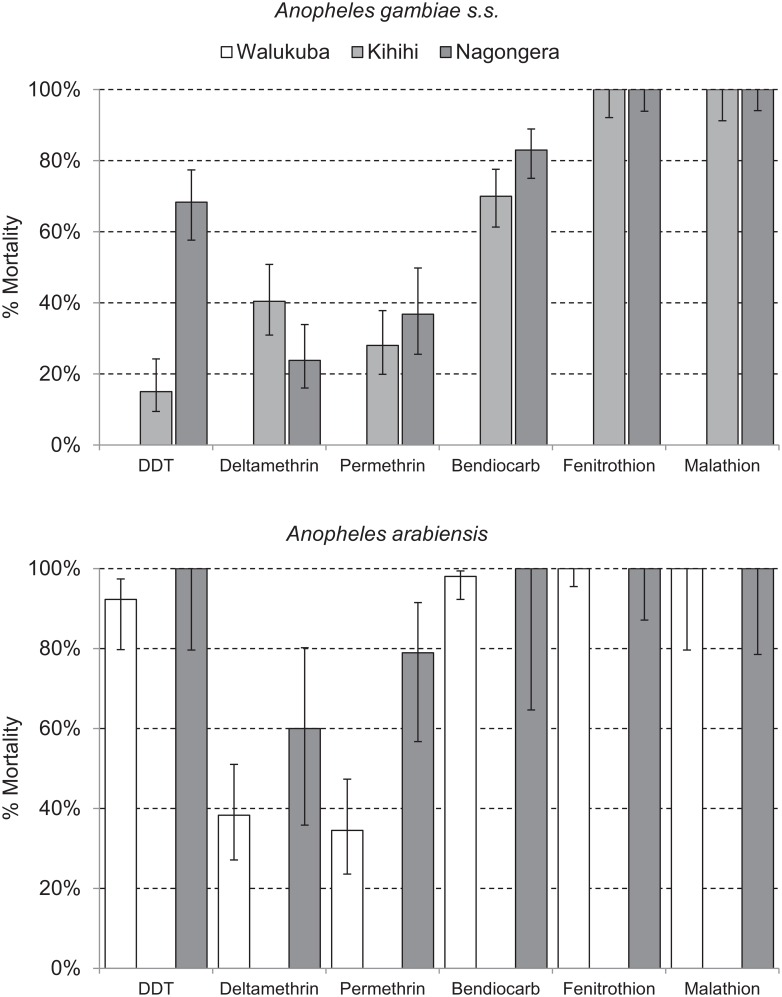
Insecticide susceptibility testing: 24-h mortality for *A*. *gambiae* s.s. (Kihihi and Nagongera only) and *A*. *arabiensis* (Walukuba and Nagongera only) exposed for 1 h to insecticide-treated papers impregnated with WHO diagnostic concentrations of insecticides. By WHO convention, mortality of 98%–100% indicates susceptibility, <98% is suggestive of resistance, and <90% is strongly suggestive of resistance. Error bars give 95% standard errors.

Health-facility-based surveillance involved a total of 110,313 outpatient visits among children 0.5 to 10 y of age from all three sites combined over the 4.5-y observation period (Table 1). The proportion of visits for which malaria was suspected ranged from 54.9% to 82.4% across the three sites. Over 98% of patients with suspected malaria underwent laboratory testing at all three sites, and the TPR ranged from 26.4% in Walukuba to 48.4% in Kihihi. For the cohort studies, a total of 1,081 children were observed over 3,258 person-years. A total of 5,213 episodes of malaria were diagnosed, with an incidence ranging from 0.29 episodes PPY in Walukuba to 2.41 episodes PPY in Nagongera. Only 12 episodes of malaria (0.2% of total) met criteria for severe malaria (5 severe anemia, 4 multiple convulsions, 2 cerebral malaria, and 1 respiratory distress). There were no deaths due to malaria. Two children with negative blood smears died of diarrheal illnesses. Monthly entomology surveys were conducted in 331 households involving 15,206 nights of collection. A total of 155,613 female *Anopheles* mosquitoes were collected, demonstrating daily HBRs ranging from 0.88 in Walukuba to 26.12 in Nagongera and sporozoite rates ranging from 0.84% in Walukuba to 1.84% in Nagongera. Estimates of the annual entomological inoculation rate were 2.71, 20.90, and 175.54 infectious bites PPY in Walukuba, Kihihi, and Nagongera, respectively (Table 1). The primary vector species in Walukuba was *A*. *arabiensis*, followed by *A*. *gambiae* s.s. and *A*. *funestus*. In Kihihi almost all mosquitoes were *A*. *gambiae* s.s., and in Nagongera the primary vector was *A*. *gambiae* s.s., followed by *A*. *arabiensis* and *A*. *funestus* (Table 1). Insecticide susceptibility testing was performed using WHO bioassays for available vector species from the study sites in 2014. Testing of *A*. *gambiae* s.s. in Kihihi and Nagongera revealed moderate to high resistance to DDT and pyrethroids (deltamethrin and permethrin), lower resistance to bendiocarb, and full susceptibility to organophosphates (fenitrothion and malathion). Testing of *A*. *arabiensis* in Walukuba and Nagongera revealed low resistance to DDT (Walukuba only), high resistance to pyrethroids, and full susceptibility to bendiocarb and organophosphates (Fig 2).

**Table 1:** Summary statistics from longitudinal surveillance studies.

Data Source	Metric	Study Site		
		Walukuba	Kihihi	Nagongera
**Health-facility-based surveillance**	**Visits for children 0.5–10 y of age**	42,833	28,790	38,690
	**Malaria suspected (percent of total)**	23,529 (54.9%)	23,733 (82.4%)	31,847 (82.3%)
	**Laboratory testing done (percent of suspected)**	23,245 (98.8%)	23,656 (99.6%)	31,410 (98.6%)
	**Tested positive for malaria (percent of tested)**	6,141 (26.4%)	11,442 (48.4%)	12,209 (38.9%)
**Cohort studies**	**Number of children observed**	340	380	361
	**Person-years of observation**	903	1,221	1,134
	**Number of episodes of malaria**	261	2,216	2,736
	**Incidence of malaria (new episodes per person-year)**	0.29	1.81	2.41
**Entomology surveys**	**Number of households observed**	117	107	107
	**Number of nights of collection**	4,951	5,141	5,114
	**Total female *Anopheles* mosquitoes collected**	4,377	17,657	133,579
	***Anopheles* species**			
	*A*. *gambiae* s.s.	34.1%	98.1%	67.5%
	*A*. *arabiensis*	54.3%	0.6%	21.6%
	*A*. *funestus*	5.3%	0.7%	10.1%
	Other	6.4%	0.5%	0.8%
	**Daily human biting rate (mosquitoes per house-night)**	0.88	3.43	26.12
	**Sporozoite rate** ^1^	0.84%	1.67%	1.84%
	**Annual entomological inoculation rate** ^2^	2.71	20.90	175.54

Data are given as number, number (percent), or percent, unless otherwise indicated.

^1^Number of mosquitoes testing positive for sporozoites/the number of mosquitoes tested.

^2^Daily human biting rate × sporozoite rate × 365 d/y.

Coverage levels of key malaria control interventions were obtained from repeated cross-sectional community surveys, routine assessments of cohort participants, and data from the Ugandan NMCP. In 2012, the proportion of households with at least one LLIN ranged from 51.0% in Kihihi to 78.5% in Nagongera, with slightly lower estimates in 2013. Considering the proportion of households with at least one LLIN per two persons, coverage levels were relatively low at all sites in 2012 and 2013 (range 17.0%–35.5%), consistent with a lack of sufficient numbers of LLINs even among households that owned at least one. According to data from the NMCP, the number of LLINs distributed in our study sites ranged from 0.63 per person in Kihihi to 0.80 per person in Walukuba. In cross-sectional surveys conducted 10–17 mo following the universal LLIN distribution campaigns, the proportion of households with at least one LLIN increased from the prior survey modestly in Walukuba (from 49.0% to 65.0%) and more substantially in Kihihi (from 37.5% to 86.5%) and Nagongera (from 71.0% to 95.5%). Considering the proportion of households with at least one LLIN per two persons, increases from the prior survey were even greater at all sites (Walukuba 31.0% to 52.0%, Kihihi 19.0% to 73.0%, and Nagongera 22.5% to 62.0%; Table 2). All cohort study participants and their family members were provided LLINs at enrolment. Over 99% of children were reported to have slept under an LLIN the prior evening at the time of routine visits. Government-supported IRS campaigns were not conducted in Walukuba and Kihihi, and cross-sectional survey data revealed only a few households that reported recent IRS use. In Nagongera, an IRS campaign was begun in December 2014, with plans to spray with the carbamate bendiocarb every 6 mo. According to data from the NMCP, over 95% of houses were sprayed during each of the three rounds. Data from our cross-sectional survey conducted in January–February 2015 estimated that 78% of houses received IRS, but this is likely an underestimate of the proportion of houses that were ultimately sprayed, as the first round of IRS was ongoing when this survey was conducted (Table 2). Among the households participating in the cohort study, over 94% received IRS during each of the three rounds conducted in Nagongera. ACT coverage among children reportedly treated for malaria from cross-sectional surveys was 75% or higher across all three surveys at all three sites, with the exception of Walukuba in 2013 (Table 2). Children in the cohort studies received treatment with artemether-lumefantrine for all episodes of laboratory-confirmed uncomplicated malaria.

**Table 2:** Coverage level of malaria control interventions.

Metric	Time Period or Measure	Study Site		
		Walukuba	Kihihi	Nagongera
**LLIN coverage**				
Proportion of households with at least one LLIN^1^	2012^2^	57.5%	51.0%	78.5%
	2013^2^	49.0%	37.5%	71.0%
	2015^2^	65.0%	86.5%	95.5%
Proportion of households with at least one LLIN per two persons^1^	2012^2^	28.5%	17.0%	35.5%
	2013^2^	31.0%	19.0%	22.5%
	2015^2^	52.0%	73.0%	62.0%
Universal LLIN distribution campaign	Date	November 2013	June 2014	November 2013
	Total population	29,020	54,443	38,867
	Number of LLINs distributed	23,456	34,420	27,120
**IRS coverage**				
Proportion of households reporting IRS in the prior 12 mo^1^	2012^2^	2.5%	0%	0%
	2013^2^	1.5%	0.5%	0%
	2015^2^	0%	0%	78.0%
Dates of community-wide IRS campaign (proportion of houses sprayed)	1st round	N/A	N/A	Dec 14–Feb 15 (96.9%)
	2nd round	N/A	N/A	Jun 15–Jul 15 (95.6%)
	3rd round	N/A	N/A	Nov 15–Dec 15 (96.8%)
**ACT coverage**				
Proportion of malaria cases in the prior 2 wk that were given ACT^1^	2012^2^	75.0%	82.1%	77.8%
	2013^2^	52.6%	75.0%	87.2%
	2015^2^	85.7%	91.7%	94.6%

^1^Data from cross-sectional surveys done in 200 households per study site per year.

^2^Surveys done each year January–February in Nagongera, March–April in Walukuba, and May–June in Kihihi.

ACT, artemisinin-based combination therapy; IRS, indoor residual spraying of insecticide; LLIN, long-lasting insecticidal net; N/A, not applicable.

**Fig 3:**
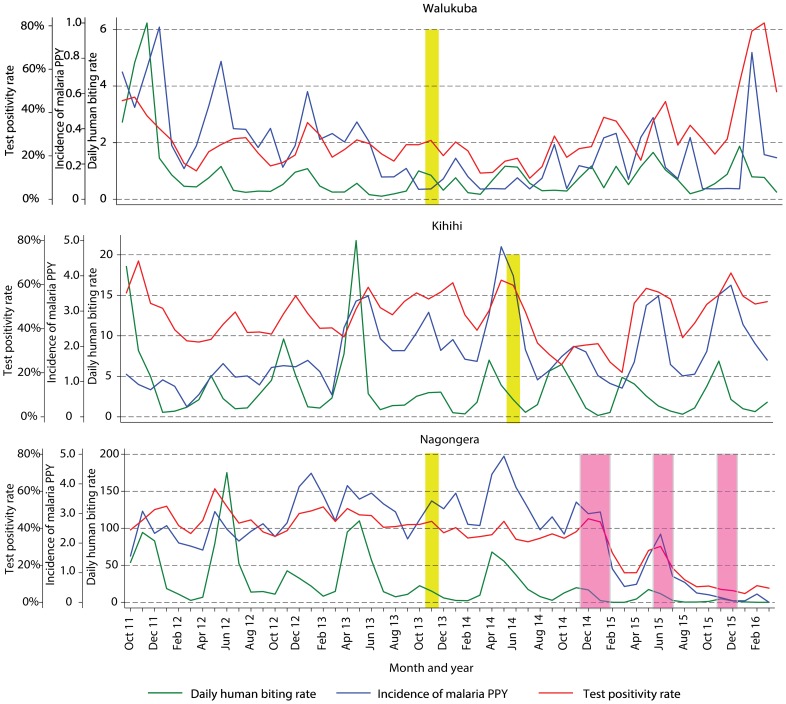
Temporal trends in monthly estimates of malaria test positivity rate from health-facility-based surveillance, incidence of malaria from cohort studies, and daily human biting rate from entomology surveys. Yellow vertical bars indicate when universal distribution of long-lasting insecticidal nets occurred. Pink vertical bars indicate when each round of indoor residual spraying with bendiocarb was implemented. Daily human biting rate is the number of female *Anopheles* mosquitoes captured per house-night of collection. PPY, per person-year.

Temporal trends in monthly crude estimates of malaria metrics from the different study sites are presented in Fig 3, and changes following universal LLIN distribution and IRS using time-series analyses to adjust for secular trends are presented in Table 3. In Walukuba, the lowest transmission site, malaria metrics were declining over the 26-mo period prior to universal distribution of LLINs. Over the 28-mo post-intervention period, universal LLIN distribution was associated with reductions in the TPR (aRR = 0.70, 95% CI 0.46–1.06, *p* = 0.09) and HBR (aRR = 0.41, 95% CI 0.14–1.18, *p* = 0.10) that did not reach statistical significance and no change in the incidence of malaria (aRR = 1.02, 95% CI 0.36–2.91, *p* = 0.97). In Kihihi, over the 21-mo post-intervention period, universal LLIN distribution was associated with a reduction in the incidence of malaria (aRR = 0.65, 95% CI 0.43–0.98, *p* = 0.04) but no significant change in the TPR (aRR = 0.83, 95% 0.58–1.18, *p* = 0.30) or HBR (aRR = 0.71, 95% CI 0.30–1.64, *p* = 0.40). In Nagongera, the highest transmission site, over the 12-mo post-intervention period prior to the implementation of IRS, universal LLIN distribution was associated with a reduction in the TPR (aRR = 0.82, 95% CI 0.76–0.88, *p <* 0.001) but no significant change in the incidence of malaria (aRR = 1.10, 95% CI 0.76–1.59, *p* = 0.60) or HBR (aRR = 0.87, 95% CI 0.31–2.47, *p* = 0.80). In contrast, compared to the 14-mo period following universal LLIN distribution, the added implementation of three rounds of IRS over a 14-mo period in Nagongera was associated with marked reductions in the TPR (aRR = 0.54, 95% CI 0.49–0.60, *p <* 0.001), incidence of malaria (aRR = 0.13, 95% CI 0.07–0.27, *p <* 0.001), and HBR (aRR = 0.29, 95% CI 0.17–0.50, *p <* 0.001).

**Table 3:** Malaria metrics at three study sites after universal long-lasting insecticidal net distribution and indoor residual spraying.

Study Site	Malaria Metric	Universal LLIN Distribution						Addition of IRS					
		Pre-intervention Period		Post-intervention Period		Adjusted Rate Ratio^1^ (95% CI)	*p-*Value	Pre-intervention Period		Post-intervention Period		Adjusted Rate Ratio^1^ (95% CI)	*p-*Value
		Period of Observation	Absolute Rate	Period of Observation	Absolute Rate			Period of Observation	Absolute Rate	Period of Observation	Absolute Rate		
**Walukuba**	Test positivity rate^2^	Oct 11–Nov 13 (26 mo)	26.5%	Dec 13–Mar 16 (28 mo)	26.2%	0.70 (0.46–1.06)	0.09	N/A					
	Incidence of malaria^3^		0.39		0.20	1.02 (0.36–2.91)	0.97						
	Human biting rate^4^		1.07		0.71	0.41 (0.14–1.18)	0.10						
**Kihihi**	Test positivity rate^2^	Oct 11–Jun 14 (33 mo)	49.3%	Jul 14–Mar 16 (21 mo)	45.9%	0.83 (0.58–1.18)	0.30	N/A					
	Incidence of malaria^3^		1.77		1.89	0.65 (0.43–0.98)	0.04						
	Human biting rate^4^		4.06		2.44	0.71 (0.30–1.64)	0.40						
**Nagongera**	Test positivity rate^2^	Oct 11–Nov 13 (26 mo)	45.3%	Dec 13–Nov 14 (12 mo)	36.5%	0.82 (0.76–0.88)	<0.001	Dec 13–Jan 15 (14 mo)	37.8%	Feb 15–Mar 16 (14 mo)	15.0%	0.54 (0.49–0.60)	<0.001
	Incidence of malaria^3^		2.82		3.28	1.10 (0.76–1.59)	0.60		3.25		0.63	0.13 (0.07–0.27)	<0.001
	Human biting rate^4^		41.40		20.15	0.87 (0.31–2.47)	0.80		18.71		3.23	0.29 (0.17–0.50)	<0.001

^1^Rate ratio adjusted for secular trends and rainfall with 1-mo lag.

^2^Number testing positive for malaria/total number tested for malaria.

^3^Episodes of malaria per person-year at risk.

^4^Number of female *Anopheles* mosquitoes collected per house-night of collection.

IRS, indoor residual spraying of insecticide; LLIN, long-lasting insecticidal net; N/A, not applicable.

This study provided the opportunity to evaluate relationships between temporal changes in two complementary measures of malaria morbidity: malaria incidence measured in the cohort studies and TPR measured by health-facility-based surveillance. Linear correlations between relative monthly changes in the incidence of malaria observed in the cohort studies and predicted from the health-facility-based surveillance TPRs are presented in S2 Fig. At all three sites, there were significant positive correlations between these two measures, with correlation coefficients ranging from 0.39 in Kihihi to 0.66 in Nagongera. The slopes for the lines of best fit were <1 for all three sites, indicating that predictions from the TPRs tended to be lower than observations from the cohort studies. The cumulative monthly relative changes in the incidence of malaria observed in the cohort studies and predicted from the health-facility-based surveillance TPRs are presented in S3 Fig. These two measures tracked well over time and in relation to the implementation of LLIN distribution and IRS, although malaria incidence relative to baseline predicted from the TPRs tended to be lower than what was observed from the cohort studies.

## Discussion

We utilized a comprehensive malaria surveillance system in three sites in Uganda with varied malaria epidemiology to measure changes in malaria metrics and mosquito exposure before and after malaria control interventions were delivered under operational conditions at the population level. The primary intervention was a national universal LLIN distribution campaign, with the goal of providing one LLIN for every two persons. The distribution campaign substantially increased LLIN coverage levels, but did not reach the universal coverage target. LLIN distribution was associated with modest reductions in malaria TPRs at all three sites, but the reduction reached statistical significance only in the highest transmission intensity site (Nagongera). There was a reduction in the incidence of malaria only in the medium transmission site (Kihihi) and reductions in the HBR that did not reach statistical significance at any of the sites. In contrast, in the highest transmission site (Nagongera), delivery of three rounds of IRS with the carbamate bendiocarb was associated with marked declines in all three malaria metrics. Notably, we documented high-level pyrethroid resistance among *A*. *gambiae* s.s. and *A*. *arabiensis* vectors across our study sites, which may have contributed to the limited changes seen following the distribution of LLINs. Our results suggest that IRS with non-pyrethroid insecticides is currently the most effective intervention available for reducing the burden of malaria in Uganda in areas where maximum bednet coverage is not obtained despite attempts at universal distribution.

Insecticide-treated nets (ITNs), including LLINs, are the most widely used intervention for the control of malaria in Africa, and universal coverage is recommended by WHO. The benefits of ITNs have been well established in several randomized controlled trials, with use of ITNs associated with a reduction in the incidence of malaria of 50% and a reduction in child mortality of 20% [5]. Analyses of observational data from cross-sectional surveys have suggested that the protective efficacy of ITNs under operational settings is similar to that observed in controlled trials [6]. Recently, using a large database of malaria field surveys, it was estimated that the incidence of malaria decreased by 40% across sub-Saharan Africa between 2000 and 2015, and that ITNs were responsible for 68% of cases averted [2]. Although these estimates are encouraging, they are based on measurement of parasite prevalence and not disease incidence. The relationship between parasite prevalence and the incidence of malaria remains uncertain, and contemporary, high-quality longitudinal data to clarify the relationship between these indicators are limited.

The scale-up of ITN coverage in Uganda (and many other countries in sub-Saharan Africa) has been impressive. Based on national surveys, the proportion of households in Uganda reporting ownership of at least one ITN increased from 16% in 2006 to 60% in 2011 following a series of sub-national distribution campaigns, and then to 90% in 2014 following the universal LLIN distribution campaign [25–27]. The average number of ITNs per household increased from 0.8 in 2009 to 2.5 in 2014 [27]. Although quality surveillance data to estimate the impact of the initial scale-up of ITN provision in Uganda are lacking, there is evidence from our study of a decline in the burden of malaria preceding the universal LLIN campaign in Walukuba. It is unclear what was responsible for this initial decline and why it was seen only in Walukuba, although this may have been influenced by the lower level of transmission intensity and greater urbanization in Walukuba compared to the other two sites [16,28].

Despite gradual improvements in the coverage levels of ITNs over the last 10 y in Uganda, evidence from this study suggests that the universal LLIN distribution campaign was followed by only modest declines in some malaria metrics at our three study sites. There are several potential explanations for these disappointing results. Perhaps of greatest concern is the recent emergence and spread of resistance to pyrethroids, the only class of insecticides available for LLINs. We documented high-level pyrethroid resistance among *A*. *gambiae* s.s. and *A*. *arabiensis* vectors in our study sites, a trend that is occurring across Africa [29]. Estimating the role of pyrethroid resistance in the protective efficacy of LLINs under operational conditions remains a significant challenge. However, increases in malaria cases have been attributed to the emergence of pyrethroid resistance in longitudinal studies from South Africa and Senegal [30,31]. Also of concern are putative changes in vector behavior and shifts in the relative abundance of vector species, which may increase (or leave unchanged) exposure risk during the early evening hours while people are outside of their bed nets and unprotected by LLINs [32,33]. In our study, vector behavioral characteristics were not assessed, and although there were differences in species composition between the three study sites, there was no evidence of a shift in species composition over time at any of the sites. Finally, it is possible that the LLINs were indeed effective, but that the lack of significant changes in malaria metrics was due to inadequate coverage resulting from insufficient numbers of LLINs, loss of nets, or poor compliance. Even after the LLIN distribution campaign, fully universal coverage levels were not achieved. We did not assess LLIN durability and compliance, so we are unable to characterize the contributions of these factors to the outcomes.

Historically, IRS has played a major role in the elimination of malaria in several countries outside of Africa and in greatly reducing the burden of malaria in parts of Africa with low or seasonal transmission [9,34]. However, the evidence base from randomized controlled trials for IRS efficacy when used alone is limited [35]. A number of recent observational studies and cluster randomized trials from Africa comparing the efficacy of IRS combined with ITNs versus either intervention alone have provided mixed results. In observational studies from Equatorial Guinea and Mozambique, IRS combined with ITNs was associated with a lower odds of parasitemia measured in cross-sectional surveys compared to either intervention alone [36]. An analysis of cross-sectional survey data from 17 countries in sub-Saharan Africa indicated that the combination of IRS and ITNs was associated with a lower risk of parasitemia compared to either intervention alone in medium and high transmission areas [37]. In an observational study from western Kenya using a prospective cohort study design, the combination of IRS and ITNs was associated with a lower incidence of new infection compared to ITNs alone [38]. In another observational study from western Kenya, in a setting with high transmission intensity and moderate ITN coverage, two rounds of IRS with pyrethroid insecticides was associated with a lower odds of parasitemia from cross-sectional surveys compared to ITNs alone [39]. In contrast, observational studies from Eritrea and Burundi demonstrated a protective effect of IRS and ITNs when used together, but failed to show any added benefit of the combination compared to either intervention alone [40,41].

Results from cluster randomized trials evaluating ITNs and IRS have also provided conflicting findings. In a study from northwest Tanzania, in a setting with high levels of pyrethroid resistance, 50 clusters received ITNs as part of a universal coverage campaign, and 25 clusters were randomly assigned to additionally receive two rounds of IRS with bendiocarb. The addition of IRS was associated with a significant reduction in parasite prevalence in cross-sectional surveys of children, and there was a non-significant tendency towards a lower entomological inoculation rate [42]. Although reported ITN use was suboptimal in this trial, in subgroup analyses ITNs provided some individual protection and IRS provided additional protection among both net-users and non-users [8]. In contrast, two studies from West Africa failed to show any additional benefit of IRS over ITNs alone. In a high transmission area of Benin with moderate pyrethroid resistance, the addition of IRS with bendiocarb or carbamate-treated plastic sheeting to targeted or universal LLIN coverage did not provide benefit in reducing the incidence of malaria or parasite prevalence in cohorts of children [43]. In a moderate transmission area of the Gambia with susceptible vectors, the addition of IRS with DDT to a background of high LLIN coverage did not provide any benefit in reducing the incidence of malaria, parasite prevalence, or measures of transmission intensity [44]. The results of these studies and our new findings suggest a pattern. Adding IRS to LLINs appears to be most effective in areas where LLIN coverage is low and/or pyrethroid resistance is high. In addition, in areas where LLIN coverage is high, IRS may be most effective when using non-pyrethroid-based insecticides, in line with current WHO recommendations, and as seen in our study.

There were several limitations to our study, and results should be interpreted with caution. The main limitation was the use of an observational study design, comparing outcomes before and after the interventions were implemented. Although we utilized a rigorous analytical approach to adjust for secular trends, the lack of contemporaneous control groups limited our ability to make rigorous causal inferences. In addition, the interventions were implemented under programmatic conditions and not in the setting of a rigorous clinical trial. This is of particular relevance for estimates of changes in malaria metrics following the universal LLIN distribution campaign, which was preceded by other sub-national distribution campaigns. Also these estimates do not include consideration of adherence or net quality. Because cohort participants received LLINs at the start of the study, the cohort study and entomology survey data utilized in this study provided estimates of changes in malaria incidence and HBR following universal LLIN distribution in the setting of existing LLIN ownership. In contrast, data from the health-facility-based surveillance measured direct changes in malaria TPR following universal LLIN distribution. Thus, these measures are complementary. Despite these limitations, our study benefitted from a comprehensive malaria surveillance system focusing on multiple indicators measured longitudinally, including disease incidence, a major improvement compared to cross-sectional surveys measuring only parasite prevalence.

Uganda is representative of several countries in sub-Saharan Africa that have made great strides in reducing the burden of malaria but face substantial challenges on the road to elimination. LLINs and IRS remain the primary interventions for the prevention of malaria in Africa; however, their relative roles in the setting of limited resources are controversial. Like most African countries, Uganda has focused on maximizing coverage with LLINs. Over the last decade, this has led to dramatic increases in coverage levels, culminating in a national universal LLIN distribution campaign resulting in 90% of households owning at least one LLIN and 72% of persons reportedly sleeping under an LLIN [27]. IRS has also become a key component of Uganda’s malaria control strategy, but resource constraints have limited its use to selected areas of the country, with less than 10% of the population protected by IRS, a level similar to that in several other African countries [10]. In this study, following the recent universal LLIN distribution campaign, we found evidence of a modest decline in the burden of malaria at one relatively low transmission site (Walukuba), but no reduction in two higher transmission sites (Kihihi and Nagongera). In contrast to the limited changes following LLIN distribution, three rounds of IRS with a carbamate insecticide at one high transmission site (Nagongera) was followed by a marked decline in the burden of malaria. These data strongly suggest that IRS with non-pyrethroid-based insecticides in combination with LLINs is the most effective intervention currently available for reducing the burden of malaria in Uganda. Resources are needed to increase LLIN coverage and expand the IRS program to cover a greater proportion of the Ugandan population. Given the complex and dynamic nature of vector populations, insecticide resistance patterns, local epidemiology, and the operational effectiveness of malaria control interventions, it is not possible to develop a “one size fits all” approach to malaria control in Africa. More resources are needed to support high-quality malaria surveillance to assess the effectiveness of malaria control interventions over time and space to support evidence-based policy decision making.

## Supporting Information

S1 Fig Profile of households and study participants enrolled into cohort studies and entomology surveys. (EPS)

S2 Fig Linear correlations between relative monthly changes in the incidence of malaria (log_10_) observed in the cohort studies and predicted from the health-facility-based surveillance test positivity rates. The solid line represents the best fit of the data, and the dashed line represents the perfect positive correlation (i.e., slope = 1).
(EPS)

S3 Fig Cumulative relative changes in the incidence of malaria (log_10_) from one month to the next observed in the cohort studies and predicted from the health-facility-based surveillance test positivity rates. Yellow vertical bars indicate when universal distribution of long-lasting insecticidal nets occurred. Pink vertical bars indicate when each round of indoor residual spraying with bendiocarb was implemented. The dashed line indicates no change from baseline (first month of observation).
(EPS)

S1 STROBE Checklist (DOC)

S1 Table Additional details of ARIMA models used for time-series analyses. (DOCX)

S1 Text Cross-sectional survey study protocol. (DOC)

S2 Text Cohort study protocol. (DOCX)

S3 Text Entomology survey study protocol. (DOCX)

S4 Text Grant proposal. (DOC)

## Abbreviations

ACT: artemisinin-based combination therapy
ARIMA: autoregressive integrated moving average
aRR: adjusted rate ratio
HBR: human biting rate
IRS: indoor residual spraying of insecticide
ITN: insecticide-treated net
LLIN: long-lasting insecticidal net
NMCP: National Malaria Control Program
PPY: per person-year
TPR: test positivity rate
WHO: World Health Organization
