# Supplementary material for: Measures of Malaria Burden after Long-Lasting Insecticidal Net Distribution and Indoor Residual Spraying at Three Sites in Uganda: A Prospective Observational Study
Source: PLoS Med. 2016 Nov 8;13(11):e1002167. doi: 10.1371/journal.pmed.1002167 (PMC5100985; doi:10.1371/journal.pmed.1002167)
Supplement: S1 STROBE Checklist — (DOC) [file pmed.1002167.s004.doc]

STROBE Statement—checklist of items that should be included in reports of observational studies

|  | Item No | Recommendation |
| --- | --- | --- |
| **Title and abstract** | 1 | (*a*) Indicate the study’s design with a commonly used term in the title or the abstract  **Response:** the phrase “A Prospective Observational Study” has been included in the title |
|  |  | (*b*) Provide in the abstract an informative and balanced summary of what was done and what was found  **Response:** The methods for the malaria surveillance studies are succinctly described and the main findings included with a balanced summary in the conclusions |
| Introduction | | |
| Background/rationale | 2 | Explain the scientific background and rationale for the investigation being reported  **Response:** Background and rationale provided in paragraphs 1 and 2 of the introduction section. |
| Objectives | 3 | State specific objectives, including any prespecified hypotheses  **Response:** The objectives are summarized in paragraph 3 of the introduction |
| Methods | | |
| Study design | 4 | Present key elements of study design early in the paper  **Response:** Key elements of the study design are provided in paragraphs 3 (health facility-based surveillance), 4 (cohort studies), 5 (entomology surveys), and 6 (cross-sectional surveys) of the methods section. |
| Setting | 5 | Describe the setting, locations, and relevant dates, including periods of recruitment, exposure, follow-up, and data collection  **Response:** The study sites are described in paragraph 2 of the methods section and Figure 1. The dates of data collection for the various surveillance studies are described in paragraphs 3-6 of the methods section. |
| Participants | 6 | (*a*) *Cohort study*—Give the eligibility criteria, and the sources and methods of selection of participants. Describe methods of follow-up  **Response:** Described in paragraph 4 of the methods section.  *Cross-sectional study*—Give the eligibility criteria, and the sources and methods of selection of participants  **Response:** Described in paragraph 6 of the methods section. |
|  |  |  |
| Variables | 7 | Clearly define all outcomes, exposures, predictors, potential confounders, and effect modifiers. Give diagnostic criteria, if applicable  **Response:** Outcomes and exposure variables of interest are defined in paragraphs 2-4 of the subheading “statistical analysis” in the methods section. |
| Data sources/ measurement | 8* | For each variable of interest, give sources of data and details of methods of assessment (measurement). Describe comparability of assessment methods if there is more than one group  **Response:** For outcome variables, sources of date and details of methods of assessment provided in paragraphs 3 (health facility-based surveillance), 4 (cohort studies), and 5 (entomology surveys) of the methods section. For exposure variables, sources of date and details of methods of assessment provided in the subheading “population level malaria control interventions” in the methods section. |
| Bias | 9 | Describe any efforts to address potential sources of bias  **Response:** Efforts to address potential sources of bias are described in paragraphs 2-5 of the subheading “statistical analysis” in the methods section. |
| Study size | 10 | Explain how the study size was arrived at  **Response:** There were no sample/power calculations done for this study. Rather samples sizes for the various surveillance studies were based on available resources and the duration of observation. |
| Quantitative variables | 11 | Explain how quantitative variables were handled in the analyses. If applicable, describe which groupings were chosen and why  **Response:** All quantitative variables were analysed as continuous variables. |
| Statistical methods | 12 | (*a*) Describe all statistical methods, including those used to control for confounding  **Response:** All statistical methods including controlling for confounding are described in paragraphs 2-4 of the subheading “statistical analysis” in the methods section. |
|  |  | (*b*) Describe any methods used to examine subgroups and interactions  **Response:** No subgroup analyses or analyses of interactions were performed. |
|  |  | (*c*) Explain how missing data were addressed  **Response:** There was no missing data as all data included in the analyses come from longitudinal surveillance studies and only observed data were included |
|  |  | (*d*) *Cohort study*—If applicable, explain how loss to follow-up was addressed  **Response:** Not applicable  *Cross-sectional study*—If applicable, describe analytical methods taking account of sampling strategy  **Response:** Not applicable |
|  |  | (*e*) Describe any sensitivity analyses  **Response:** Sensitivity analyses were not performed |

| Results | | |
| --- | --- | --- |
| Participants | 13* | (a) Report numbers of individuals at each stage of study—eg numbers potentially eligible, examined for eligibility, confirmed eligible, included in the study, completing follow-up, and analysed  **Response:** Summarized in Table 1 and Supplemental Figure 1 |
|  |  | (b) Give reasons for non-participation at each stage  **Response:** Included in Supplemental Figure 1 |
|  |  | (c) Consider use of a flow diagram  **Response:** Included in Supplemental Figure 1 |
| Descriptive data | 14* | (a) Give characteristics of study participants (eg demographic, clinical, social) and information on exposures and potential confounders  **Response:** Included in Table 1 |
|  |  | (b) Indicate number of participants with missing data for each variable of interest  **Response:** Not applicable |
|  |  | (c) *Cohort study*—Summarise follow-up time (eg, average and total amount)  **Response:** Included in Table 1 |
| Outcome data | 15* | *Cohort study*—Report numbers of outcome events or summary measures over time  **Response:** Included in Table 1 |
|  |  |  |
|  |  | *Cross-sectional study—*Report numbers of outcome events or summary measures  **Response:** Included in Table 2 |
| Main results | 16 | (*a*) Give unadjusted estimates and, if applicable, confounder-adjusted estimates and their precision (eg, 95% confidence interval). Make clear which confounders were adjusted for and why they were included  **Response:** Included in Table 3 and described in paragraphs 2-5 of the subheading “statistical analysis” in the methods section. |
|  |  | (*b*) Report category boundaries when continuous variables were categorized  **Response:** Not applicable |
|  |  | (*c*) If relevant, consider translating estimates of relative risk into absolute risk for a meaningful time period  **Response:** Not applicable |
| Other analyses | 17 | Report other analyses done—eg analyses of subgroups and interactions, and sensitivity analyses  **Response:** Not applicable |
| Discussion | | |
| Key results | 18 | Summarise key results with reference to study objectives  **Response:** Included in paragraph 1 of the discussion section |
| Limitations | 19 | Discuss limitations of the study, taking into account sources of potential bias or imprecision. Discuss both direction and magnitude of any potential bias  **Response:** Included in paragraphs 7 and 8 of the discussion section |
| Interpretation | 20 | Give a cautious overall interpretation of results considering objectives, limitations, multiplicity of analyses, results from similar studies, and other relevant evidence  **Response:** Included in paragraph 9 of the discussion section. Reference to other studies included in paragraphs 2-6 of the discussion section. |
| Generalisability | 21 | Discuss the generalisability (external validity) of the study results  **Response:** Included in paragraph 9 of the discussion section. |
| Other information | | |
| Funding | 22 | Give the source of funding and the role of the funders for the present study and, if applicable, for the original study on which the present article is based  **Response:** Information include in the on-line submission |

.
