# Supplementary material for: Measures of Malaria Burden after Long-Lasting Insecticidal Net Distribution and Indoor Residual Spraying at Three Sites in Uganda: A Prospective Observational Study
Source: PLoS Med. 2016 Nov 8;13(11):e1002167. doi: 10.1371/journal.pmed.1002167 (PMC5100985; doi:10.1371/journal.pmed.1002167)
Supplement: S1 Table — (DOCX) [file pmed.1002167.s005.docx]

**Supplemental Table 1. Additional details of ARIMA models used for time-series analyses**

| **Example of candidate ARIMA models and AIC for Walukuba for TPR** | | |
| --- | --- | --- |
| **ARIMA(p,d,q)(P,D,Q)_m_ ^1^** | **Predictor (lag in months)** | **AIC** |
| (2,1,1)(1,1,1)_6_ | ITN, rainfall (1) (**FINAL MODEL**) | 21.1 |
| (2,1,1)(1,1,1)_6_ | ITN, rainfall (1), age, proportion female | 21.9 |
| (1,1,1)(0,1,0)_6_ | ITN, rainfall (1), age | 22.0 |
| (0,1,0)(0,0,0)_6_ | ITN | 37.4 |
| (1,1,0)(0,0,0)_6_ | ITN, rainfall (1) | 39.4 |
| **Final ARIMA models** ^2^ | | |
| **Study site** | **Malaria metric** | **ARIMA(p,d,q)(P,D,Q)_m_** **^1^** |
| **Walukuba** | Test positivity rate | (2,1,1)(1,1,1)_6_ |
|  | Incidence of malaria | (4,1,1)(0,1,1) _6_ |
|  | Human biting rate | (1,1,1)(1,1,0)_6_ |
| **Kihihi** | Test positivity rate | (1,1,0)(1,1,0)_6_ |
|  | Incidence of malaria | (3,1,1)(0,0,1)_6_ |
|  | Human biting rate | (2,1,1)(1,1,0)_6_ |
| **Nagongera** | Test positivity rate  ITN model  IRS model | (3,1,1)(1,1,0)_6_  (1,1,1)(1,1,1)_6_ |
|  | Incidence of malaria  ITN model  IRS model | (0,1,1)(0,0,0)_6_  (0,1,1)(0,0,0)_6_ |
|  | Human biting rate  ITN model  IRS model | (2,1,1)(1,1,0)_6_  (2,1,1)(0,1,0)_6_ |

^1^ p=autoregressive order, d=differencing order, q=moving average order; *m* refers to the number of periods in each season, and the uppercase P,D,Q refer to the autoregressive, differencing, and moving average terms for the seasonal part of the ARIMA model.

^2^ All models adjusted for rainfall with a one-month lag
