# Supplementary material for: Measures of Malaria Burden after Long-Lasting Insecticidal Net Distribution and Indoor Residual Spraying at Three Sites in Uganda: A Prospective Observational Study
Source: PLoS Med. 2016 Nov 8;13(11):e1002167. doi: 10.1371/journal.pmed.1002167 (PMC5100985; doi:10.1371/journal.pmed.1002167)
Supplement: S2 Text — (DOCX) [file pmed.1002167.s007.docx]

**“Program for resistance, immunology, surviellance, and modelling of malaria”**

**(PRISM)**

**Study Title: Cohort studies to estimate malaria incidence and morbidity in three different epidemiological settings in Uganda**

**DMID Protocol Number:** 10-0063

**Sponsored by:** National Institute of Allergy and Infectious Diseases (NIAID)

**DMID Funding Mechanism:** NIH/NIAID U19AI089674

**Principal Investigator:** Moses R. Kamya, MBChB, MPH, PhD

**DMID Program Officer:** Malla Rao, DrPH, M Engg

**Draft Number:** 1.0, dated: 10 March 2011

**Statement of Compliance**

The study will be carried out in accordance with Good Clinical Practice (GCP) as required by the following:

- US Code of Federal Regulations applicable to Clinical Studies (45 CFR)
- *Ugandan Ethics Committee* (or whatever constitutes the national regulatory body)
- Completion of Human Subjects Protection Training
- NIH/NIAID Clinical Terms of Award

**SIGNATURE PAGE**

The signature below constitutes the approval of this protocol and the attachments, and provides the necessary assurances that this study will be conducted according to all stipulations of the protocol, including all statements regarding confidentiality, and according to local legal and regulatory requirements and applicable US federal regulations and ICH guidelines.

Site Investigator:

Signed: ______________________________________________

*Name*

*Title*

TABLE OF CONTENTS

[PARTICIPATING SITES 4](#_Toc284600999)

[INSTITUTIONAL REVIEW BOARDS 5](#_Toc284601000)

[INVESTIGATORS 6](#_Toc284601001)

[STUDY SYNOPSIS 8](#_Toc284601002)

[ABBREVIATIONS AND ACRONYMS 9](#_Toc284601003)

[1.0 BACKGROUND 10](#_Toc284601004)

[1.1 Burden of malaria in sub-Saharan Africa and Uganda 10](#_Toc284601005)

[1.2 Malaria control in Sub-Saharan Africa and Uganda 10](#_Toc284601006)

[1.3 Monitoring the burden of malaria in Uganda 11](#_Toc284601007)

[2.0 STUDY RATIONALE 12](#_Toc284601008)

[3.0 STUDY OBJECTIVES 13](#_Toc284601009)

[3.1 Primary Objective 13](#_Toc284601010)

[3.2 Secondary objectives 13](#_Toc284601011)

[4.0 METHODS 13](#_Toc284601012)

[4.1 Study design 13](#_Toc284601013)

[4.2 Study sites 13](#_Toc284601014)

[Table 1. Characteristics of selected districts 15](#_Toc284601016)

[4.3 Study participants 15](#_Toc284601017)

[4.3.1 Selection and Initial contact 16](#_Toc284601018)

[4.3.2 Study participant screening 16](#_Toc284601019)

[4.3.3 Informed consent 17](#_Toc284601020)

[4.3.4 Study enrolment 17](#_Toc284601021)

[4.4 Study follow-up and procedures 18](#_Toc284601022)

[4.4.1 Clinic visits 18](#_Toc284601023)

[4.4.2 Malaria diagnosis and treatment 19](#_Toc284601024)

[Table 2 below provides a summary of the schedule for blood collection 20](#_Toc284601025)

[4.4.3 Non-malarial illnesses 20](#_Toc284601026)

[4.4.4 Administration of medications 21](#_Toc284601027)

[4.5 Criteria for study discontinuation 21](#_Toc284601028)

[4.6 Laboratory methods 21](#_Toc284601029)

[4.6.1 Microscopy 21](#_Toc284601030)

[4.6.2 Haemoglobin measurement 21](#_Toc284601031)

[4.6.3 Molecular and Immunology Studies 22](#_Toc284601032)

[5.1 Records to be kept 22](#_Toc284601033)

[5.2 Data quality assurance and monitoring 23](#_Toc284601034)

[5.3 Record keeping 23](#_Toc284601035)

[5.4 Statistical considerations and analysis 23](#_Toc284601036)

[Table 3. Outcome measures 23](#_Toc284601037)

[Table 4. Malaria control intervention variables of interest 25](#_Toc284601038)

[6.0 HUMAN SUBJECTS 27](#_Toc284601039)

[6.1 Risks and Discomforts 27](#_Toc284601040)

[6.2 Costs to the Subjects 27](#_Toc284601041)

[6.3 Reimbursement of Subjects 27](#_Toc284601042)

[6.4 Informed consent 28](#_Toc284601043)

[6.5 Confidentiality 28](#_Toc284601044)

[6.6 Institutional review boards (IRBs) 28](#_Toc284601045)

[REFERENCES 29](#_Toc284601046)

[Appendix A. HOUSEHOLD QUESTIONNAIRE 30](#_Toc284601047)

[Appendix B. WHO CRITERIA FOR SEVERE MALARIA/DANGER SIGNS 31](#_Toc284601048)

# PARTICIPATING SITES *(contact information redacted)*

**Uganda Malaria Surveillance Project (UMSP)**

Address: Infectious Disease Research Collaboration (IDRC),

Mulago Hospital Complex, MUJHU 3 Building, P.O. Box 7475,

Kampala, Uganda

Contact Person: Catherine Tugaineyo

Phone Number: +256 (0) 414-530692

Fax Number: +256 (0) 414-540524

Email: ctugaineyo@muucsf.com

**London School of Hygiene & TropicalMedicine (LSHTM)**

Address: Keppel Street, London,WC1E 7HT, UK

Contact Person: Susan Sheedy

Phone Number: +44 (0) 20 7927 2256

Fax Number: +44 (0)20 7637 4314

Email: susan.sheedy@lshtm.ac.uk

**University of California, San Francisco (UCSF)**

Address: San Francisco General Hospital, 1001 Potrero Avenue,

Building 30, Room 402/408, San Francisco, CA 94110, USA

Contact Person: Tamara Clark

Phone Number: +1 (415) 206-8790

Fax Number: +1 (415) 648-8425

Email: tclark@medsfgh.ucsf.edu

# INSTITUTIONAL REVIEW BOARDS *(contact information redacted)*

**Uganda National Council for Science and Technology (UNCST)**

Address: Uganda House, 11^th^ Floor, PO Box 6884, Kampala, Uganda

Contact Person: Dr. Thomas Gordon Egwang

Phone Number: 256-41-250499

Fax Number: 256-41-234579

**Makerere University, School of Medicine Research and Ethics Committee (SOMREC)**

Address: Makerere University, Faculty of Medicine, Office of the Dean,

PO Box 7072, Kampala, Uganda

Contact Person: Dr. Charles Ibingira

Phone Number: +256 (0) 414-530020

Fax Number: +256 (0) 414-531091

**University of California, San Francisco, Committee for Human Research (UCSF-CHR)**

Address: Office of Research, 3333 California Street Suite 315, San

Francisco, CA 94118, U.S.A.

Contact Person: Dr. Reese T. Jones

Phone Number: 01-415-476-1814

Fax Number: 01-415-502-1347

**London School of Hygiene and Tropical Medicine Ethics Committee**

Address: Keppel Street, London, WC1E 7HT, UK

Contact Person: Gemma Howe

Phone Number: +44 (0) 20 7927 2802

Email: Ethics@lshtm.ac.uk

# INVESTIGATORS

Moses Kamya, MBChB, MPH, PhD

Role in project: Principal Investigator

Professor, Department of Medicine, Makerere University, Kampala, Uganda

Director, Infectious Disease Research Collaboration / Uganda Malaria Surveillance

Project, Kampala, Uganda

Email: [mkamya@infocom.co.ug](mailto:mkamya@infocom.co.ug)

Grant Dorsey, MD, PhD

Role in project: Co-investigator

Associate Professor, Department of Medicine, University of California, San Francisco

Co-investigator, Uganda Malaria Surveillance Project, Kampala, Uganda

Email: [gdorsey@medsfgh.ucsf.edu](mailto:gdorsey@medsfgh.ucsf.edu)

Sarah Staedke, MD, PhD

Role in project: Co-investigator

Clinical Senior Lecturer, London School of Hygiene and Tropical Medicine, London, UK

Co-investigator, Uganda Malaria Surveillance Project, Kampala, Uganda

Email: [sarah.staedke@lshtm.ac.uk](mailto:sarah.staedke@lshtm.ac.uk)

Anne Gasasira, MBchB, PhD

Role in project: Co-investigator

Epidemiologist, Uganda Malaria Surveillance Project, Kampala, Uganda

Email: [agasasira@muucsf.org](mailto:agasasira@muucsf.org)

Adoke Yeka, MBchB, Msc

Role in project: Co-investigator

Epidemiologist, Uganda Malaria Surveillance Project, Kampala, Uganda

Email: [yadoke@yahoo.com](mailto:yadoke@yahoo.com)

Humphrey Wanzira, MBchB, Msc

Role in project: Co-investigator/coordinator

Epidemiologist, Uganda Malaria Surveillance Project, Kampala, Uganda

Email: [wanzirah@yahoo.com](mailto:wanzirah@yahoo.com)

Chris Drakeley, BSc, PhD

Role in project: Co-investigator

Department of Immunology and Infection, London School of Hygiene and Tropical Medicine, London, UK

Email: [chris.drakeley@lshtm.ac.uk](mailto:chris.drakeley@lshtm.ac.uk)

Teun Bousema

Role in project: Co-investigator

Department of Immunology and Infection, London School of Hygiene and Tropical Medicine, London, UK

Email: [teun.bousema@lshtm.ac.uk](mailto:teun.bousema@lshtm.ac.uk)

Bryan Greenhouse, MD

Role in Project: Co-Investigator

Assistant Adjunct Professor, University of California, San Francisco

Co-Investigator MU-UCSF research Collaboration

Email: [bgreenhouse@medsfgh.ucsf.edu](mailto:bgreenhouse@medsfgh.ucsf.edu)

Arthur Mpimbaza, MBchB, MMed

Role in the project: Collaborator

Pediatrician, Uganda Malaria Surveillance Project, Kampala, Uganda

Email: arthurwakg@yahoo.com

Joaniter Nankabirwa, MBchB, MSc

Role in the project: Collaborator

Epidemiologist, Uganda Malaria Surveillance Project, Kampala, Uganda

Email: [jnankabirwa@yahoo.co.uk](mailto:jnankabirwa@yahoo.co.uk)

**OTHER KEY STUDY PERSONNEL**

| Name | Title |
| --- | --- |
| TBH | Cohort coordinator – Nagongera |
| TBH | Cohort coordinator – Walukuba |
| TBH | Cohort coordinator – Kihihi |
| Geoff Lavoy | Data manager |
| Ruth Kigozi | Assistant data manager |
| TBH | Clinical officer – Nagongera |
| TBH | Clinical officer – Walukuba |
| TBH | Clinical officer – Kihihi |

# STUDY SYNOPSIS

| Title | **Cohort studies to estimate malaria incidence and morbidity in three different epidemiological settings in Uganda** |
| --- | --- |
| Description | Three concurrent dynamic cohort studies in settings with varying malaria transmission intensities will be conducted to measure the incidence of malaria and indicators of malaria morbidity |
| Study sites | Study participants will be enrolled from three sub-counties in Uganda: Nagongera sub-county in Tororo District, Walukuba sub-county in Jinja District and Kihihi sub-county in Kanungu District. Study clinics will be located at the health center IV facilities serving each of the catchment areas, also utilized as malaria sentinel sites. |
| Study objectives | 1. To estimate the incidence of malaria among children and adults living in three different epidemiological settings with varying levels of malaria transmission intensity. 2. To measure the prevalence of asymptomatic parasitaemia and anaemia among children and adults living in three settings of varying malaria transmission intensity. 3. To determine the age- and exposure-dependent acquisition of antibody responses to malaria antigens 4. To measure associations between antimalarial antibodies and protection from malaria. |
| Participants and Sample Size | Each cohort will enroll all children aged 6 months to 10 years and their primary care givers from 100 randomly selected households in the three catchment sub-counties. |
| Selection Criteria | Eligibility criteria of potential study participants include:   1. Documented age between 6 months to 10 years OR an adult (≥18 years) primary care giver of at least one child who is enrolled in the cohort 2. Resident in the sub-county at the household selected for recruitment. 3. No intention to move out of the sub-county for the next two years. 4. Agreement to come to the study clinic at the UMSP sentinel site for any febrile illness. 5. Agreement to avoid antimalarial medications administered outside the study. 6. Provision of written informed consent (from parent or guardian in case of children). 7. Absence of a chronic medical condition requiring specialized primary health care 8. Not enrolled in another research study |
| Participant  Follow up | Study participants will be followed for two years for all their health care needs at designated study clinics. Study participants with suspected malaria will undergo standard evaluation for malaria. Those diagnosed with malaria will be prescribed artemether-lumefantrine. Study participants will be seen for routine visits every three months to assess for asymptomatic parasitemia and anemia, collect blood for immunology and molecular studies and reinforce the study protocol. |
| Malaria Case definitions | **Uncomplicated malaria:** all of the following   1. Documented fever (> 38.0°C tympanic ) or history of fever in the previous 24 hrs 2. Positive thick blood smear 3. Absence of complicated malaria   **Complicated malaria (any of the following):**  1. Evidence of severe disease with a positive thick blood smear  2. Danger signs with a positive thick blood smear  3. Parasite density > 500,000/ul |
| Primary study outcome | Malaria incidence density |
| Secondary outcomes | Prevalence of asymptomatic parasitemia  Prevalence of anaemia |

# ABBREVIATIONS AND ACRONYMS

ACT Artemisinin-based Combination Therapy

CRF Case Record Form

DHS Demographic and health surveys

GCP Good Clinical Practice

GFATM Global Fund to Fight AIDS, Tuberculosis and Malaria

HMIS Health Management Information System

IPTp Intermittent Preventive Therapy in Pregnancy

LLITN Long-Lasting Insecticidal Treated Net

MIS Malaria Indicator Survey

PMI President’s Malaria Initiative

QA Quality Assurance

UMSP Uganda Malaria Surveillance Project

WHO World Health Organization

# 1.0 BACKGROUND

## 1.1 Burden of malaria in sub-Saharan Africa and Uganda

Malaria remains a major public health problem in much of sub-Saharan Africa. It is responsible for 10% of the overall disease burden, 30-50% of inpatient admissions, up to 50% of outpatient visits and 40% of the total public health expenditure in this region (www.rbm.who.int). The populations at highest risk of malaria are young children and pregnant women^1^.

Uganda, where the proposed work would be located, is emblematic of the immense problem that malaria poses for African countries. According to the 2008 World Malaria Report, Uganda ranked 6^th^ in terms of number of malaria cases and 3^rd^ in terms of number of malaria deaths^2^. Malaria is the leading cause of morbidity and mortality in Uganda. It is responsible for up to 40% of all outpatient visits, 25% of all hospital admissions and 14% of all hospital deaths (Uganda Ministry of Health, unpublished). The malaria-specific mortality in children is estimated to be between 70,000 and 100,000 deaths per year, a death toll that far exceeds that of HIV/AIDS ^3^. In 2009, a countrywide malaria indicator survey reported that 42% of children aged 0-59 months were infected with malaria parasites ^5^.

Although over 90% of Uganda’s population live in endemic areas, the epidemiology of malaria varies widely across the country ranging from low and unstable transmission, predominantly in highland areas, to some of the highest transmission intensities reported in the world with year round transmission^4^. This variability was illustrated in the 2009 malaria survey which found substantial differences in infection rates across geographic regions ranging from 4.9% parasitemia in children living in the central region to 62.5% in those in the mid-northern region ^5^.

## 1.2 Malaria control in Sub-Saharan Africa and Uganda

Commitment by the global community to decrease the burden of malaria has recently escalated, with concentrated efforts in sub-Saharan Africa. In 2005, the Roll Back Malaria Partnership and World Health Assembly set a target to reduce the number of malaria cases by 50% between 2000 and the end of 2010 and by 75% by the end of 2015^6^.

In order to meet these targets there has been a rapid scale up of proven interventions, notably distribution of long lasting insecticide treated bed nets (LLITNs), indoor residual spraying (IRS) with pesticides, intermittent preventive therapy in pregnant women (IPTp) and prompt and effective treatment with highly efficacious artemisinin combination therapies (ACT). Subsequently, some endemic African countries like Kenya, Tanzania, Zanzibar, Ethiopia and most of the South African countries have reported impressive reductions in malaria incidence^7,8^.

Uganda like other sub-Saharan countries is a beneficiary of increased efforts to control malaria. Through large funding sources such as the Global Fund to Fight AIDS, Tuberculosis and Malaria (GFATM) and the United States government President’s Malaria Initiative (PMI), Uganda now has an unprecedented opportunity to reduce malaria associated morbidity and mortality on a national scale. Indeed increased resources have been followed by widening deployment of key intervention strategies. At least 3 million insecticide treated bednets, the main strategy for malaria prevention in Uganda, have been distributed since 2006, increasing the overall household coverage from 34% in 2006 to 59% in 2009 and coverage in pregnant women and children under five by 23% and 33% respectively. Similarly, treatment of fevers with an ACT increased from 1% to 14% in children under 5 over the same period^5^. However, there is limited data to show that these increases have impacted the malaria burden in Uganda. On the contrary, anecdotal data show no improvement in the malaria situation in the country^7^. There remains optimism that the tide can be turned through the further expansion of these interventions. However, there is a critical need for an evidence base to support the targeted use of control interventions to maximize their impact. The process of documenting this transitioning epidemiology of malaria can only be achieved through a robust framework of malaria surveillance using validated metrics of disease burdens.

## 1.3 Monitoring the burden of malaria in Uganda

Most malaria endemic countries rely heavily on health facility-based surveillance to monitor the malaria burden. In this surveillance system, cases diagnosed as malaria at health facilities are reported through a national system and trends are monitored over time.

In Uganda, malaria cases are reported through a Health Management Information System (HMIS) and analysed at national level. While these data are collected routinely from presumably the majority of health facilities in the country, they are often limited due to incomplete reporting, failure of the system to capture cases that occur outside the formal health care system, and the absence of laboratory confirmation, particularly where the vast majority of reported cases of malaria are not laboratory confirmed ^9,10^. As a result these data are grossly sub-optimal to monitor the changing clinical epidemiology of malaria in the setting of increasing intervention coverage. Indeed, national reports on trends in malaria cases from Uganda derived from the HMIS system often simply reflect the number of cases of fever captured through HMIS^2^.

A practical solution to improving health facility data is to identify key elements needed to ensure accurate surveillance and implement at selected sites. This is the basis of health facility-based sentinel surveillance. The Uganda Malaria surveillance project (UMSP) runs a sentinel surveillance system at health facilities located in different parts of the country. Sentinel sites, chosen to represent the geographical and epidemiological diversity around the country, consist of 6 level IV health facilities: 2 located in districts with relatively low malaria transmission intensity (Kabale and Kanungu), 2 in districts with medium transmission intensity (Mubende and Jinja), and 2 in districts with high transmission intensity (Apac and Tororo). Morbidity data from the sites is analysed monthly to monitor malaria trends.

Another source of malaria data in Uganda is periodic national surveys. Population-based demographic and health surveys, such as a recently concluded Malaria Indictor Survey in Uganda, provide useful statistics on a range of malaria indicators from nationally representative household samples. However they are very expensive to conduct, making them unfavourable to conduct frequently and unattractive to rely on for routine malaria surveillance in a resource limited setting.

The best metric for measuring malaria disease burden is malaria incidence. Therefore a good surveillance program should aim at estimating the number of incident cases originating from a defined catchment population base. This is however challenging to routinely enumerate as the most accurate method involves well designed longitudinal studies in which a representative well described population sample is followed through time and incident cases systematically identified. These cohort studies minimize undetected cases that often limit other methods of estimating malaria trends, utilize valid case definitions and standardized methodology of case ascertainment and often collect useful data beyond malaria counts. However they are labor-intensive, require specialized skills and not implementable over a wide enough geographical scale for routine surveillance purposes. Longitudinal studies may therefore provide gold-standard method for assessing malaria incidence against which other existing methodologies are compared.

# 2.0 STUDY RATIONALE

Several countries in sub-Saharan Africa have reported declines in the burden of malaria with increasing coverage of key malaria interventions. In Uganda, however, no such change has been clearly demonstrated and as noted above, country level statistics are largely based on limited and poor quality data. There has been very little scientific effort in Uganda to document any transitioning epidemiology of malaria using validated metrics of disease burdens within a robust framework of surveillance and there are very few contemporary studies that have tested the reliability of the current national surveillance activities ^11^. It is therefore not clear if the overall malaria burden is changing at all, if there are changes in some but not other transmission settings and how any changes may be correlated to intervention coverage.

We propose to conduct three prospective cohorts to measure the incidence of malaria over time in three sub-counties in Uganda. These studies will provide accurate current information on the burden of malaria in a low-transmission setting, a medium transmission setting and a very high transmission setting. The catchment areas of these studies will overlap with those of three existing sentinel surveillance sites allowing us to compare metrics from the proposed studies to those obtained through the sentinel system.

# 3.0 STUDY OBJECTIVES

## 3.1 Primary Objective

To estimate the incidence of malaria among children and adults living in three different epidemiological settings with varying levels of malaria transmission intensity.

## 3.2 Secondary objectives

1. To measure the prevalence of asymptomatic parasitaemia and anaemia among children and adults living in three settings of varying malaria transmission intensity.
2. To determine the age- and exposure-dependent acquisition of antibody responses to malaria antigens
3. To measure associations between antimalarial antibodies and protection from malaria.

# 4.0 METHODS

## 4.1 Study design

We propose to conduct three concurrent, dynamic population-based cohort studies to estimate the incidence of malaria and other indicators of malaria morbidity in children living in three well defined epidemiological settings of varying malaria transmission intensities. Study participants will be recruited from randomly selected households located in our catchment areas and followed for two years.

**4.2 Study sites**

The studies will be conducted in three sub-counties located in Uganda: Nagongera sub-county, located in Tororo district in Eastern Uganda, Walukuba sub-county, located in Jinja district in Eastern Uganda and Kihihi sub-county, located in Kanungu district in south-western Uganda. Study clinics will be set up at the UMSP sentinel site health center IV facilities which serve each of the sub-counties (Table 1).

Nagongera sub-county, is predominantly a rural setting considered to be of very high malaria transmission. Entomology studies conducted in Nagongera in 2001-02 estimated the entomological inoculation rate (EIR) to be 562 infective bites per person per year ^4^. Recent demographic data from this sub-county comes from a survey done by our group in 2009 and data from our ongoing sentinel surveillance project. The total population of Nagongera sub-county is 37,714, with children aged 1 to 10 years constituting 37% of the population. Nagongera Health Center IV is the largest public health facility in the sub county and treated an average of 2044 patients per month in 2010 (umsp.muucsf.org).

Also located in the eastern part of the country, Walukuba is a peri-urban sub-county in Jinja district. The district, with an estimated population of 387,600, is an area of medium malaria transmission with an estimated EIR of 6 infective bites per person per year ^4^. The Walukuba health center IV is the largest public health facility in the sub-county and treated an average of 3,198 patients per month in 2010 (umsp.muucsf.org).

Kihihi is a rural sub-county in Kanungu district. The district has an estimated population of 204700 and is an area of relatively low malaria transmission intensity with an EIR estimated to be 6 infective bites per person per year ^4^. The largest healthcare point in Kihihi sub-county is Kihihi health center IV, a public health facility that treated an average of 1,945 patients per month in 2010 (umsp.muucsf.org)

## Table 1. Characteristics of selected districts

| **Characteristics** | **DISTRICT** | | |
| --- | --- | --- | --- |
|  | **Kanungu** | **Jinja** | **Tororo** |
| **Demographics** ^a^ | | | |
| Location | Southwest | East | East |
| Population | 204,700 | 387,600 | 536,900 |
| Urbanization level | 6.3% | 22.1% | 6.5% |
| Number of households | 43,500 | 84,000 | 112,300 |
| Persons per household | 4.7 | 4.5 | 4.8 |
| **Entomology (data collected 2001-2002)** | | | |
| Entomologic inoculation rate | 6 | 6 | 562 |
| Predominate vector species | *An. gambiae ss* | *An.gambiae ss* | *An.gambiae ss* |
| **Measures of infection (data collected in children 2-9 years of age, 1999)** | | | |
| Parasite rate | 43% | 15% | 91% |
| **Malaria control interventions** | |  |  |
| IRS, year (% targeted coverage) | 2007 (99%) | None | None |
| ITN coverage (< 5 yrs)^d^ | 6.5% | 5.8% | 12.8% |
| > 2 doses of IPTp at ANC visit^d^ | 24.6% | 14.0% | 15.7% |
| Prompt treatment of fever (< 5 yrs) ^d^ | 23.8% | 22.0% | 31.7% |
| ACT coverage^e^ | 81% | 55% | 43% |
| **Outpatient level IV health facility data (UMSP data December 2009 – May 2010)** | | | |
| Name of health facility | Kihihi | Walukuba | Nagongera |
| Average Number of patients per month | 2117 | 3048 | 1891 |
| Proportion with suspected malaria | 73% | 55% | 73% |
| Slide positivity rate (< 5 yrs) | 47% | 44% | 69% |
| Slide positivity rate (> 5 -15 yrs) | 45% | 48% | 48% |

^a^ Uganda Population and Housing Census, 2002 (Ugandan Bureau of Statistics)

^b^ Data only available from September – November 2008

^d^ Uganda Demographic and Health Survey, 2006

^e^ Proportion of patients with malaria prescribed an ACT at UMSP sentinel health facilities (December 2008-May 2009)

## 4.3 Study participants

We will recruit all children aged 6 months to 10 years from 100 randomly selected households within the catchment sub-counties. Primary caretakers of these children will also be enrolled. For all household members who are not enrolled in the cohorts, information will be collected on a single time-point without scheduled follow-up visits. A household will be defined as any single permanent or semi-permanent dwelling structure acting as the primary residence for a person or group of people that generally cook and eat together.

In order to create a sampling frame for the selection of participating households, all households within each of the three sub-counties will be enumerated and mapped. Identified households will have their coordinates measured using hand-held global positioning systems and assigned unique identification numbers. Details of the enumeration surveys are described in a separate protocol.

Using a computerized number generator, a random sample of households in each sub-county will be selected for screening and study participant recruitment. We will approach households from the randomization list consecutively and enroll all children from a single household who full fill the study criteria along with their primary caretaker(s). The cohorts will be dynamic such that over the course of the 2 year study all newly eligible children will be enrolled (i.e. those that reach 6 months of age) while study participants who reach 11 years of age will be excluded from further follow-up.

### 4.3.1 Selection and Initial contact

Study personnel will approach households on the randomly selected household list in the order in which they were selected. When a household with at least one child of appropriate age is identified, study personnel will briefly describe the purpose of the study to parent(s) or guardian(s). If the parent(s)/guardian(s) are interested in the study, the study personnel will take the potential participant and parent/guardian to the study clinic for screening or schedule a screening appointment date. Households in which no children between the ages of 6 months and 10 years reside will be deleted from the random list and the next listed household visited. If a parent/guardian is not home during the initial contact, the household will be re-visited on at least 3 other occasions over a 2-week period before elimination from the sample selection process. We will enroll a total of 100 households with at least one child and primary caregiver per sub-county.

### 4.3.2 Study participant screening

The screening visit and all subsequent study visits will take place at a designated study clinic in the health center IV facilities located in each catchment area. A study clinician will assess eligibility of potential study participants using the following selection criteria.

Inclusion criteria:

1. Documented age between 6 months to 10 years or a primary caretaker of such a child
2. Resident in the sub-county at the household selected for recruitment
3. No intention to move out of the sub-county for the next two years
4. Agreement to come to the study clinic at the UMSP health center sentinel site for any febrile illness
5. Agreement to avoid antimalarial medications administered outside the study
6. Provision of written informed consent or consent from parent or guardian in case of enrolment of a child; from the participant in case of enrolment of an adult primary care taker

Exclusion criteria:

1. Presence of a chronic medical condition requiring specialized primary health care
2. Enrolled in another research study

### 4.3.3 Informed consent

Written informed consent will be requested from parents/guardians of children as well as the primary care givers of children enrolled who meet the eligibility criteria and assent will be requested from children aged 8 years and older. If parents/guardians are undecided about consenting for their child(ren) or themselves at the time of the initial screening visit, they will be allowed up to two weeks to make a final decision about study participation.

Written informed consent forms will be provided to the parents/guardians and available in Luganda, Lusoga for those in Walukuba; Rukiga, Runyankore for those in Kihihi and Japadhola and Ateso for those in Nagongera sub-county. Following the informed consent discussion, parents/guardians will be asked to sign an IRB-approved written consent form for their child/children and themselves to participate in the research study and a second approved consent form for the future use of biological specimens obtained during the course of the study.

A study clinician will conduct the informed consent discussion at the study clinic in the appropriate language and a translator will be used if necessary and will include a detailed description of the purpose of the study, study procedures and risks and benefits to participants. Each section of the consent form should be read to the participant or parent/guardian exactly as it is written either by the study clinician or by the translator if the study clinician cannot read that language, and then further explained to the participant or parent/guardian if necessary. The translator will also assist with the discussion and assessment of comprehension. If the parent or guardian is unable to read or write, their fingerprint will substitute for a signature, and a signature from a witness to the informed consent procedures will be obtained. Study participants aged 8 – 10 yrs will be asked to provide assent to participate in the study as per Uganda National Council of Science and Technology guidelines ^12^.

In addition to written informed consent for cohort study participants, verbal consent will be requested from all other individuals living in participating households but not eligible for the study, for a single finger-prick blood sample collected only at baseline. This finger prick blood sample will be used for future genetic testing (see section 4.8).

### 4.3.4 Study enrolment

After cohort participants are deemed eligible for enrolment and informed consent for study participation is obtained, they shall be enrolled. Study participants will be given a unique study number which will be linked to their household ID number and will undergo a standard baseline evaluation. The baseline evaluation will include a standard history taking and physical examination accompanied with filling out standard case record forms. Subjects will have blood collected by venipuncture (5-10 cc’s) for hemoglobin measurement, thick blood smear, immunological studies and filter paper sample for future molecular studies. Subjects found to have a fever (tympanic temperature > 38.0°C) or history of fever in the previous 24 hrs and a positive blood smear will be diagnosed with malaria and treated with artemether-lumefantrine while those with other illnesses will be treated according to local guidelines (see sections 4.5 and 4.6).

At the end of the enrolment visit all study participants will be given a long lasting insecticide treated bed net (ITN) and a household survey appointment will be scheduled to collect household-level information on the use of malaria interventions and treatment seeking practices (Appendix A). All individuals living in participating households but not enrolled into the cohort studies will be have a finger-prick blood sample collected on filter paper in order to complete our understanding of genetic risk factors for malaria and age-dependent acquisition of antibody responses in the households from which cohort participants are recruited.

## 4.4 Study follow-up and procedures

Parents/guardians will be asked to bring themselves and their child/children to the study clinics for all their medical care and to avoid the use of any other anti-malarial medication outside of the clinic. Study clinics will be open from Monday to Sunday from 8.00 am to 5:00 pm. Visits to the clinic will be classified as either scheduled routine visits or sick visits. Sick visits will further be categorised as initial or follow up visits.

**4.4.1 Clinic visits**

**Routine visits:** These will be visits conducted at the study clinic every 3 months to ensure protocol compliance and obtain regularly scheduled clinical and laboratory measurements. At each visit a standardized evaluation will be done accompanied with filling out standard case record forms. Parents/guardians will be asked about visits to outside health facilities, use of any anti-malarial medications outside the study protocol and ITN usage. The study protocol will be reinforced with discussion regarding the need to come to the study clinic promptly upon the onset of any illness and to avoid use of outside anti-malarial medications. All participants will have a finger prick done for a thick blood smear to assess for asymptomatic parasitemia, measure haemoglobin levels, and for filter paper samples, and to collect plasma. In addition, among selected participants, a venipuncure will be done at alternate routine visits (6 monthly) to collect blood for immunology assays.

Study participants found to be ill during a routine visit will undergo evaluation using the guidelines for sick visits below. Study subjects who do not present to the clinic for their routine appointments will be visited at home and requested to come to the study clinic as soon as possible.

**Sick Visits:** Initial sick visits will be defined as study visits for a new medical illness. All patients presenting for an initial visit will undergo a standardized clinical history and physical examination. Subjects found to have a fever (tympanic temperature > 38.0°C) or history of fever in the previous 24 hrs will have blood obtained by finger-prick for an urgent blood smear and filter paper sample collection. If the thick blood smear is positive, the patient will be diagnosed with malaria and managed as described in section 4.4.2 below. If the thick blood smear is negative, the patient will be managed as described in section 4.4.3 below. Patients who do not have fever and are not suspected to have malaria will receive standard-of-care treatment as per local treatment guidelines.

Follow-up visits for a previously diagnosed illness will be scheduled as per the clinicians’ discretion. During follow-up visits, blood smears will be done for subjects who present with fever and if the blood smear is positive, the patient will be managed as described in section 4.4.2 below.

**4.4.2 Malaria diagnosis and treatment**

Patients with suspected malaria, defined as fever (tympanic temperature > 38.0°C) or history of fever in the previous 24 hrs will undergo a standardized evaluation as described above. Those found to have malaria based on laboratory confirmation will have a second finger prick for a thin smear and hemoglobin measurement and will be classified as having uncomplicated or complicated malaria based on the following criteria:

**Uncomplicated malaria (all of the following):**^13^

1) Fever (> 38.0°C tympanic) or history of fever in the previous 24 hours

2) Positive thick blood smear

3) Absence of complicated malaria.

**Complicated malaria (any of the following):** ^13^

1. Evidence of severe disease (see Appendix B) with a positive thick blood smear

2. Danger signs in children < 5 years of age (see Appendix B) with a positive thick blood smear

3. Parasite density > 500,000/ul

Episodes of malaria will also be classified into the following categories according to the timing of previous malaria episodes for treatment purposes:

1. New episodes of malaria will be defined as any first episode or any episode occurring > 14 days after the diagnosis of a previous episode
2. Treatment failures will be defined as any of the following:
   1. Complicated malaria occurring 1-14 days after the diagnosis of a previous episode
   2. Fever (> 38.0°C tympanic) or history of fever in the previous 24 hours with a parasite density > the parasite density of a episode of malaria diagnosed 2 days prior
   3. Fever (> 38.0°C tympanic) or history of fever in the previous 24 hours with a parasite density > 25% of the parasite density of a episode of malaria diagnosed 3 days prior
   4. Fever (> 38.0°C tympanic) or history of fever in the previous 24 hours with a positive thick blood smear of any parasite density occurring 4-14 days after the diagnosis of a previous episode

All patients diagnosed with new episodes of uncomplicated malaria will be prescribed artemether-lumefantrine (AL), the recommended first-line treatment in Uganda. Patients with complicated malaria or treatment failure following treatment with AL will be prescribed quinine according to national malaria treatment guidelines. Patients with treatment failure following treatment with quinine will be treated with quinine plus clindamycin.

On the day malaria is diagnosed, patients will also be prescribed paracetamol (10mg/kg) to take every 8 hours until resolution of fever. For patients with anemia (Hb < 10 gm/dL), we will follow the Integrated Management of Childhood Illness (IMCI) guidelines: anemic children will be prescribed iron sulfate (100 mg daily for 2 weeks) and mebendazole(only children > 1 year of age; 250 mg age 1-2 years; 500 mg > 2 years age; no more than every 6 months).

## Table 2 below provides a summary of the schedule for blood collection

| **Procedure** | **Baseline** | **Routine every 3 months** | **Routine every 6 months** | **Malaria diagnosed** |
| --- | --- | --- | --- | --- |
| Malaria smear & filter paper | X | X |  | X |
| Haemoglobin | X | X |  | X |
| Plasma from finger prick |  | X |  |  |
| Phlebotomy | X |  | X |  |

### 4.4.3 Non-malarial illnesses

Patients who are found to have illnesses other than malaria will receive standard-of-care treatment in the study clinic, according to local guidelines and algorithms, or will be referred for admission at the health care facility at the discretion of the attending clinic physician. If a patient is diagnosed with a non-malarial illness at the same time as malaria, treatment of the non-malarial illness will be at the discretion of the physician following the national treatment guidelines administered at the same time as the malaria treatment.

**4.4.4 Administration of medications**

All medications prescribed for study participants will be administered by the local health facility pharmacy and not by study personnel. The study will work with health facilities to ensure adequate supplies of antimalarial drugs and appropriate treatment for all diagnosed malaria episodes.

**4.5 Criteria for study discontinuation**

Study participants will be prematurely withdrawn from follow-up if they meet any of the following criteria: 1) permanent movement out of the sub-county, 2) inability to be located for > 4 months, 3) withdrawal of informed consent, or 4) inability to comply with the study schedule and procedures.

Participants who do not meet the above discontinuation criteria will be censured from the study when they reach their 11^th^ birthday or when the 2-year study follow-up period ends, whichever comes first.

## 4.6 Laboratory methods

### 4.6.1 Microscopy

Thick and thin blood smears will be stained with 2% Giemsa and read by experienced laboratory technologists who are not involved in direct patient care. Parasite densities will be calculated by counting the number of asexual parasites per 200 leukocytes (or per 500 leukocytes, if the count is <10 asexual parasites/200 leukocytes), assuming a leukocyte count of 8,000/μl. A blood smear will be considered negative when the examination of 100 high power fields does not reveal asexual parasites. Gametocytemia will also be determined from thick smears. Thin smears will be used for parasite species identification. Urgent thick smears will be read in our study clinic for initial diagnosis and to identify treatment failures during follow-up. Routine blood smears will be read within 48 hours. For quality control, all slides will be read by a second microscopist and a third reviewer will settle any discrepant readings.

### 4.6.2 Haemoglobin measurement

Hemoglobin will be measured from fingerprick blood samples using a portable spectrophotometer (HemoCue, Anglom, Sweden).

### 4.6.3 Molecular and Immunology Studies

Each time a thick blood smear is obtained; blood will also be collected onto filter paper. Samples will be collected by venipuncture or by finger prick sampling. Blood will be placed onto filter paper in approximately 25 μl aliquots per blood spot (4 blood spots per sample). The samples will be labelled with study numbers and dates, air-dried, and stored in small, sealed sample bags at ambient temperature or 4°C with desiccant. Molecular studies will include the extraction of DNA from filter paper and followed by characterization of parasite and host genetic polymorphisms using standard molecular procedures including PCR, DNA hybridization, and/or restriction enzyme digestion. For all repeat episodes of malaria, molecular genotyping methods, similar to those described for polymorphism analysis, will be used to distinguish recrudescent from new infections. Additional molecular studies will include analyses of polymorphisms in parasite genes for mutations that may impact on malaria infection and response to antimalarial therapy. Molecular studies will be performed only for research purposes and will have no impact on the clinical management of study patients.

Venipuncture and finger-prick plasma blood samples collected at baseline and at the time of routine assessments will be used in selected subjects for immunology. For venipuncture, approximately 5ml of blood will be collected and separated into plasma and peripheral blood mononuclear cells (PBMC) using a Ficoll gradient, following standard protocols.  Plasma will be stored at -80C for future immunologic studies, which may include measurement of levels of cytokines, antibodies, and other features related to the host immune response.  PBMCs will be stored at -80°C or in liquid nitrogen to maintain viability, and will be evaluated using flow cytometry, ELISPOT, and other assays to assess the host immune response.  Information from immunology studies will have no impact on patient care.

## 4.7 Household Survey

A Household Questionnaire (Appendix A) will be completed with the head of the household. This instrument is based on the model Malaria Indicator Survey questionnaires developed by the Roll Back Malaria Monitoring and Evaluation Reference Group, as well as other questionnaires from previous surveys conducted in Uganda, including the 2006 Uganda Demographic and Health Survey (UDHS) and the 2009 Malaria Indicator Survey.

The Household Questionnaire will obtain information on all residents in the selected households. Some basic information will be collected on the characteristics of each person listed, including age, sex, and relationship to the head of the household. The Household Questionnaire will also be used to collect responses on indicators of ownership and use of mosquito bed nets. The Household Questionnaire will also collect proxy indicators of wealth based on responses of household’s dwelling unit, ownership of various durable goods and land, and household food security.

# 5.0 DATA MANAGEMENT

## 5.1 Records to be kept

All clinical data will be recorded onto standardized case record forms (CRFs) by study personnel. Laboratory data will be recorded in a laboratory record book by the health center laboratory technologists and then transferred to the case record forms by study coordinators, who will review the case record forms daily for completeness and accuracy. Data will be entered directly from CRFs into a computerized database maintained at the study site clinics. All computerized data will be double entered to verify accuracy of entry and the database will be backed up on a daily basis and for quality control.

Query programs will be written into the database to limit the entry of incorrect data and ensure entry of data into required fields. All the entered data will later be transferred to the data centre in Kampala.

## 5.2 Data quality assurance and monitoring

All members of the study team will be educated in the study protocol prior to the onset of the study. Knowledge of the study protocol and procedures will be assessed and documented with a post-training questionnaire. The study clinicians will complete CRFs at each patient visit and also review these forms for completeness and accuracy. Study group meetings will be conducted regularly to review the progress of the study, address any difficulties, and provide performance feedback to the members of the study group.

## 5.3 Record keeping

CRFs will be provided for each subject. Participants will be identified by their study identification number on study documents and patient names will not be recorded on the CRFs or entered into the computerized database. All patient record forms will be kept in individual files in a secured filing cabinet in the study clinic. All corrections will be made on case record forms following GCP guidelines by striking through the incorrect entry with a single line and entering the correct information adjacent to it. All corrections will be initialled and dated by the study individual making the correction.

## 5.4 Statistical considerations and analysis

The primary statistical tests for this study will be descriptive in nature. Descriptive statistics will be stratified according to study site and age strata. A summary of the primary and secondary outcome measures are described in Table 3 below.

## Table 3. Outcome measures

| **Outcome** | **Definition** |
| --- | --- |
| Malaria incidence | Number of new episodes of malaria per time of observation |
| Prevalence of anemia | Proportion of study participants with anemia (mild = Hb < 10 gm/dL; moderate = Hb < 7.5 gm/dL; severe = Hb < 5.0 gm/dL) at the time of routine assessment |
| Parasite prevalence | Proportion of study participants with a positive thick blood smear at the time of routine assessment |
| Prevalence of malaria specific immune responses | Proportion of study participants with malaria specific immune responses at the time of routine assessment |

One of the primary purposes of this study will be to compare measures of malaria morbidity with measures from an ongoing health-facility based malaria surveillance program. In this study the primary measure of malaria morbidity will be the incidence of malaria in the cohorts described in this protocol. In our ongoing health-facility based malaria surveillance program, the primary measure of malaria morbidity is the slide positivity rate (SPR), defined as the proportion of patients with suspected malaria referred for diagnostic testing with a positive test result. It will not be possible to compare point estimates of malaria morbidity using our different methodologies due to differences in the units of measurement. Therefore, the primary metric for malaria morbidity will be the relative change in the incidence of malaria over time (r∆Im) defined as an incidence rate ratio = Im*_i+1_* / Im*_i_ ,* where Im*_i_* represents the incidence of malaria during the baseline time interval and Im*_i+1_* represents the incidence of malaria during interval immediately following using two month time intervals. For example if the Im*_i_* = 2 episodes per person year for Jan-Feb and Im*_i+1_* = 1 episode per person year for Mar-Apr, the r∆Im would equal 0.5 (e.g. 2-fold decrease in the incidence of malaria). For the gold standard method, r∆Im will be measured directly from our cohort studies stratified by age where malaria incidence will be equal to the number of cases of malaria per person per unit time. For the health-facility based method, r∆Im will be estimated using the SPR among patients stratified by age captured in our surveillance system by the following formula:

| Estimated r∆Im | = | Im *_i+1_* | = | SPR*_i+1_* (1 – SPR*_i_*) |
| --- | --- | --- | --- | --- |
|  |  | Im *_i_* |  | SPR*_i_* (1 – SPR*_i+1_*) |

We will test the hypothesis that estimates of r∆Im will be similar using the two methodologies. Point estimates of r∆Im from one 6 month time period to the next will be compared by calculating the variance of the streamlined method using the delta method and the variance of the gold standard method using a bootstrap test. Assuming that we will have a sample size of 1800 blood smears per 6 month period for SPR and 300 cohort participants per site, and assuming baseline SPR’s of 20%, 40%, and 70%, and baseline malaria incidence rates of 2, 4, and 6 cases per person year for low, medium, and high transmission areas respectively, we will have 80% power (one-sided significance level of 0.05) to reject the alternative hypothesis that there is an absolute difference in estimates of r∆Im of 0.3, 0.23 and 0.22 for low, medium, and high transmission sites, respectively. Whether or not these analyses show any evidence of significant differences between the methods, we will explore whether there is any systematic bias in the estimate of the streamlined method compared to the gold standard method (e.g. the streamlined method consistently over- or underestimates r∆Im). Ecological log-linear regression models will be used to predict the result of the gold standard method using data from the streamlined method, allowing adjustment for bias. Final models will then be prospectively validated in independent test sets to insure their accuracy.

In addition we will use statistical modelling to measure the impact of changing coverage levels of malaria control interventions on estimates of malaria incidence, parasite prevalence, anemia, and malaria specific immune responses in our cohorts. Detailed data will be collected prospectively on coverage levels of key malaria control interventions as detailed in Table 4 below. Data on IRS coverage will come from the Uganda MOH and implementing partners. Data on ITN coverage and ACT use will come from cross sectional surveys and health-facility based surveillance being collected separately from this study protocol.

Table 4. Malaria control intervention variables of interest

| **Category** | **Metric** | **Source of data** |
| --- | --- | --- |
| **IRS** | Date, formulation, and proportion of households sprayed | MOH records |
| ITNs | Percentage of household with at least one bednet | Cross-sectional surveys |
|  | Percentage of household with at least one ITN |  |
|  | Average number of nets per household |  |
|  | Average number of ITNs per household |  |
|  | Percentage of children age who slept under any net the prior night |  |
|  | Percentage of children age who slept under an ITN the prior night |  |
| ACTs | Proportion of febrile episodes in children treated with an ACT | Cross-sectional surveys |
|  | Proportion of antimalarial doses prescribed that were ACTs | Outpatient surveillance |
|  | Number of ACT doses prescribed at health care facility per month | Outpatient surveillance |

These analyses will be performed on a population rather than individual level, as data on malaria control interventions of interest are only available at a population level (population here defined as a particular sentinel site). To model the population effect of interventions for infectious processes, such as malaria, such population-level analyses are in fact preferable to individual-level analyses, as standard assumptions about independent outcomes between subjects are violated when disease dynamics are nonlinear. Indeed, population-level analyses may be more powerful as well as less biased in such situations.

To obtain appropriate causal inference on the effect of interventions on a population, we will use a counterfactual framework to estimate what the outcome variable of interest would be if the malaria control intervention were set to a specific level (e.g. what parasite prevalence would be if ITN coverage was set to 25%). In this framework, the counterfactual or missing data are defined as the unobserved levels of the intervention for a given population at a certain time. To evaluate the effect of an intervention (i.e. different levels of the counterfactual), we will fit a statistical model to appropriate covariates, then use the model to predict different outcomes at different levels of the intervention for a given population by changing the value of the covariate of interest (e.g. how much would the parasite prevalence change if ITN coverage increased from 25% to 50%).

The unit of analysis will be data from a single sentinel site during a 6-month period. An interval of 6 months was chosen as all intervention and exposure variables will be measured at least every 6 months. In addition, effects of interventions are expected to lag over the period of a few months or longer, so little information should be lost in aggregating variables collected more frequently than every 6 months. Analyses will be performed across all sentinel sites, allowing us to evaluate the potentially different effects of interventions when performed in areas of differing transmission intensity. However, estimates of the effect of interventions will only be evaluated for transmission intensities where these effects are identifiable (e.g. if no IRS is performed in medium transmission sites, we will not attempt to evaluate the effect of IRS in medium transmission sites even though our model could in theory compute an estimate).

Our dependent variables will be the outcome variables of interest (Table 3) for each site for each 6-month interval. Independent variables will include the outcome of interest from the prior interval (to assess change in the outcome), EIR and parasite rate from the prior time interval (to incorporate transmission intensity), and values of control intervention variables (Table 4) from appropriate prior intervals. Specifically, population level ITN use (proportion of households with at least one ITN) and population level ACT use (proportion of febrile episodes in children treated with an ACT) will be incorporated from only the prior interval, as any longer term effect of these interventions should be mediated through transmission intensity which is already parameterized. In contrast, IRS may have a longer term, cumulative effect and will be parameterized as two covariates: (number of sprayings in the last 10 years) x (proportion of households sprayed); (months since last spraying)^-1^ x (proportion of households sprayed).

Relationships between interventions and outcomes are likely to be non-linear and non-independent. Therefore, the use of standard linear models is unlikely to approximate true relationships, and pre-specified parametric models are likely to result in biased estimates. Numerous data-adaptive prediction algorithms (e.g. generalized boosted regression, multivariate adaptive polynomial spline regression, or random forests) exist to provide flexible estimates that may more closely reflect complex relationships found in nature, however it is difficult to predict a priori which of these algorithms will perform best with a particular type of data. Choosing the algorithm with the best prediction in a given data set (i.e. smallest residuals) is likely to result in over-fitting and produce a model which is not generalizable to other data sets. A data-adaptive approach that provides flexible estimates while avoiding over-fitting called Super Learner has recently been shown to possess strong theoretical advantages over other methods as well as providing excellent prediction in practice. In this approach, numerous pre-specified prediction algorithms, which may include simple least-squares regression models and more data-adaptive approaches, are fit on the data set. V-fold cross validation is used to select a convex combination of algorithms which minimizes a specified loss function (in this case L2-loss), then uses this combination of predictors fit on the entire data set to produce a final prediction for each data point. Super Learner prediction models will be fit to the outcome of interest using the appropriate model family for the dependent variable (e.g. Poisson for counts, binomial for proportions). The effect of an intervention on a given population will then be assessed by setting the counterfactual to desired levels and using the Super Learner to predict the outcome given the covariates for that population. Confidence intervals for each prediction will be calculated using a bootstrap procedure. Plots of outcomes versus levels of a given intervention will be produced to visualize the predicted effects of interventions for a given population. Specific parameters of interest (e.g. change in parasite prevalence for population “A” if ITN use goes from 25% to 50%) and confidence intervals associated with those parameters can be performed using the estimates and bootstrap-derived inference of the parameters.

# HUMAN SUBJECTS

**6.1 Risks and Discomforts**

**Privacy -** Care will be taken to protect the privacy of subjects and parents/guardians, as described in this protocol. However, there is a risk that others may inadvertently see patients’ medical information, and thus their privacy may be compromised.

**Finger Pricks and Venipuncture –** Routine sampling will be done by finger prick (~400μL or ~0.1 teaspoon), venous sampling will be done every 6 months (5mL or 1 teaspoon for children <5 years of age and 10mL or 2 teaspoons for older individuals). The total volume of blood taken per year will not exceed 12 mL for children <5 years of age or 21 mL for older participants. These volumes are too small to experience any side-effects from the blood drawing although a mild and transient feeling of discomfort/bruising may occur at the site of sampling.

Risks of these procedures include pain, transient bleeding and soft-tissue infection.

**6.2 Costs to the Subjects**

There will be no cost to the participant or their parents/guardians for participation in this study.

- 1. **Reimbursement of Subjects**

Participants will not be paid for their participation in the study. We will provide all routine medical care, including evaluations, medications available in the study clinics, and cost of any transportation free of charge.

**6.4 Informed consent**

Before enrolment into the study, parents/guardians will have to give informed consent for themselves and their children while children ≥ 8 years will be asked to provide assent. Only those who fulfil this criterion will be included into the study. The consent and assent forms to be used in the cohort study will be translated into the locally used languages used at the study sites and also back-translated into English to check for any loss or change of meaning. Japadhola and Swahili will be used in Nagongera, Lusoga in Walukuba while Rukiga and Runyakole will be used in Kihihi. The informed consent and assent discussion will be conducted by the study physician in an appropriate language that the participant’s parent/guardian understands and a translator will be used if necessary.

**6.5 Confidentiality**

All records will be kept as confidential as possible. Study participants will be identified only by a unique identification number. Patient names will not be entered into the computerized database. Study participant case record forms will be kept in individual files in a secured filing cabinet in the study clinics. Additional records will be kept in laboratory record books, which will be stored in the central study laboratory. No individual identities will be used in any reports or publications resulting from the study.

Much of the data to be collected for this study will come from tests and procedures normally done in the routine care of patients. All project staff will be trained on procedures for maintaining confidentiality and asked to sign a pledge of confidentiality.

## 6.6 Institutional review boards (IRBs)

This protocol, all procedures and consent forms, and any subsequent modifications must be reviewed and approved by the IRBs of all the participating institutions. This includes the following IRBs:

1. Uganda National Council of Science and Technology (UNCST)
2. Makerere University School of Medicine - Research and Ethical Committee (SOMREC)
3. University of California, San Francisco Committee for Human Research (UCSF-CHR)
4. London School of Hygiene and Tropical Medicine Ethics Committee

# REFERENCES

1. Breman JG, Alilio MS, Mills A. Conquering the intolerable burden of malaria: what’s new, what’s needed: a summary. Am J Trop Med Hyg. 2004; 71(suppl):1-15
2. World Health Organisation, World Malaria Report 2008
3. Lynch KI, Beach R, Asamoa K, Adeya G, Nambooze J and Janowsky E. President's Malaria Initiative, Rapid Assessment Report - Uganda, 2005
4. Okello PE, Van Bortel W, Byaruhanga AM, et al. Variation in malaria transmission intensity in seven sites throughout Uganda. Am J Trop Med Hyg 2006;75:219-25
5. Uganda Malaria Indicator Survey report, 2009
6. The Abuja Declaration and the Plan of Action, Abuja, 25^th^ April 2000
7. [O'Meara WP](http://www.ncbi.nlm.nih.gov/pubmed?term=%22O%27Meara%20WP%22%5BAuthor%5D), [Mangeni JN](http://www.ncbi.nlm.nih.gov/pubmed?term=%22Mangeni%20JN%22%5BAuthor%5D), [Steketee R](http://www.ncbi.nlm.nih.gov/pubmed?term=%22Steketee%20R%22%5BAuthor%5D), [Greenwood B](http://www.ncbi.nlm.nih.gov/pubmed?term=%22Greenwood%20B%22%5BAuthor%5D). Changes in the burden of malaria in sub-Saharan Africa. [Lancet Infect Dis.](javascript:AL_get(this,%20'jour',%20'Lancet%20Infect%20Dis.');) 2010 Aug;10(8):545-55.
8. World Health Organisation. World malaria report 2009
9. Breman JG, Holloway CN. Malaria surveillance counts. Am J Trop Med Hyg 2007;77:36-47
10. Cibulskis RE, Bell D, Christophel EM, et al. Estimating trends in the burden of malaria at country level. Am J Trop Med Hyg 2007;77:133-7
11. Chilundo B, Sundby J and Aanestad M. Analysing the quality of routine malaria data in Mozambique. Malar J 2004;3:3
12. Uganda National guidelines for research involving humans as research participants, March 2007
13. WHO. Guidelines for the treatment of malaria, 2nd edition. 2010.

# Appendix A. HOUSEHOLD SURVEY

The household survey will be administered through a completely paperless QDS software system. However the data dictionary of the questionnaire which provides information on the questions being asked and coding system used is attached.

# Appendix B. WHO CRITERIA FOR SEVERE MALARIA/DANGER SIGNS

Criteria for severe malaria

- Cerebral malaria - defined as unarousable coma not attributable to any other cause in a patient with falciparum malaria
- Generalized convulsions (> 3 convulsions over 24 hours period)
- Severe normocytic anemia (Hb < 5 gm/dL)
- Hypoglycemia
- Metabolic acidosis with respiratory distress
- Fluid and electrolyte disturbances
- Acute renal failure
- Acute pulmonary edema and adult respiratory distress syndrome (ARDS)
- Circulatory collapse, shock, septicemia ("algid malaria")
- Abnormal bleeding
- Jaundice

Danger signs (for children < 5 years of age)

- Less than 3 convulsions over 24 hour period
- Inability to sit up or stand
- Vomiting everything
- Unable to breastfeed or drink
- Lethargy

# Appendix C. Schedule of evaluations for study participants

| **Evaluations** | **Baseline (enrolment)** | **Approx. every 3 months** | **Approx. every 6 months** | **New visits for medical problems** | **Day malaria diagnosed** |
| --- | --- | --- | --- | --- | --- |
| Informed consent | X |  |  |  |  |
| History and physical | X | X |  | X | X |
| Hemoglobin measurement | X | X |  |  | X |
| Malaria smear & filter paper | X | X |  | X* | X |
| Plasma from finger prick |  | X |  |  |  |
| Phlebotomy for immunology studies | X |  | X |  |  |
| ITN distribution | X |  |  |  |  |

* if fever present
