# Supplementary material for: Measures of Malaria Burden after Long-Lasting Insecticidal Net Distribution and Indoor Residual Spraying at Three Sites in Uganda: A Prospective Observational Study
Source: PLoS Med. 2016 Nov 8;13(11):e1002167. doi: 10.1371/journal.pmed.1002167 (PMC5100985; doi:10.1371/journal.pmed.1002167)
Supplement: S3 Text — (DOCX) [file pmed.1002167.s008.docx]

**“Program for resistance, immunology, surviellance, and modelling of malaria” (PRISM)**

**Study Title:** **Entomological surveillance for estimating malaria transmission intensity in 3 sites in Uganda**

**DMID Protocol Number:** 10-0064

**Sponsored by:** National Institute of Allergy and Infectious Diseases (NIAID)

**DMID Funding Mechanism:** NIH/NIAID U19AI089674

**Principal Investigator:** Moses R. Kamya, MBChB, MPH, PhD

**DMID Protocol Champion:** Malla Rao, DrPH, M Engg

**Draft Number:** 1.0, dated: 24 January 2011

Table of Contents

[PROJECT SUMMARY 4](#_Toc281394802)

[INVESTIGATORS 5](#_Toc281394803)

[OTHER KEY STUDY PERSONNEL 6](#_Toc281394804)

[ABBREVIATIONS AND ACRONYMS 7](#_Toc281394805)

[1.0 BACKGROUND 8](#_Toc281394806)

[1.1 Introduction 8](#_Toc281394807)

[1.2 Human landing catches 8](#_Toc281394808)

[1.3 Light traps 9](#_Toc281394809)

[1.4 Pyrethrum spray and exit trap collections 9](#_Toc281394810)

[1.5 Vector biting behavior 9](#_Toc281394811)

[1.6 Basic reproductive number 10](#_Toc281394812)

[1.7 Larval breeding sites 10](#_Toc281394813)

[1.8 Insecticide resistance 10](#_Toc281394814)

[2.0 RATIONALE 11](#_Toc281394815)

[3.0 OBJECTIVES 11](#_Toc281394816)

[4.0 RESEARCH DESIGN 12](#_Toc281394817)

[4.1 Study sites 12](#_Toc281394818)

[Table 1. Characteristics of selected districts 12](#_Toc281394819)

[4.2 Sampling frame 13](#_Toc281394820)

[4.3 Outcome measures 14](#_Toc281394821)

[5.0 STUDY PROCEDURES 14](#_Toc281394822)

[5.1 Human landing catches 14](#_Toc281394823)

[Table 2. Schedule for human landing catches, where letters donate individual field workers 15](#_Toc281394824)

[5.2 Light trap collections 16](#_Toc281394825)

[5.3 Pyrethrum spray and exit trap collections 17](#_Toc281394826)

[5.4 Environmental measurements 18](#_Toc281394827)

[5.5 Larval surveys 18](#_Toc281394828)

[6.0 LABORATORY METHODS 18](#_Toc281394829)

[6.1 Processing of mosquito specimens 18](#_Toc281394830)

[6.2 Estimation of entomologic indicators 19](#_Toc281394831)

[Table 3. Entomological indicators measured by each collection method 19](#_Toc281394832)

[7.0 ANALYTICAL PLAN 20](#_Toc281394833)

[Table 4. Power calculations for comparing estimates of EIR 20](#_Toc281394834)

[8.0 DATA MANAGEMENT 21](#_Toc281394835)

[9.0 PROTECTION OF HUMAN SUBJECTS 21](#_Toc281394836)

[9.1 Institutional review boards 22](#_Toc281394837)

[9.2 Informed consent process 22](#_Toc281394838)

[9.3 Risks and discomforts 23](#_Toc281394839)

[9.4 Benefits 24](#_Toc281394840)

[9.5 Confidentiality 24](#_Toc281394841)

[9.6 Compensation 24](#_Toc281394842)

[10.0 REFERENCES 25](#_Toc281394843)

[Appendix 1. Sample timetable of weekly activities 27](#_Toc281394844)

# PROJECT SUMMARY

| **Title** | Entomological surveillance for estimating malaria transmission intensity in 3 sites in Uganda |
| --- | --- |
| **Project Sites** | 1. **Tororo:** Nagongera sub-county, a rural area in Tororo district in eastern Uganda, is an area of presumed high malaria transmission intensity 2. **Jinja:** Walukuba sub-county, a peri-urban area in Jinja district in the central region, is an area of presumed medium malaria transmission intensity 3. **Kanungu:** Kihihi sub-county, a rural area in Kanungu district in western Uganda, is an area of presumed low malaria transmission intensity |
| **Duration** | 30 months |
| **Activities** | 1. **Human-landing catches:** These will be conducted indoors and outdoors in and around eight randomly selected houses at each site each month. 2. **Light trap collections:** At each of the 3 sentinel sites, CDC light trap collections will be placed in 100 houses which have been randomly selected for participation in the PRISM cohort study every two weeks. 3. **Pyrethrum spray + exit trap collections:** Ten randomly selected houses will be sprayed with an aerosol of non-residual pyrethroids with a piperonyl butoxide synergist each month in each site. Window trap collections will also be placed one night every 4 weeks. 4. **Environmental data:** Temperature data loggers will be positioned indoors and outdoors in 6 houses representative of typical homes in each site and records made for the entire study period. |
| **Objectives** | 1. To estimate the malaria transmission intensity, as measured by the entomological inoculation rate (EIR), in the 3 study areas. 2. To compare the relative ability of human landing catches, CDC light trap collections and pyrethrum spray collections and exit traps combined to measure human biting rates. 3. To determine the proportion of bites by malaria vectors which occur outdoors relative to those received indoors. 4. To determine the Basic Reproductive Number (R_0_) in the study sites by measuring the human-landing rate, proportion of blood-fed mosquitoes that have fed on people (human biting index) and mosquito population survival (parity rate). 5. To determine the distribution of larval breeding sites during the study. 6. To collect mosquitoes for determination the susceptibility of malaria vectors to insecticides commonly used for malaria control. |

# INVESTIGATORS

**Moses Kamya, MBChB, MPH, PhD**

Role in project: Principal Investigator

Professor, Department of Medicine, Makerere University, Kampala, Uganda

Director, Infectious Disease Research Collaboration / Uganda Malaria Surveillance

Project, Kampala, Uganda

Email: [mkamya@infocom.co.ug](mailto:mkamya@infocom.co.ug)

**Steve Lindsay, PhD**

Role in project: Co-Principal Investigator

Professor of Public Health Entomology, Department of Disease Control

London School of Hygiene and Tropical Medicine, London, UK

Email: [steve.lindsay@lshtm.ac.uk](mailto:steve.lindsay@lshtm.ac.uk)

**Grant Dorsey, MD, PhD**

Role in project: Co-investigator

Associate Professor, Department of Medicine, University of California, San Francisco

Email: [gdorsey@medsfgh.ucsf.edu](mailto:gdorsey@medsfgh.ucsf.edu)

**Sarah Staedke, MD, PhD**

Role in project: Co-investigator

Clinical Senior Lecturer, Department of Clinical Research

London School of Hygiene and Tropical Medicine, London, UK

Email: [sarah.staedke@lshtm.ac.uk](mailto:sarah.staedke@lshtm.ac.uk)

**Martin Donnelly, MA, MSc, PhD**

Role in project: Co-investigator

Reader in Vector Biology, Liverpool School of Tropical Medicine, Pembroke Place, Liverpool, UK

Email: mjames@liv.ac.uk

**Kilama Maxwell, BA**

Role in the project: Co-investigator

Entomologist, Uganda Malaria Surveillance Project, Kampala, Uganda

Email: [kilamam@gmail.com](mailto:kilamam@gmail.com)

**Anne Gasasira, MBChB, MSc, PhD**

Role in the project: Collaborator

Epidemiologist, Uganda Malaria Surveillance Project, Kampala, Uganda

Email: agasasira@gmail.com

# OTHER KEY STUDY PERSONNEL

| **Name** | **Title** | **Affiliation** |
| --- | --- | --- |
| Geoff Lavoy | Data manager | Uganda Malaria Surveillance Project, Kampala |
| Ruth Kigozi | Data manager | Uganda Malaria Surveillance Project, Kampala |

# ABBREVIATIONS AND ACRONYMS

| ACTs | artemisinin combination therapies |
| --- | --- |
| CDC | Centers for Disease Control |
| EIR | Entomological Inoculation Rate |
| ELISA | Enzyme-linked immunosorbent assay |
| ETs | Exit traps |
| GIS | Geographical Information System |
| HLC | Human-landing collections |
| IRS | indoor-residual spraying |
| ITNs | insecticide-treated nets |
| IVM | Integrated Vector Management |
| LLINs | long-lasting insecticide treated nets |
| LTs | CDC light traps |
| MOH | Ministry of Health |
| NIH | National Institutes of Health, USA |
| PCR | Polymerase chain reaction |
| PI | Principal Investigator |
| PSC | Pyrethrum spray catches |
| RFLP | Restriction fragment length polymorphism |
| Sp | Sporozoite |
| WHO | World Health Organisation |

# 1.0 BACKGROUND

## 1.1 Introduction

Despite recent evidence that the burden of malaria is decreasing in many endemic areas [1], malaria remains one of the most serious global health problems and is the leading cause of morbidity and mortality in Uganda. The malaria-specific mortality is estimated to be between 70,000 and 100,000 child deaths annually in Uganda, a death toll that far exceeds that of HIV/AIDS (Uganda MOH). According to the 2008 World Malaria Report, Uganda ranked 6^th^ in terms of number of malaria cases and 3^rd^ in terms of number of malaria deaths. Previous work in Uganda has documented some of the highest transmission intensities reported in the world, [2] and demonstrated substantial variability in malaria transmission in different parts of the country. However, these studies were conducted 10 years ago, and little evidence is currently available on the level of malaria transmission in Uganda.

There is a substantial body of evidence supporting the widespread use of indoor-residual spraying (IRS), insecticide-treated nets (ITNs), and artemisinin combination therapies (ACTs) in sub-Saharan Africa [3, 4]. However, considerable gaps in knowledge remain regarding the impact of these interventions in operational settings under different epidemiological conditions, in various sequences and combinations, and over an extended period of time. The World Health Organization is advocating Integrated Vector Management (IVM) [5], where a combination of tools are used to reduce malaria, yet there is little evidence to support which strategies work best in different eco-epidemiological settings. This study proposed here will provide important insights into which combinations of control measures are effective at reducing malaria over several years. Understanding the current level of malaria transmission in Uganda, variations across the country, and changes over time is critical to evaluating the impact of control interventions.

## 1.2 Human landing catches

Classically, the intensity of malaria transmission is estimated by human-landing catches, where mosquitoes are caught landing on the exposed limbs of field workers. Human landing catches are widely regarded as the ‘gold standard’ for estimating transmission intensity, and were used in the prior studies conducted by Okello and colleagues to estimate EIR [2]. However, this method is not ideal as field workers may be potentially exposed to malaria during the collection process. To prevent this, the WHO [6] recommends that field workers *“…should take an appropriate and effective antimalarial prophylaxis to avoid contracting malaria during collection of biting mosquitoes. Furthermore, it is not necessary to permit mosquitoes to feed; they should be collected as soon as they settle on the skin, since it can be safely assumed that biting would normally follow. Landing rates should therefore be measured instead of biting rates*’

## 1.3 Light traps

CDC light traps are positioned indoors next to a person sleeping under a treated bednet. The trap collects mosquitoes trying to feed on people and reduces the number of bites a person would receive from vectors. Moreover all individuals in the room are protected from mosquitoes because they are sleeping under nets. This is a standardised method of sampling where the catch is not dependent on the operator. Most people like having a small light in the house at night so there are rarely problems with compliance.

## 1.4 Pyrethrum spray and exit trap collections

Spraying insecticides indoors early in the morning is an activity normally appreciated by householders since it reduces the number of mosquitoes in the house. Exit traps in windows are not a problem, although if fitted to doorways there may be issues concerning safety as the occupants cannot lock their door at night. In the study areas there is not a problem with security so we do not anticipate problems. At cold times of the year some people complain that exit traps maker the room cold at night. If this occurs, we will provide house occupants with blankets.

## 1.5 Vector biting behavior

Understanding vector biting behaviour, the location of biting, and the proportion of mosquitoes that bite outdoors, is critically important. Whilst typically around 70-80% of biting by vectors occurs indoors [7, 8] there is growing evidence that as transmission falls the proportion of biting by vectors outdoors increases [9]. This probably arises since our main vector control tools of LLINs and IRS is directed largely against vectors entering houses. Since these are killed and/or repelled by the insecticide it may be that selection for outdoor biting occurs. This is potentially important since our ability to protect ourselves against outdoor biting is poor. A further difficulty is that our ability to measure outdoor biting, other than by using human-landing catches is weak.

## 1.6 Basic reproductive number

One of the most powerful measures used to describe the nature of malaria transmission is based on the Basic Reproductive Number (R_0_) [10, 11]. This is defined as the number of people that can be infected that arises from a single person infected with malaria parasites, assuming that all individuals are susceptible to infection. Where R_0_ is >1 transmission can be maintained and where it <1 the disease will be eliminated. To estimate R_0_ we need to know the biting rates, the frequency of vectors feeding on people, vector survival and a few other variables one can parameterise from sources in the literature. For this reason we will determine the proportion of blood feeds taken by vectors which are on people and the survival of the mosquito population which is based on the proportion of older mosquitoes in the population i.e. those that have laid eggs.

More recently the concept of R_0_ has been extended to capture the heterogeneity in malaria transmission in a population. This includes the variation in the seasonal and spatial nature of transmission, but also the variation in breeding sites in space and time.

## 1.7 Larval breeding sites

Assessing the distribution of breeding sites is important since generally aquatic habitats close to human habitation are more likely to be more important for malaria transmission than those further away. If breeding sites are readily located, accessible and not too numerous larval source management may prove to be an additional method for vector control in the study sites.

## 1.8 Insecticide resistance

Four main classes of insecticides are available to control anopheline mosquitoes, the vectors of malaria, including organochlorine (DDT), pyrethroids, carbamates, and organophosphates[12]. The four insecticidal classes are directed against only two targets, and there is considerable cross resistance. Resistance to insecticides, in particular pyrethroids and DDT, puts vector control strategies at risk. Insecticide resistance in the primary African malaria vectors *Anopheles gambiae*, *A. arabiensis* and *A. funestsus*, has been seen against all classes of insecticides available to control anopheline mosquitoes [13-16]. Most alarming is resistance to pyrethroids, as this is the only class currently utilized for ITNs and is also a key component of many IRS programs [17]. Resistance to pyrethroids is already hampering malaria control programs [18, 19]. Evaluating markers of insecticide resistance will be an important component of longitudinal surveillance in Uganda, however, our understanding of mechanisms of resistance is currently incomplete.

# 2.0 RATIONALE

In this study, we propose to estimate malaria transmission intensity, as measured by EIR, using three different methodologies including human landing catches (the gold standard), CDC light traps, and pyrethrum spray catches combined with exit trap collections. The study will be conducted in three different study sites, all of which were included in the original survey conducted Okello and colleagues in Uganda (Tororo, Jinja, and Kanungu ) [2]. The proposed study will provide important information about the degree of malaria transmission in Uganda, and whether it is as intense today. In addition, we aim to compare the alternative collection methods to the gold standard in the anticipation that one of these techniques can be used to estimate transmission effectively, allowing us to confine human-landing catches to the history books.

The information collected in this study will complement and support other studies which will describe the clinical pattern of infection and morbidity, as well as the level of antimalarial drug resistance in the parasite populations and insecticide resistance in local vector populations, in the same sites. The collection and integration of these diverse data sets will provide a robust characterisation of malaria in the study sites and establish an essential framework for developing future interventions against this disease.

This work will capture the intensity and variability of malaria transmission in the study sites and the distribution and nature of larval breeding sites. This information will also provide specimens for determining the level and nature of the susceptibility of mosquitoes to insecticides. In addition, it will also allow the team to develop mathematical models which describe the nature of malaria transmission in the study area and can be used for determining the most effective way in which to combine interventions against malaria in the future.

# OBJECTIVES

1. To estimate the malaria transmission intensity, as measured by the EIR, in the 3 study areas
2. To compare the relative ability of human landing catches, CDC light trap collections and pyrethrum spray collections and exit traps combined to measure human biting rates
3. To determine the proportion of bites by malaria vectors which occur outdoors relative to those received indoors
4. To determine R_0_ in the study sites by measuring the human-landing rate, proportion of blood-fed mosquitoes that have fed on people (human biting index) and mosquito population survival (parity rate)
5. To determine the distribution of larval breeding sites during the study
6. To collect mosquitoes for determination the susceptibility of malaria vectors to insecticides commonly used for malaria control

# 4.0 RESEARCH DESIGN

## 4.1 Study sites

We propose to carry out detailed entomological surveillance in (1) Nagongera sub-county in Tororo district, (2) Walukuba sub-county in Jinja district, and (3) Kihihi sub county in Kanungu district for 30 months starting in April 2011 (Table 1).

### Table 1. Characteristics of selected districts

| **Characteristics** | **District** | | |
| --- | --- | --- | --- |
|  | **Tororo** | **Jinja** | **Kanungu** |
| **Demographics** ^a^ | | | |
| Population | 536,900 | 387,600 | 204,700 |
| Urbanization level | 6.5% | 22.1% | 6.3% |
| Number of households | 112,300 | 84,000 | 43,500 |
| **Entomology (data collected 2001-2002)** | | | |
| Entomologic inoculation rate | 562 | 6 | 6 |
| Predominate vector species | *An.*  *gambiae ss* | *An.*  *gambiae ss* | *An.*  *gambiae ss* |
| **Measures of infection (data collected in children 2-9 years of age, 1999)** | | | |
| Parasite rate | 91% | 15% | 43% |

^a^ Uganda Population and Housing Census, 2002 (Ugandan Bureau of Statistics)

Nagongera sub-county is located in Tororo district, eastern region. The area is a rural setting characterized by dry savannah grassland interrupted by bare rocky outcrops and low lying swamps, although natural vegetation has mostly been replaced by cultivated crops. Average daytime temperature is 27°C, with two rainy seasons (March to May and August to October); annual rainfall is 1,000-1,500 mm^2^. At an elevation of 1,185m above sea level the sub-county represents an area of presumed high malaria transmission with annual entomological inoculation rates of 562 infective bites/year [2]. The major malaria vector species in the region are Anopheles gambiae s.s., An. funestus and small numbers of *An. arabiensis* [2, 20].

Walukuba sub-county is a peri-urban area in Jinja district, central region. The population of Jinja was estimated at 387,600 based on the 2002 national census. It is a swampy area near Lake Victoria. At an elevation of 1215m above sea level, it is an area of presumed medium malaria transmission with an EIR of 6 infective bites/year [2]. The major malaria vector species in the region is An. gambiae s.s. [2].

Kihihi sub-county is a rural sub-county in Kanungu district in western Uganda with presumably relatively low malaria transmission intensity at 6 infective bites/year [2]. Located at an elevation of 1310m, it is a mountainous rural setting.

## 4.2 Sampling frame

Prior to the onset of this study, all households in each sub-county in the 3 sites will be enumerated and mapped, which will be used to generate a sampling frame for the entomology surveys. The enumeration of households is being conducted as part of a separate approved protocol. All households enumerated during the survey will be assigned a unique number. A random sample of households will be selected from each site to generate a list of households to be approached for recruitment into the entomology surveys. From these lists, households will be selected for participation in the human landing catches, pyrethrum spray + exit trap collections, and environmental measures (Figure). A separate list of random households will be selected from each site to generate a list of households to be approached for recruitment into the cohort studies being conducted under a separate protocol. The households of all children recruited into the cohort study will be approached for the CDC light trap collections.

**Figure.** Sampling frame for random selection of household for entomology surveys

Enumeration list of all households from each sub-county

Random list of households to be selected from for cohort studies

Random list of households to be selected from for only entomology surveys

Selection of 6 households for environmental measurements

Selection of 10 households for spray and exit trap collections

Selection of 100 households for light trap collections

Selection of 8 households for human landing catches

##

## 4.3 Outcome measures

The primary outcome measures for each study site are:

- annual number of bites/person/year (EIR) by malaria vectors from human-landing catches

The secondary outcome measures for each study site are:

- annual number of bites/person/year (EIR) by malaria vectors from light trap catches
- annual number of bites/person/year (EIR) by malaria vectors from pyrethrum spray catches & exit traps
- Basic reproductive number (R_0_)
- Describe the seasonal variation in vector biting rates
- Identify the principal breeding sites of anophelines
- Assess the susceptibility of vectors to major classes of insecticide

# 5.0 STUDY PROCEDURES

## 5.1 Human landing catches

**5.1.1 Overview**

Human-landing catches will be conducted indoors and outdoors in and around randomly selected houses at each site each month. The catches are designed to replicate normal human subject behaviour, assuming many residents will be outdoors in the early evening, and that most will retire to bed at 22.00h. This is done not only to ensure representativeness of collection but also to ensure that the risk of infection from mosquito-borne diseases is not increased above normal.

**5.1.2 Study population**

In each site, 8 households will be randomly selected for the catches from the entomology recruitment list generated from the enumeration database in each site. The same 8 households will be sampled each month. Written informed consent from the head of household or an adult household representative will be obtained prior to conducting the landing catches. At each site, two different houses will be used each night, at least 300m apart, for four consecutive nights. Thus, all 8 households will be sampled in the same week each month.

**5.1.3 Recruitment of field workers**

At each site, a team of 8 field workers will be recruited for the human landing catches. The criteria for recruitment will include: (1) Willingness to participate in indoor and outdoor landing catches from 17.00 to 07.00 hours the following morning, (2) Willingness to take chemoprophylaxis for malaria, (3) Willingness to abstain from alcohol during working hours, and (4) No significant past medical history. Field workers will be recruited and screened for the eligibility criteria by UMSP supervisors. The same field workers used for the human landing catches will be used for all components of the entomology survey as outlined in Appendix 1. Field workers will be paid for their work and provided malaria chemoprophylaxis, including mefloquine (250mg tab orally once weekly) or doxycycline (100mg tab orally each day). The risks and benefits of each treatment option will be explained to the field workers, and they will be allowed to choose their preferred treatment. Field workers will also be offered medical treatment for any illness that develops during the period of their employment.

**5.1.4 Methods**

At each house two field workers will be stationed outdoors 10m from the house, and two will be stationed indoors. Outdoor collections will be conducted from 18.00h to 21.50h. Indoor collections will be conducted from 18.00h to 05.50h. Field workers will collect mosquitoes landing on their exposed legs using aspirators and torchlight. Field workers will collect mosquitoes for 50 minutes, with a 10 minute break each hour. They will be rotated between sites using the schedule shown in Table 2.

**Table 2. Schedule for human landing catches, where letters donate individual field workers**

| **Time** | **House 1** | | **House 2** | |
| --- | --- | --- | --- | --- |
|  | **Outdoors** | **Indoors** | **Outdoors** | **Indoors** |
| **Day 1** | | | | |
| 18.00-21.50h | A, B | C, D | E, F | G, H |
| 22.00-02.50h |  | A, B |  | E, F |
| 03.00-05.50h |  | C, D |  | G, H |
| **Day 2** | | | | |
| 18.00-21.50h | C, D | A, B | G, H | E, F |
| 22.00-02.50h |  | C, D |  | G, H |
| 03.00-05.50h |  | A, B |  | E, F |
| **Day 3** | | | | |
| 18.00-21.50h | A, B | C, D | E, F | G, H |
| 22.00-02.50h |  | A, B |  | E, F |
| 03.00-05.50h |  | C, D |  | G, H |
| **Day 4** | | | | |
| 18.00-21.50h | C, D | A, B | G, H | E, F |
| 22.00-02.50h |  | C, D |  | G, H |
| 03.00-05.50h |  | A, B |  | E, F |

This will require 4 field workers/house, or a total of 8 field workers per night, plus one supervisor, at each site. Mosquitoes will be collected at hourly intervals through the night and processed the following morning. The number of mosquitoes collected outdoors by human landing catches from 18.00 to 21.50h will be compared with those collected indoors from 22.00 to 5.50h.

## 5.2 Light trap collections

**5.2.1 Overview**

The human landing collections will be supplemented by extensive collections using CDC light traps. The CDC light traps will be used to collect mosquitoes from households involved in a parallel cohort study recording the incidence of malaria in children, which is being conducted under a separate study protocol.

**5.2.2 Study population**

From the enumeration database, a separate list of randomly selected households will be generated for possible recruitment into the cohort study (Figure). All children aged 6 months to 10 years, who meet the eligibility criteria, will be recruited from 100 households at each site, and followed for 2 years. Study participants will receive all routine medical care at a study clinic located within the UMSP government run level IV health center. Routine medical care will be provided free of charge and transport costs to and from the clinic will be reimbursed. Written informed consent from the head of household or an adult household representative will be obtained prior to conducting the CDC light trap collections. Households participating in the cohort studies will have the option of not participating in the CDC light trap collections.

**5.2.3 Methods**

Mosquitoes will be sampled using miniature CDC light traps (Model 512; John W. Hock Company, Gainesville, Florida, USA) positioned 1m above the floor at the foot end of the bed where a person sleeps under an ITN. Traps will be set at 19.00h and collected at 07.00h the following morning by field workers. If the trap cannot be set-up in the intended house, the trap will be moved to the nearest similar house after obtaining written informed consent from the head of household or an adult household representative. If the occupant does not spend the night in the selected room or the trap is faulty, the data will be excluded from the analysis. The number of children and adults who slept in the house the previous night will be determined and the presence of long-lasting insecticide treated nets (LLINs) recorded. Each night approximately 12 traps will be set for 4 nights in each week. The 100 cohort study houses will be sampled every other week during the study. Sampling schedules are shown in Appendix 1.

## 5.3 Pyrethrum spray and exit trap collections

**5.3.1 Overview**

Randomly selected houses will be sprayed using an aerosol of non-residual pyrethroids with a piperonyl butoxide synergist each month. These sprays will be combined with exit traps placed over the windows of the houses to capture any escaping mosquitoes.

**5.3.2 Study population**

In each site, 10 households will be randomly selected for the spray collections from the entomology recruitment list generated from the enumeration database in each site. The same 10 households will be sampled one day every 4 weeks. Written informed consent from the head of household or an adult household representative will be obtained prior to conducting the pyrethrum spray and exit trap collections. Sampling schedules are shown in Appendix 1.

**5.3.3 Methods**

Collection will take place between 06.00-08.00h. The number of children and adults who slept in the house the previous night will be determined and the presence of long-lasting insecticide treated nets (LLINs) recorded. White sheets will be spread on the floor and over the furniture in the house. Two field workers, one inside the house and one outside, will spray around the eaves with 0.025% pyrethrum emulsifiable concentrate with 0.1% PBO in kerosene. The field worker inside the house will then spray the roof and walls. The house will be closed for 10 minutes after which the white sheets will be brought outside (where there is sufficient light), and dead mosquitoes will be collected from the sheets and transferred to the field laboratory on moist filter papers in petri dishes for identification and processing.

To collect house-leaving mosquitoes, window exit traps will be set at 18.00h and collected between 06-07.00h the following morning. Mosquitoes from each trap will be put into paper cups separately and transferred to the field laboratory for processing. Mosquitoes will be provided with sugar solution for 12 hours from the time of collection. Parity dissections will be performed on 500 of each species each month at each site.

## 5.4 Environmental measurements

**5.4.1 Overview**

Temperature is a critical measurement since it governs the rate at which sporozoites develop in vectors. Here we propose to record changes in indoor and outdoor temperatures during the study.

**5.4.2 Study population**

In each site, 6 households will be randomly selected for the environmental measurements from the entomology recruitment list generated from the enumeration database in each site. Written informed consent from the head of household or an adult household representative will be obtained prior to conducting the environmental measurements. The houses will be representative of typical homes in each site, and the same 6 households will be sampled for the duration of the study.

**5.4.3 Methods**

Temperature data loggers will be positioned indoors and outdoors in the selected households. Tiny tag data loggers will be positioned on the wall indoors and outdoors and will record information for 24h each month.

## 5.5 Larval surveys

Each study site will be surveyed for water bodies each month. Site coordinates will be recorded using a GPS. Purposeful sampling will be done to maximize collection of the aquatic stages of mosquitoes using a 350-mL dipper (Clarke Mosquito Control Products, Roselle, IL). At each site, 10 dips will be made in places likely to harbour mosquito larvae, such as around tufts of submerged vegetation or substrate, edges of water bodies, and around floating debris. In extensive water bodies, dipping will be carried out over a 100-m walk. Larvae will be classified either as anophelines or culicines. Anopheline larvae will be stored in 100% ethanol, which will be refreshed on reaching the laboratory. Randomly selected subsamples of anopheline larvae selected during the routine mapping of the area sand sibling species of the *An. gambiae* complex will be identified by amplification of ribosomal DNA using polymerase chain reaction (PCR) [21].

# 6.0 LABORATORY METHODS

## 6.1 Processing of mosquito specimens

A large number of specimens will be collected from the different sites and from the different collection methods. All anophelines and culicines will be identified taxonomically to species level where possible. To process the mosquitoes, we will implement a systematic procedure for labelling and recording the specimens which will include the following information: 1) area where the samples were collected, 2) house number (which will be linked to GIS data), 3) method of collection, 4) date of collection, and 5) serial number of the specimen. When processing the specimens, labels will be written in pencil and placed with the relevant specimens in eppendorf tubes and similar information recorded in a register for easy data entry and cross-checking.

## 6.2 Estimation of entomologic indicators

A list of entomological indicators that will be measured and the methods used to generate this data are summarized in Table 3 below.

### Table 3. Entomological indicators measured by each collection method

| **Indicator** | **Extended surveys** | | | **CDC light trap collection** |
| --- | --- | --- | --- | --- |
|  | **Man-landing collections** | **Pyrethrum spray collection** | **Window trap collection** |  |
| Species | X | X | X | X |
| Vector density | X |  | X |  |
| Biting rate | X | X |  | X |
| Physiological status | X | X | X | X |
| Parity rate | X | X | X | X |
| Sporozoite infection rate | X | X |  | X |
| Human blood index | X | X |  |  |
| Proportion resting indoors |  | X | X |  |
| Proportion exiting after blood meal |  | X | X |  |
| Proportion biting early or late | X |  |  |  |

**6.2.1 Species determination**

Identification of anophelines will be based on morphological criteria according to established taxonomic keys [22, 23]. Identification of members of the *An. gambiae* complex will be by PCR [21].

**6.2.2 Sporozoite infection rate**

Sporozoites will be identified in individual mosquitoes stored on desiccant using an ELISA technique [24].

**6.2.3 Human blood index**

The source of mosquito blood meals will be assessed on abdomens squashed on filter paper and identified using an ELISA technique [25].

#

# 7.0 ANALYTICAL PLAN

We will test the hypothesis that annual estimates of EIR will be similar using the human landing catches and the alternative methodologies (CDC light traps and pyrethrum spray + exit trap collections). Point estimates of EIR using the different methods will be calculated at monthly intervals and compared annually using the non-parametric Wilcoxon matched-pairs signed-ranks test. Sample sizes will be determined by the human biting rate, number of mosquitoes tested for sporozoites, and the sporozoite rate. Power calculations based on published estimates of the human biting rate and sporozoite rate in high and low transmission intensity settings [4] are presented in Table 4. In our high transmission setting, we will have 80% power (one-sided significance level of 0.05) to reject the alternative hypothesis that there is a difference between our estimates assuming that the annual EIR using the gold standard method is 1,586 and the annual EIR using the streamlined methods is within the range of 1,205-1,964. In our low transmission setting, we will have 80% power (one-sided significance level of 0.05) to reject the alternative hypothesis that there is a difference between our estimates assuming that the annual EIR using the gold standard method is 4 and the annual EIR using the streamlined methods is within the range of 0–24.

### Table 4. Power calculations for comparing estimates of EIR

| Level of transmission intensity | Gold standard method estimates | | | Alternative method |
| --- | --- | --- | --- | --- |
|  | Average nightly human biting rate | Average sporozoite rate | Annual EIR | Range of annual EIRs for which no significant difference detected |
| High | 108 | 0.0402 | 1586 | 1205 - 1964 |
| Low | 7 | 0.00157 | 4 | 0 - 24 |

Regardless of whether these analyses show any evidence of significant differences between the methods, we will explore whether there is any systematic bias in the estimate of the alternative method compared to the gold standard method (e.g. the alternative method consistently over- or underestimates the EIR). If systematic bias is found, ecological log-linear regression models will be used to predict the result of the gold standard method using data from the alternative method, allowing adjustment for bias. Final models will then be prospectively validated in independent test sets to insure their accuracy.

# 8.0 DATA MANAGEMENT

Entomological data will be recorded by Ugandan fieldworkers on standardised data forms. The entomologist (KM) will ensure that this information is checked in the field by the team member who collected it, or a colleague working in the same geographical area. The forms will be sent to Kampala in batches where they will also be double entered by two different data entry clerks. The first and second entry datasets will be combined and errors corrected to produce a single dataset. This will be submitted to consistency checking by generic and study specific algorithms designed to identify sources of error. When inconsistencies are found, these will be checked against the original forms and subsequently amended in the dataset. If discovered that the error has been introduced during the data collection, this will be communicated to the entomologist / project manager by the data supervisor in Kampala. Errors will be corrected when possible, with checking in the field when necessary and possible to produce final datasets.

All forms with subject names will be kept in a locked cabinet, when not in use, and the key kept by the local investigators. These datasets will be, password protected and only accessed by the local investigators based at the study sites and the project data manager based in Kampala. Data will be stored for at least 10 years.

The Principal Entomologist (SL) will maintain appropriate medical and research records for this study in compliance with the principles of good clinical practice and regulatory and institutional requirements and in compliance of the requirements for the protection of confidentiality of participants. Only study team members will have access to these records.

Authorised representatives of the sponsor, the ethics committee(s) or regulatory bodies may inspect all documents and records required to be maintained by the investigator. The PI or designee will ensure the access to facilities and the records.

# 9.0 PROTECTION OF HUMAN SUBJECTS

This study is conducted in accordance with the principles set forth in the ICH Harmonised Tripartite Guideline for Good Clinical Practice and the Declaration of Helsinki in its current version, whichever affords the greater protection to the participants.

## 9.1 Institutional review boards (contact information redacted)

This protocol will be reviewed and approved by all IRBs before the project begins. Any amendments or modifications to this material will also be reviewed and approved by the IRBs prior to implementation. The IRBs will include:

***Makerere University, Faculty of Medicine Research and Ethics Committee (FOMREC)***

Address: PO Box 7072, Mulago Hospital, Kampala, Uganda

Contact Person: Dr. Charles Ibingira

Phone Number: +256 (0) 414-530020

Fax Number: +256 (0) 414-541036

***Uganda National Council of Science and Technology (UNCST)***

Address: Plot 3/5/7 Nasser Road, PO Box 6884, Kampala, Uganda

Contact Person: Mr. Julius Ecuru

Phone Number: +256 (0) 414-705500

Fax Number: +256 (0) 414-234579

***University of California, San Francisco, Committee on Human Research (UCSF CHR)***

Address: Office of Research, Box 0962, San Francisco, CA 94110, USA

Contact Person: Dr. Reese T. Jones

Phone Number: +1 (415) 476-1814

***London School of Hygiene & Tropical Medicine (LSHTM)***

Address: Keppel Street, London, WC1E 7HT, UK

Contact Person: Susan Sheedy

Phone Number: +44 (0) 20 7927 2256

Fax Number: +44 (0)20 7637 4314

Email: [susan.sheedy@lshtm.ac.uk](mailto:susan.sheedy@lshtm.ac.uk)

## 9.2 Informed consent process

Approval from local leaders will be sought before beginning activities in the project area. Heads of households or adult household representative will be asked to provide their consent prior to study activities being conducted in households. Informed consent documents translated into the local language will be provided, describing the purpose of the project and the procedures to be followed, and the risks and benefits of participation.

For the human landing catches, pyrethrum spray collections, and environmental measures, and CDC light catches witnessed written consent will be sought. The entomology field team will seek informed consent from a representative of the household, either the head of household (regardless of age), or other adult resident (over 18 years of age), for participation in the entomology surveys. The informed consent discussion will be conducted with the resident at their household by the study team in the language that the resident is most comfortable with, using a translator if necessary. If the household resident cannot read, an impartial witness will be present during the consent process. During the consent discussion, the appropriate informed consent form will be reviewed with the household resident and all contents will be discussed. The household resident will be offered a copy of the informed consent form to read during the discussion. Each section of the information sheet will be read to the household resident as it is written, and then further explained if necessary. Following the informed consent discussion, household representatives will be asked by the study personnel to provide their written consent on the approved informed consent document to participate in a research study. If the household representative is unable to read or write, their fingerprint will substitute for a signature, and a signature from a witness to the informed consent procedures will be obtained. No incentives or coercion will be used by study staff to obtain consent and efforts will be made to avoid undue coercion by others. All will be informed that they may withdraw from the study at any time without penalty.

##

## **9.3 Risks and discomforts**

**9.3.1 To households**

Potential risks and discomforts to participating households include loss of privacy, but this will likely be minimal. Care will be taken to protect the privacy of participating households, as described in this protocol. However, there is a risk that others may inadvertently see participants’ information, and thus their privacy compromised. Intrusion by the study staff into the household, and discomforts related to the study procedures, such as the pyrethrum spray, are other concerns. Study personnel will be instructed to interact with the households in a courteous and respectful manner in order to limit this possible discomfort.

**9.3.2 To field workers**

Human-landing catches have been used for estimating exposure in previous studies in the proposed study sites in Uganda [2]. Men/collectors participating in the human landing catches are at risk of being bitten by an infected mosquito and contracting malaria. To limit this possibility, we will follow WHO guidelines and ensure that field workers *’…take an appropriate and effective antimalarial prophylaxis to avoid contracting malaria during collection of biting mosquitoes’.* We will also ensure that mosquitoes will be collected as soon as they settle on the skin, before they feed. Importantly our collection schedule will follow the normal pattern of human behaviour with outdoor collections being made only from 18.00-21.50h. Indoor collections will be made throughout the night by men resident in the study sites. In this way, collectors will receive fewer bites than normal. Field workers will also be offered prompt medical care through the HC IV should they fall sick during their employment.

## 9.4 Benefits

No direct benefits to the individual or households will be achieved from the entomology exercise.

## 9.5 Confidentiality

Each participant will have a unique identification (ID) number. All data recorded on individuals will be made by recording the anonymous ID. All forms and datasets, apart from those for enumeration, will identify subjects by their subject specific ID and names will not be collected or entered. These datasets will be, password protected and only accessed by senior study staff and the study Data Manager. Data will be stored for at least 10 years.

## 9.6 Compensation

Households will be paid a small stipend equivalent to rent for the periods their households are used for study activities.

# 10.0 REFERENCES

1. O'Meara, W.P., et al., *Changes in the burden of malaria in sub-Saharan Africa.* Lancet Infect Dis, 2010. **10**: p. 545-55.

2. Okello, P.E., et al., *Variation in malaria transmission intensity in seven sites throughout Uganda.* Am J Trop Med Hyg, 2006. **75**: p. 219-225.

3. Pluess, B., et al., *Indoor residual spraying for preventing malaria (Review).* The Cochrane Review, 2010.

4. Lengeler, C., *Insecticide-treated bed nets and curtains for preventing malaria (Review).* Cochrane Review, 2009.

5. WHO, *WHO position statement on integrated vector management.* Week Epidem Rec, 2008. **20**: p. 177–184.

6. WHO, *Malaria entomology and vector control. Learner’s Guide*. WHO/CDS/CPE/SMT/2002.18 Rev.1. Part I ed. 2003, Geneva: World Health Organization.

7. Lindsay, S.W., et al., *Exposure of Gambian children to Anopheles gambiae malaria vectors in an irrigated rice production area.* Med Vet Entomol, 1995. **9**: p. 50-58.

8. Geissbühler, Y., et al., *Interdependence of domestic malaria prevention measures and mosquito-human interactions in urban Dar es Salaam, Tanzania.* Malaria Journal, 2007. **6**(1): p. 126.

9. Bayoh, N.M., et al., Anopheles gambiae*: historical population decline associated with regional distribution of insecticide-treated bed nets in western Nyanza Province, Kenya.* Malaria J, 2010. **9** e62.

10. Gilles, H.M. and D.A. Warrell, *Bruce-Chwatt's Essential Malariology.* 3rd ed. 1993, London: Edward Arnold.

11. Smith, D., et al., *Revisiting the basic reproductive number for malaria and its implications for malaria control.* PLoS Biology, 2007. **5**: p. e42.

12. Nauen, R., *Insecticide resistance in disease vectors of public health importance.* Pest Manag Sci, 2007. **63**: p. 628-33.

13. Muller, P., et al., *Field-caught permethrin-resistant Anopheles gambiae overexpress CYP6P3, a P450 that metabolises pyrethroids.* PLoS Genet, 2008. **4**: e1000286.

14. Ramphul, U., et al., *Insecticide resistance and its association with target-site mutations in natural populations of Anopheles gambiae from eastern Uganda.* Trans R Soc Trop Med Hyg, 2009.

15. Wondji, C.S., et al., *Two duplicated P450 genes are associated with pyrethroid resistance in Anopheles funestus, a major malaria vector.* Genome Res, 2009. **19**: p. 452-9.

16. Hemingway, J. and H. Ranson, *Insecticide resistance in insect vectors of human disease.* Annu Rev Entomol, 2000. **45**: p. 371-91.

17. Sharp, B.L., et al., *Seven years of regional malaria control collaboration--Mozambique, South Africa, and Swaziland.* Am J Trop Med Hyg, 2007. **76**: p. 42-7.

18. Sharp, B.L., et al., *Malaria vector control by indoor residual insecticide spraying on the tropical island of Bioko, Equatorial Guinea.* Malar J, 2007. **6**: p. 52.

19. N'Guessan, R., et al., *Reduced efficacy of insecticide-treated nets and indoor residual spraying for malaria control in pyrethroid resistance area, Benin.* Emerg Infect Dis, 2007. **13**(2): p. 199-206.

20. Ramphul, U., et al., *Insecticide resistance and its association with target-site mutations in natural populations of Anopheles gambiae from eastern Uganda.* Trans Roy Soc Trop Med Hyg, 2009. **103**: p. 1121-1126.

21. Scott, J.A., W.G. Brogdon, and F.H. Collins, *Identification of single specimens of the Anopheles gambiae complex by the Polymerase Chain Reaction.* Am J Trop Med Hyg, 1993. **49**: p. 520-529.

22. Gillies, M.T. and B. DeMeillon, *The Anophelinae of Africa south of the Sahara (Ethiopian zoogeographical region)*. 1968, Johannesburg, South Africa: The South African Institute for Medical Research.

23. Gillies, M.T. and M. Coetzee, *A supplement to the Anophelinae of Africa south of the Sahara.* 1987, Johannessburg: The South African Institute for Medical Research.

24. Wirtz, R.A., et al., *ELISA method for detecting Plasmodium falciparum circumsporozoite antibody.* Bull World Health Organ, 1989. **67**: p. 535 - 542.

25. Burkot, T.R., W.G. Goodman, and G.R. DeFoliart, *Identification of mosquito blood meals by enzyme-linked immunoabsorbent assays.* Am J Trop Med Hyg, 1981. **30**: p. 1336-1341.

# Appendix 1. Sample timetable of weekly activities

| Activity/site | M | T | W | T | F |
| --- | --- | --- | --- | --- | --- |
| Week 1 | | | | | |
| Human landing catches (2 houses/site) | X | X | X | X |  |
| Light trap installation (12-13 houses/night; 50/week) | X | X | X | X |  |
| Processing of HLC specimens (identification, Sp ELISA) |  | X | X | X | X |
| Light trap catches (2 houses/night) | X | X | X | X |  |
| Processing of LTC specimens (identification, Sp ELISA) |  | X | X | X | X |
| Exit trap installation (2 houses/site) | X | X | X | X | X |
| Exit trap collection (2 houses/site) | X | X | X | X | X |
| Pyrethrum spray catches (2 houses/site) | X | X | X | X | X |
| Processing of ETs & PSC (identification & BM ELISA) |  | X | X | X | X |
| Week 2 | | | | | |
| Human landing catches (2 houses/site) | X | X | X | X |  |
| Light trap installation (12-13 houses/night; 50/week) | X | X | X | X |  |
| Processing of LTC specimens (identification, Sp ELISA) |  | X | X | X | X |
| Week 3 | | | | | |
| Human landing catches (2 houses/site) | X | X | X | X |  |
| Light trap installation (12-13 houses/night; 50/week) | X | X | X | X |  |
| Larval surveys of study site | X | X | X | X | X |
| Week 4 | | | | | |
| Human landing catches (2 houses/site) | X | X | X | X |  |
| Light trap installation (12-13 houses/night; 50/week) | X | X | X | X |  |
| Processing of LTC specimens (identification, Sp ELISA) |  | X | X | X | X |
