# Supplementary material for: Measures of Malaria Burden after Long-Lasting Insecticidal Net Distribution and Indoor Residual Spraying at Three Sites in Uganda: A Prospective Observational Study
Source: PLoS Med. 2016 Nov 8;13(11):e1002167. doi: 10.1371/journal.pmed.1002167 (PMC5100985; doi:10.1371/journal.pmed.1002167)
Supplement: S4 Text — (DOC) [file pmed.1002167.s009.doc]

**Research Project 1:** **Optimizing strategies for malaria surveillance and measuring the impact of control interventions**

**A. SPECIFIC AIMS**

The epidemiology of malaria in Africa is in transition, with evidence of declining transmission and disease burden [1-3], a presumed consequence of expanded intervention coverage of insecticide-treated bednets (ITNs), indoor residual spraying (IRS) and effective case management with artemisinin-based combination therapies (ACTs). To date, most of the documented successes in malaria control in Africa have been limited to relatively low transmission settings or defined geographic areas such as islands. There has been very little scientific effort to document the transitioning epidemiology in areas of high malaria transmission using validated metrics of intervention coverage, infection risk, and disease burdens within a robust framework of malaria surveillance. Reliable surveillance is essential for the design, refinement and resource allocation of control programs and to provide assessment of the impact and cost-effectiveness of program activities. However, the capacity to conduct high-quality malaria surveillance is currently inadequate in much of Africa. One reason for this situation is the lack of financial and technical resources available to control programs for surveillance activities. Consequently, health information system (HIS) data are subject to the vagaries of incomplete data and inaccurate diagnosis. Furthermore, data on vector-based measures of transmission intensity and host-based measures of infection and disease are not collected routinely.

A further reason for the current inadequate surveillance of malaria in Africa is a lack of understanding of the optimal approaches to routine malaria surveillance. The objectives of this project are to establish and validate simplified, reliable, and cost-effective surveillance tools at various sites across Uganda. This work aims to inform malaria control efforts in Uganda and to provide a model for malaria surveillance in other malaria endemic countries. Our specific aims will be as follows.

**Specific Aim 1. To identify optimal strategies for malaria surveillance in Uganda by comparing different methodologies in multiple sites with varied transmission intensity.** We will perform comprehensive malaria surveillance studies at 3 sites in Uganda (Kabale – low transmission; Mubende – medium transmission; Apac – high transmission) to measure key metrics of malaria transmission (entomological inoculation rates), infection (parasite rates), and disease (measures of malaria morbidity and mortality). Surveillance methodologies using “streamlined” techniques designed to improve efficiency and feasibility will be compared with “gold standard” techniques, with the goal of identifying optimal strategies for surveillance under a range of epidemiological settings and with changing transmission intensity.

**Specific Aim 2. To estimate the impact of key malaria control interventions on measures of transmission intensity, infection, and disease using surveillance data at multiple sites in Uganda.** We will prospectively assess the coverage of key malaria control interventions (ITNs, IRS and ACTs) and measure metrics of malaria transmission, infection, and disease at multiple sites in Uganda. Statistical techniques will be used to estimate the impact of changing coverage levels of control interventions on our outcome measures. Over the course of the project, with streamlining of surveillance techniques, we will expand the number of sentinel sites from 3 to 6 to increase the diversity of epidemiological settings and levels of control interventions.

**Specific Aim 3. To conduct an economic evaluation of malaria control interventions to identify the optimal coverage levels and mix of interventions at multiple sites in Uganda.** We will prospectively collect data on the coverage and costs of key malaria control interventions at multiple sites in Uganda with variable transmission intensity. We will then model the cost-effectiveness of different interventions and their combinations at selected coverage levels as measured by various key impact indicators and disability adjusted life-years averted to determine the optimal level of coverage and mix of interventions to maximize health outcomes in different transmission settings.

The work outlined in this proposal will be implemented within our existing health facility-based malaria surveillance system in six districts in Uganda encompassing a range of transmission intensities. To augment this existing infrastructure and expertise and to facilitate our entry into new scientific areas, the proposed work will benefit from collaboration with the KEMRI-Wellcome Trust Research Programme, which has extensive experience and expertise in malaria epidemiology and community and school-based malaria surveys. We have also established collaborations with entomologists at the London School of Hygiene and Tropical Medicine and the Liverpool School of Tropical Medicine. To ensure that research findings are of direct relevance to ongoing malaria control efforts, proposed work will be integrated into ongoing activities and conducted in collaboration with the Uganda National Malaria Control Program and international funding agencies such as the President’s Malaria Initiative. Further, the proposed research will develop innovative approaches to malaria surveillance that should be widely applicable.

**B. BACKGROUND AND SIGNIFICANCE**

**B.1 Burden of Malaria in Sub-Saharan Africa and Uganda.** Malaria is Africa’s leading cause of mortality in children under five years of age and is responsible for 10% of the overall disease burden, 40% of public health expenditure, 30-50% of inpatient admissions, and up to 50% of outpatient visits (www.rbm.who.int). There are several reasons why Africa bears a large proportion of the world's malaria burden. First, most malaria infections in sub-Saharan Africa are due to *Plasmodium* *falciparum*, the cause of the most difficult to treat and severe form of the disease. Second, this region is also home to the most efficient malaria mosquito vectors, members of the *Anopheles gambiae* complex and those of the *An. funestus* complex. Third, most African countries are "the poorest of the poor", lacking the basic infrastructure and resources necessary to mount sustainable malaria control efforts. Uganda, where the proposed work would be located, is emblematic of the immense problem that malaria poses for African countries. Malaria is endemic in over 95% of the country, with some of the highest malaria transmission intensities reported in the world [4]. According to the 2008 World Malaria Report, Uganda ranked 6^th^ in terms of number of malaria cases and 3^rd^ in terms of number of malaria deaths [5]. Malaria is the leading cause of morbidity and mortality in Uganda and is responsible for up to 40% of all outpatient visits, 25% of all hospital admissions and 14% of all hospital deaths (Uganda Ministry of Health, unpublished). The overall malaria-specific mortality is estimated to be between 70,000 and 100,000 child deaths annually in Uganda, a death toll that far exceeds that of HIV/AIDS [6]. A 1995 Burden of Disease study indicated that 15% of life years lost to premature death was due to malaria and that families spend 25% of their income on malaria prevention and treatment (Uganda Ministry of Health, unpublished). Poor school performance and absenteeism due to malaria reduce chances of escaping from poverty [7]. Poor people tend to live in environments conducive to mosquito breeding and malaria transmission. Thus malaria enhances poverty, which in turn causes poor disease management, locking people in a malaria-poverty trap [8].

Despite the overwhelming burden imposed by malaria in Africa, there is increasing optimism that the tide can be turned through the establishment of several recent large-scale initiatives. The United States government has recently launched the President’s Malaria Initiative (PMI), with the goal of reducing malaria-related deaths in selected countries, including Uganda, by 50% within five years. Through PMI and other large funding sources, such as the Global Fund to Fight AIDS, Tuberculosis and Malaria (GFATM), Uganda and other African countries now have an unprecedented opportunity to reduce malaria associated morbidity and mortality on a national scale.

**B.2 Epidemiology of Malaria in Sub-Saharan Africa.** Malaria in sub-Saharan Africa is largely characterized by high rates of infection and the development of partial immunity after repeated exposure. Transmission intensity ranges from under ten to several hundred infective bites per person year, but the majority of these infections do not lead to clinical illness. The risk of developing symptomatic disease is inversely proportionate to the level of acquired immunity. Partial immunity develops through repeated exposure, leading first to protection against severe forms of disease, followed by protection against symptomatic illness [9]. The result of this phenomenon is that the burden of malaria in Africa is heavily borne by young children. Newborns are protected during the first few months of life due to the transplacental acquisition of maternal antibodies and relatively high hemoglobin F content in the infants’ erythrocytes [10]. After about 3-4 months of age, protection from these factors wanes, and the infant becomes highly vulnerable to malaria, including severe disease, with an estimated 75% of malaria deaths occurring in African children under the age of 5 years [11]. However, the age at which malaria risk peaks in endemic areas of Africa varies from 1-2 years of age in areas of high transmission intensity to approximately 5 years of age in areas of low to moderate transmission intensity [12]. The risk of symptomatic malaria declines as children get older, however, school aged children are at highest risk of asymptomatic parasitemia, with chronic parasitemia resulting in anemia [13-15]. Malaria parasitemia may also impact significantly on the education and learning of school-aged children [13]. However, as the intensity of malaria transmission declines due to control efforts, it is suggested that clinical immunity will be acquired more slowly and disease burdens will shift into older age groups [16]. In addition, the high prevalence of parasitemia in children (up to 90% at any time) creates a large reservoir for transmission [17]. Our proposed studies will benefit from the extensive experience we have gained studying malaria in Uganda and elsewhere in East Africa. Lessons learned in Uganda will also be relevant for other African nations.

**B.3 Malaria Control in Africa and Uganda.** Proven effective malaria control interventions currently available in Africa include ITNs, IRS, and effective case management with ACTs. In controlled trials, ITNs have been associated with a 17% reduction in child mortality and a 50% reduction in the incidence of clinical malaria [18]. One challenge for African countries is to increase ITN coverage, especially amongst high risk populations. According to the Ugandan 2006 Demographic and Health survey only 10% of children under the age of 5 years were sleeping under an ITN. Currently, large scale efforts are underway to increase ITN coverage in Uganda, with a goal of reaching 85% of children under 5 years by 2010 (PMI Malaria Operational Plan 2007). Uganda, like many other African countries, has renewed efforts to provide vector control through the use of IRS of insecticide. In 2006, Uganda began a successful IRS program in low endemicity highland areas of the country; however these areas represent less than 5% of the Ugandan population. Uganda’s IRS program has been expanded to two high transmission districts, as well as internally displaced people’s camps in northern Uganda. However, it is unknown whether IRS is effective or sustainable in high transmission settings. The cornerstone of childhood malaria control for much of Africa has been the provision of prompt and effective antimalarial therapy (case management). However, the spread of resistance to inexpensive drugs has seriously compromised this strategy. Newer ACTs have been shown to be highly effective and have been adopted in most African countries, but these drugs are considerably more expensive than older therapies, and their availability remains limited. As these newer therapies become more widely available, it is important to use them in a rational way to prevent the further spread of drug resistance [19]. Based on the considerations noted above, there is no clear single path for improved malaria control in Africa. Rather, improvements in control will likely come from incremental advances through expanding and better targeting the coverage of proven interventions, as well as the development of new interventions. For the first time, resources are becoming available to fund the expansion of existing interventions, however, there is a critical need to generate an evidence base to support the targeted use of control interventions to maximize their impact.

**B.4. Malaria Surveillance.** Malaria **s**urveillance, which encompasses monitoring and evaluation of malaria control efforts, is essential to guide program planning and management, inform governments and donors on progress, and assist with advocacy. Surveillance and monitoring provides the basis for the design, refinement and resource allocation of control programs [20, 21], while evaluation provides assessment of the impact and cost-effectiveness of program activities [22]. Most malaria control programs rely on routinely collected health facility-based data for surveillance needs. These data are inadequate to describe a changing clinical epidemiology, subject as they are to vagaries of incomplete data and poor diagnostics, and very few contemporary studies have tested the reliability of national case surveillance activities [23]. Furthermore, at a time when malaria transmission and disease burdens are in decline, with targets set for elimination or low stable endemic control, there is an important scientific requirement to design and implement comprehensive surveillance activities including monitoring sensitive metrics of infection risk, intervention coverage and disease outcome. A practical approach to this problem is to identify a few key components of surveillance and implement these effectively, in a timely manner, and throughout the country. Defining the risks of *P. falciparum* infection and disease can be measured using a variety of techniques in different populations, but their cost and sensitivity depend on the overall levels of risk and the sampling strategies adopted [24]. Below we discuss current approaches to the different components of malaria surveillance in sub-Saharan Africa and provide the rationale for the selecting the methods to accomplish the specific aims of this proposal.

**B.4.1 Measures of Transmission.** The exposure of individuals to malaria parasites is typically represented as the Entomological Inoculation Rate (EIR), which is an estimate of the number of infective bites received during a season or annually. It is derived by multiplying the number of bites received each night by the proportion of mosquitoes that have sporozoites, and again with the number of nights during the period of interest. It is a fundamental measure that will allow us to determine whether the control measures are successful or not, as well as allowing us to interpret the pattern of malaria infection and morbidity we find. Classically, human-landing catches have been used to estimate EIR. Here men collect biting insects landing on their exposed limbs. Since this method of sampling exposes individuals to the risk of vector-borne diseases, it is important to replace it with other techniques. We propose to move in this direction by directly comparing human-landing catches with two standard collection methods including: (1) light trap collections made indoors and (2) pyrethrum spray collections together with window traps collections. Whilst we recognize that other collection methods are currently being developed, they are still in the early phase of development, and we propose to limit ourselves to well-established sampling methods. A light trap positioned next to a person sleeping under a bednet provides a relatively simple method of estimating biting rates. We propose to use spray collections in homes in the early morning to collect bloodfed mosquitoes. Bloodmeals will then be analyzed to identify those bloodfeeding on people. If in addition mosquitoes are collected leaving the house in exit traps, an alternative estimate of EIR can be obtained by summing the number of human bloodfeds collected in the room and in the exit trap. In addition to the major malaria vectors within the *An. gambiae* and *An. funestus* complexes there are a number of secondary vectors, so it is important to quantify the total transmission pressure, not just that posed by endophagic/endophilic primary vectors [25].

**B.4.2. Measures of Infection.** The parasite rate (PR) is defined as the proportion of surveyed persons harboring parasites in their peripheral blood, and PR among children aged 2-10 years provides an indirect quantitative measure of transmission intensity across a range of malaria endemicities [26]. PR is the most frequently measured malariometric index. The usefulness and robustness of malaria surveillance to generate estimates of PR will depend on the sampling strategy. The most robust sampling framework for national malaria surveys are household cluster surveys which collect information on malaria intervention coverage, patterns of antimalarial use, and in some cases, on the prevalence of malaria infection among pregnant women and children under five years. However, estimating PR among these age groups is not optimal, as pregnant women sequester infections [27] and infection prevalence in very young children is modified by a variety of factors, including presence of maternal antibodies [28]. More importantly, national cluster surveys are expensive, time-consuming, and technically complicated to undertake, and sampling is typically powered to provide only national or first-level administrative unit representative estimates of malaria risk and intervention coverage. Such limitations preclude frequent monitoring and evaluation, especially at local levels, hindering decentralized planning and allocation of resources for targeted control. Increasing the frequency of monitoring enables prompt feedback of intervention effectiveness, helping control programs to adapt and improve control strategies. Uganda has a relative paucity of data on PR, with only 94 surveys conducted since 1985, in contrast to its neighbors Kenya and Tanazania, where 987 and 773 surveys have been conducted over this same time period, respectively ([www.map.ox.ac.uk/data/](../www.map.ox.ac.uk/data/)).

A complementary, inexpensive framework for malaria planning, monitoring and evaluation is offered by school malaria surveys [29-31]. The practical advantages of sampling children at school are clear: identification and selection of individuals is simplified, compliance is high, and costs are reduced, since only a fraction of the population is examined. In addition, ministries of education are increasingly developing or upgrading national school databases using geographical information systems (GIS). This allows incorporation of information on school locations and enrolment into a single database, and the use of geo-statistical methods to model risks between schools and across un-sampled schools. There are also important epidemiological reasons for sampling children who attend school. Historically, malaria endemicity was defined on the basis of PR among children aged 2-10 years, and geographical reconnaissance of malaria was recommended in all areas prior to a control program launching into an attack phase of the Global Malaria Elimination Program [24]. Recent mathematical models of malaria transmission dynamics indicate that EIR determines both the rate at which PR rises during early childhood and the age at which maximum PR is attained [32]. Age-stratified studies in varying transmission settings reveal that PR consistency has a convex relationship with age, with PR rapidly rising among young children, attaining a maximum within the 5-10 year old age class, and declining among adolescents and young adults, thereafter maintaining a relatively stable low value throughout adulthood [26]. Such consistency in the relationship between PR and age permits age-standardization of available *PR* estimates to the 2 to 10 year age range on the basis of catalytic conversion models [26], although estimates are most precise when the majority of the sampled population are within this age range.

**B.4.3 Measures of Disease.** One of the most important goals of any malaria surveillance program is estimating the number of malaria cases. The general working definition of a case of malaria is considered to be “fever with malarial parasitemia”, which normally defines all patients that require antimalarial treatment [5]. The number of reported cases of malaria is generally captured through a country’s health management information system (HMIS). However, the accuracy of these data are often limited due to incomplete reporting, failure of the system to capture cases that occur outside the formal health care system, and the absence of laboratory confirmation, particularly in African countries where the vast majority of reported cases of malaria are not laboratory confirmed [20, 33]. Indeed, national reports on trends in malaria cases from most countries in sub-Saharan Africa, including Uganda, simply reflect the number of cases of fever captured through HMIS system [5]. Another problem with monitoring trends in malaria cases is the lack of a clear denominator. As malaria is a disease that can occur repeatedly, the best metric for estimating malaria morbidity is malaria incidence, defined as the number of confirmed malaria cases per person per time at risk. The most accurate method of estimating malaria incidence involves longitudinal studies in defined cohorts, where all cases of suspected malaria (i.e. those with fever) are captured and undergo a diagnostic test that is highly sensitive and specific. However, such methods require considerable resources and are rarely undertaken as part of routine malaria surveillance programs. The slide positivity rate (SPR), defined as the number of laboratory-confirmed malaria cases per 100 suspected cases examined, provides an alternative method for estimating temporal changes in malaria incidence. The SPR gains accuracy in considering only laboratory confirmed cases of malaria, and it can provide a rapid and inexpensive means of assessing the burden of malaria in a population utilizing health care facilities. Decreases in the SPR have been cited as evidence for successful malaria control interventions in Africa [2, 3], yet there has been little work describing the relationship between changes in SPR and changes in malaria incidence using empiric data.

Estimating trends in malaria-associated mortality in sub-Saharan Africa also faces a number of methodological challenges and limitations. Representative household surveys (i.e. Demographic Health Survey) can provide high quality data on all cause mortality, but generally do not collect data on malaria-specific mortality and are limited by high costs, infrequency (typically every 4-8 years), and sampling over large areas, limiting geographic precision. Mortality surveys can be supplemented with verbal autopsy (VA) to provide malaria-specific mortality data, however, misclassification of deaths is a problem and further validation of these techniques are needed [34]. Health facility sentinel sites have also been used to examine trends in malaria-associated mortality, but are potentially limited by a lack of representativeness, limited sensitivity (many deaths occur at home) and absence of laboratory confirmation. Despite low sensitivity, facility-based trends would reflect true mortality trends in the community if sensitivity and positive predictive values were constant [34]. One could also follow trends in the proportion of all deaths reportedly caused by malaria, as this measure may be less susceptible to bias due to changing health facility use. With expanded efforts to reduce the burden of malaria, improved surveillance systems designed to accurately measure trends in malaria morbidity and mortality are needed, both at the level of the health facility and the community.

**B.5 Measuring the Impact of Malaria Control Interventions.** Evidence supporting the use of key malaria control interventions (ITNs, IRS, and ACTs) in sub-Saharan Africa is highly variable. The efficacy of ITNs has been well established through a number of randomized controlled trials (RCTs) conducted across a wide range of epidemiological settings in Africa. Pooled estimates report a 50% reduction in the incidence of malaria and a 17% reduction in all cause childhood mortality associated with ITNs [18]. Similar results have been seen with large scale ITN distribution campaigns under operational conditions [35-37]. In contrast to ITNs, there are limited data from controlled trials on the efficacy of IRS, however many studies have documented the effectiveness of IRS in certain settings by comparing data before and after the intervention [38]. In sub-Saharan Africa, a number of malaria eradication pilot projects were initiated from the 1950s to the 1970s, with significant reductions in transmission and malaria cases, although in most cases transmission was not interrupted [39-41]. However, with few exceptions, IRS was not taken to scale during this period and interest in IRS waned. More recently, there has been a renewed interest in IRS as a major malaria control intervention in sub-Saharan Africa, with reports of significant declines in the burden of malaria following IRS in areas of low-moderate transmission intensity or discrete communities such as South Africa [42], the highlands of Kenya [43], and Bioko Island, Equatorial Guinea [44]. The WHO is now promoting wider application of IRS, including use in highly endemic settings in sub-Saharan Africa [38]. However, there are limited data supporting the effectiveness of IRS in high transmission settings, and historical data and modeling exercises suggest that several years of IRS may be needed to have an impact [45, 46]. There is also an urgent need to generate data on the operational effectiveness of IRS in different epidemiological settings, and to evaluate the potential impact of insecticide resistance on control. With respect to case management, the WHO now recommends ACTs for the treatment of uncomplicated malaria. There is a large body of evidence from RCTs documenting excellent efficacy of ACTs in Africa [47]. There is also evidence from non-controlled studies documenting significant declines in measures of malaria burden following the wide-scale implementation of ACTs in combination with vector control interventions in South Africa [42] and the island of Zanzibar [1]. It has also been suggested that wide scale use of ACTs may lead to decreases in transmission intensity due to the anti-gametocyte effects of these drugs in relatively low transmission settings [48], but this impact may be less pronounced in higher transmission settings typical of sub-Saharan Africa [49]. In summary, there is a substantial body of evidence supporting the widespread use of IRS, ITNs, and ACTs in sub-Saharan Africa. However, considerable gaps in knowledge remain, such as the impact of these interventions in combination in operational settings under different epidemiological conditions, in various sequences and combinations, and over an extended period of time. The World Health Organization is advocating Integrated Vector Management (IVM), where a combination of tools are used to reduce malaria, yet there is little evidence to support which strategies work best in different eco-epidemiological settings. This study will provide important insights into which combinations are effective at reducing malaria over several years. As the vast majority of malaria control interventions are not implemented as controlled experiments, it is important to develop standardized metrics and analytical methods for estimating their impact through routine surveillance.

**B.6 Economic Evaluation of Malaria Surveillance and Control.** Economic evaluation compares the costs and consequences of competing alternative interventions to allow policy makers to make informed and rational decisions on the allocation of scarce resources. Different methods of economic evaluation are available; cost-effectiveness analysis (CEA) is most commonly used in the health sector. In CEA, the costs of different interventions are assessed per unit of health effect. In cost-utility analysis, which may be considered a subset of CEA, health is measured in terms of standardized increments, such as the disability-adjusted life year (DALY). The DALY, which captures the duration of time lived with a disability and the time lost due to premature mortality, has gained favor as the standard indicator of disease burden and is used widely in evaluations of malaria interventions [50, 51]. A number of studies have evaluated the cost-effectiveness of malaria control interventions, but gaps in the evidence remain. In 1999, a review of 14 studies concluded that the evidence on the cost-effectiveness of malaria prevention and treatment in Africa (including ITNs, IRS, IPTp, a hypothetical vaccine, and changing the first line drug for treatment) was limited [52]. Since then, multiple studies have evaluated the costs or cost-effectiveness of use of insecticide-treated bednets [53-55], indoor residual spraying [55-57], antimalarial treatment [58-63], malaria case management [64], malaria diagnostics [65-71], and intermittent preventive treatment [72-75], building the evidence base on malaria control interventions. Although these analyses suggest that interventions for the prevention and treatment of malaria are highly cost-effective in sub-Saharan Africa, including provision of ITNs (US$ 5-17 per DALY averted) and IRS (US$ 9-24 per DALY averted), CEA results are context specific and may not be generalizable to policy debates in other settings. Data on the costs and consequences of interventions, alone and particularly in combination, in different epidemiological settings, remain limited. Few studies based on Ugandan data are available and rapid change in the context of malaria control in terms of availability and coverage of interventions, epidemiological burden, spread of antimalarial resistance, and changes in the cost of interventions, calls for additional studies to inform policy decision-making and ensure that resources are allocated efficiently.

**C. PRELIMINARY STUDIES**

**C.1 Uganda Malaria Surveillance Program (UMSP).** UMSP was established by our collaboration and the Uganda Ministry of Health in 2001 with the purpose of creating a multi-site surveillance system for monitoring antimalarial drug efficacy and safety, monitoring malaria related morbidity and mortality, and building capacity for facility based management of malaria, with the goal of providing sustainable progress in malaria control. One of the strengths of UMSP is the presence of an active health facility based sentinel site surveillance system involving several government heath centers across the country. In 2006, UMSP expanded the scope of the sentinel site surveillance system to collect detailed and high quality data on the burden of malaria in the outpatient setting. Individual level patient data, including patient demographics, results of laboratory tests, diagnoses given, and treatments prescribed, are collected using a standardized case record form for all patients who present to the outpatient department of the sentinel site health facilities. Data are entered electronically at the sites in real time and sent to a core facility in Kampala at the end of each month using cellular technology. At our core facility in Kampala data are cleaned and merged into a larger database which is accessible through a website ([www.mu-ucsf.org/UMSP](http://www.mu-ucsf.org/UMSP)).Our individual patient database allows us to correlate outcome variables and stratify data across demographic variables (i.e. age) and calendar time. For the research outlined in this proposal we will focus our activities in 6 districts where current UMSP surveillance sites are located. These sites consist of 2 districts with relatively low malaria transmission intensity (Kabale and Kanungu), 2 districts with medium transmission intensity (Mubende and Jinja), and 2 districts with high transmission intensity (Apac and Tororo). The sites see an average of 1,000-2,000 patients per month, with 30-48% of patients 10 years of age or younger. A majority of patients 10 years or younger are suspected of having malaria, ranging from 47% at the lowest transmission site to over 70% at the higher transmission sites. Among patients over 10 years of age, 29-61% are suspected of having malaria, with no difference in this level based on transmission intensity (Table 1).

**Table 1. Description of outpatient data from sentinel districts to be included in this proposal.**

| **District** | **Relative transmission intensity** | **Average monthly data July 2008 – June 2009** | | | |
| --- | --- | --- | --- | --- | --- |
|  |  | **Total patients seen** | **Age < 10 years (%)** | **Suspected malaria age < 10 years (%)** | **Suspected malaria age > 10 years (%)** |
| Kabale^*^  Kanungu  Mubende  Jinja  Tororo  Apac | Low  Low  Medium  Medium  High  High | 2073  1704  991  2382  2038  1527 | 613 (30%)  588 (35%)  356 (36%)  792 (33%)  984 (48%)  595 (39%) | 288 (47%)  396 (67%)  282 (79%)  516 (65%)  745 (76%)  427 (72%) | 417 (29%)  478 (43%)  390 (61%)  586 (37%)  433 (41%)  314 (34%) |

^*^ Data only available from September 2008-January 2009

One of the strengths of the UMSP health facility based malaria surveillance system is the high quality of care and reliance on laboratory confirmed diagnosis that is generally not available for most public health facilities in sub-Saharan Africa. Health care workers at all UMSP sentinel site heath facilities have undergone a two week integrated team based training program to improve malaria case management as well as periodic retraining by UMSP staff. This training program was associated with a significant improvement in the proportion of patients with suspected malaria referred for diagnostic testing, and documented over 90% sensitivity and specificity of field microscopy when compared to expert microscopy [76]. At the sentinel sites that are the focus of this proposal and where data are available for the previous 12 months, the proportion of patients with suspected malaria who are referred for diagnostic testing has now reached 80% at all the sites and over 94% at 3 of the sites (Figure 1).

**Figure 1. Proportion of patients with suspected malaria referred for diagnostic testing**

2008

2009

The slide positivity rate (SPR), defined as the number of laboratory-confirmed malaria cases per 100 suspected cases examined, has been advocated as a useful metric for monitoring trends in malaria morbidity [5]. The SPR can provide a rapid and inexpensive means of assessing the burden of malaria in a population utilizing health care facilities, and decreases in the SPR have been cited as evidence for successful malaria control interventions in Africa [2, 3]. Monthly SPR trends in children < 10 years of age from UMSP sentinel sites that will be the focus of this proposal are presented in Figure 2. There is a strong correlation between SPR and the level of transmission intensity, with ranges of 30-40% at the lowest transmission site to 60-80% at the highest transmission sites. Over the last 12 months there have been no clear changes in the SPR at any of the sites, with the exception of expected seasonal fluctuations. In summary, our collaboration is well positioned to carry out the health facility based outpatient surveillance studies outlined in this proposal. We have a number of well functioning government health facilities from a diverse range of epidemiological settings utilized by large numbers of patients, a high proportion of patients suspected of having malaria, and excellent diagnostic capabilities allowing us to focus on laboratory-confirmed cases of malaria.

**Figure 2. Slide positivity rate (SPR) for children < 10 years of age**

2008

2009

**C.2 Measuring the incidence of malaria in cohorts.** Our collaboration has been an innovative leader in longitudinal studies of malaria incidence in cohorts of children living in both urban and rural environments. Between 2005 and 2008 we conducted a prospective study in a cohort of children living in the relatively low transmission setting of Kampala, Uganda. Following a census of an urban slum, we randomly selected a representative sample of 690 children age 1-10 years. Study participants were asked to come to a designated study clinic, open 7 days a week, for all healthcare needs. Subjects who presented to the clinic with fever (tympanic temperature > 38.0°C) or reported history of fever symptoms in the previous 24 hours had blood obtained by fingerprick for a thick blood smear. If the thick blood smear was positive, the participant was diagnosed with and treated for malaria. If the thick blood smear was negative, the participant was not given antimalarial therapy. Study participants were seen routinely at least once a month to review the study protocol and discourage the use of outside medications. These methods allowed us to generate accurate estimates of malaria incidence (number of malaria episodes per person time) and correlate this outcome measure with other variables of interest (Figure 3) [77].

**Figure 3. Temporal trends in malaria incidence in a cohort of children living in Kampala, Uganda**


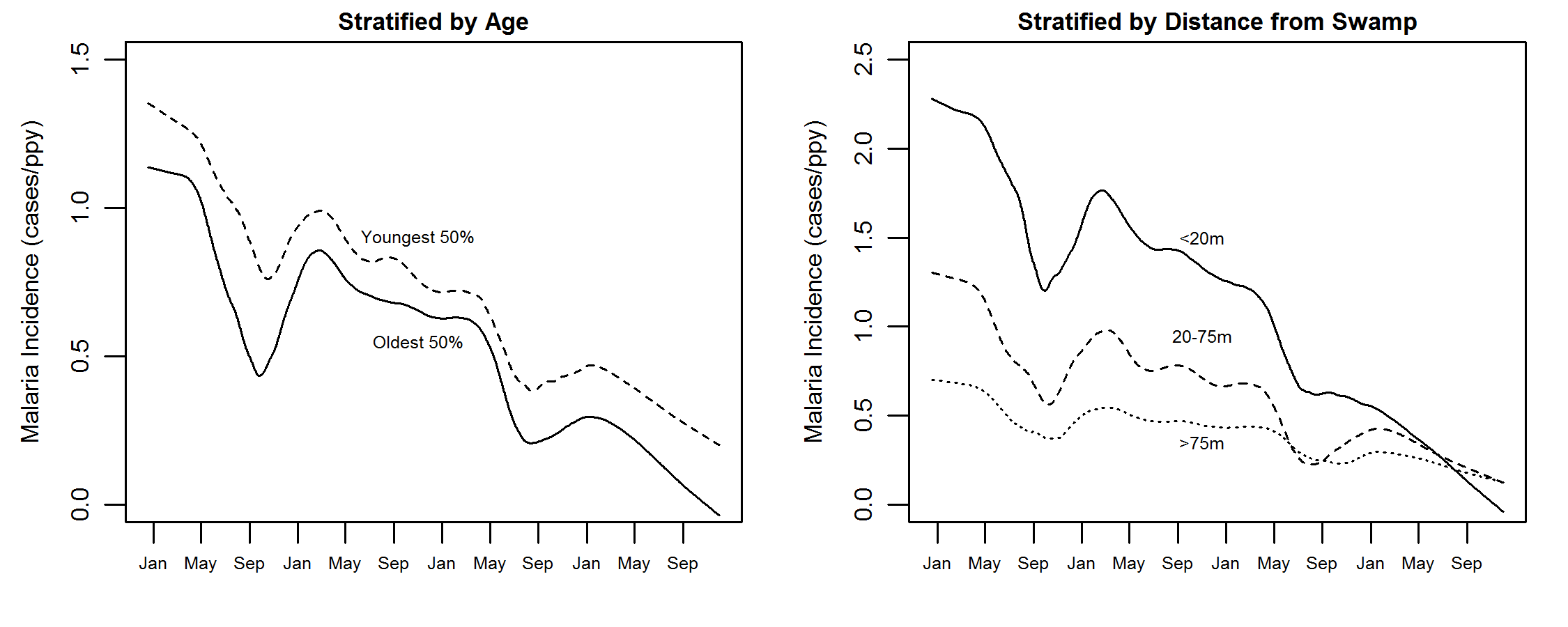


2005

2006

2007

2008

2005

2006

2007

2008

There was a greater than 3-fold decrease in the annual incidence of malaria over the 4 year follow-up period, with the most pronounced reduction following the distribution of ITNs to all study participants in May 2006. Malaria incidence was also associated with location of residence, with malaria incidence highest in those children living near a mosquito breeding area (swamp) that bordered the study area. These data demonstrate that well conducted cohort studies may capture marked changes in malaria incidence over a relatively short period of time and are particularly well suited for measuring the impact of interventions while controlling for other risk factors such as age and geography.

In 2007 we began a prospective study in a cohort of 350 infants living in a high transmission area in the Tororo District of Eastern Uganda. All study participants were given an ITN at enrollment and will be followed until 5 years of age using methods similar to those described above. The incidence of malaria in this cohort increased with age over the first 15 months of life (presumably due to the waning effects of maternal antibodies) before leveling off at over 5 episodes per year at 30 months of age. In addition, the incidence of malaria is over 3-fold higher in those living in a rural environment compared to those living in the town municipality (Figure 4). The incidence of malaria in Tororo is over 10-fold higher compared to similarly aged children in Kampala in the setting of uniform ITN use. In summary, the accurate measurement of malaria incidence in well defined cohorts can provide a wealth of information, but such studies require a considerable amount of expertise and a large commitment of time and resources. Our collaboration has been an innovative leader in prospective cohort studies and the lessons we have learned will be invaluable for the studies described in this proposal.

**Figure 4. Relationship between malaria incidence and age in a cohort from Tororo, Uganda**


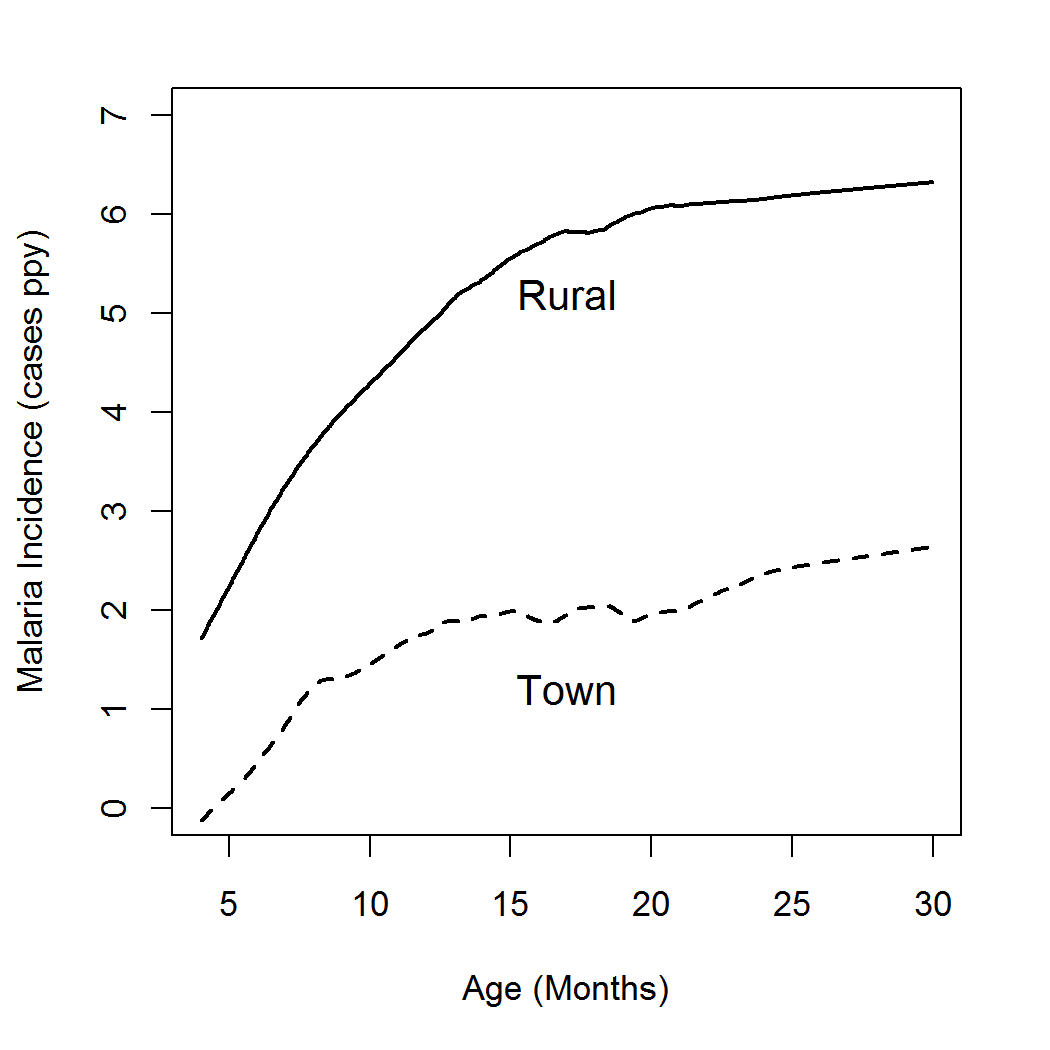


**C.3 Relationship between changes in malaria incidence and slide positivity rates.** As discussed above, the most accurate method of estimating malaria incidence involves longitudinal studies in defined populations, where all cases of suspected malaria (i.e. those with fever) are captured and undergo a diagnostic test that is highly sensitive and specific. However, such methods require considerable resources and are rarely undertaken as part of routine malaria surveillance programs. The slide positivity rate (SPR) provides an alternative method for estimating temporal changes in malaria incidence. The relative change in the incidence of malaria over time (r∆Im) can be defined as Im*_i+1_* / Im*_i_ ,* where *i* represents the baseline time interval and *i+1* represents the interval immediately following. Assuming that that there is no differential bias in the subgroup of patients with suspected malaria who undergo laboratory testing within a target population and the incidence of non-malaria fevers does not change over time, SPR can be used to estimate the relative change in malaria incidence over time by the following formula:

| Estimated r∆Im | = | Im *_i+1_* | = | SPR*_i+1_* (1 – SPR*_i_*) |
| --- | --- | --- | --- | --- |
|  |  | Im *_i_* |  | SPR*_i_* (1 – SPR*_i+1_*) |

Using data from our cohort of children living in Kampala (section C.2), we compared relative changes in malaria incidence that were directly observed with estimates that were derived from changes in the SPR using the above formula. Using 2-month time intervals the relative change in the incidence of malaria ranged from 0.27-2.71 for the observed values and 0.25-2.59 for the estimated values. Using 6-month time intervals the relative change in the incidence of malaria ranged from 0.35-1.80 for the observed values and 0.37-1.86 for the estimated values (Figure 5). The observed and estimated r∆Im had the same directionality in 22 of 23 (96%) 2-month intervals and 7 of 7 (100%) 6-month intervals. In summary, changes in SPR provided an accurate means of estimating changes in malaria incidence in this well defined cohort. The SPR has several advantages as a surrogate measure for changes in the incidence of malaria using routine surveillance data. Surveillance using SPR applies a consistent and accepted case definition of malaria, is easy to integrate into most health management information systems, and can be done at a fraction of the cost of measuring the true incidence of malaria. However, further research is needed to validate these finding in other epidemiological settings using real world health facility based surveillance data.

**Figure 5. Estimated versus observed temporal changes in malaria incidence**

**
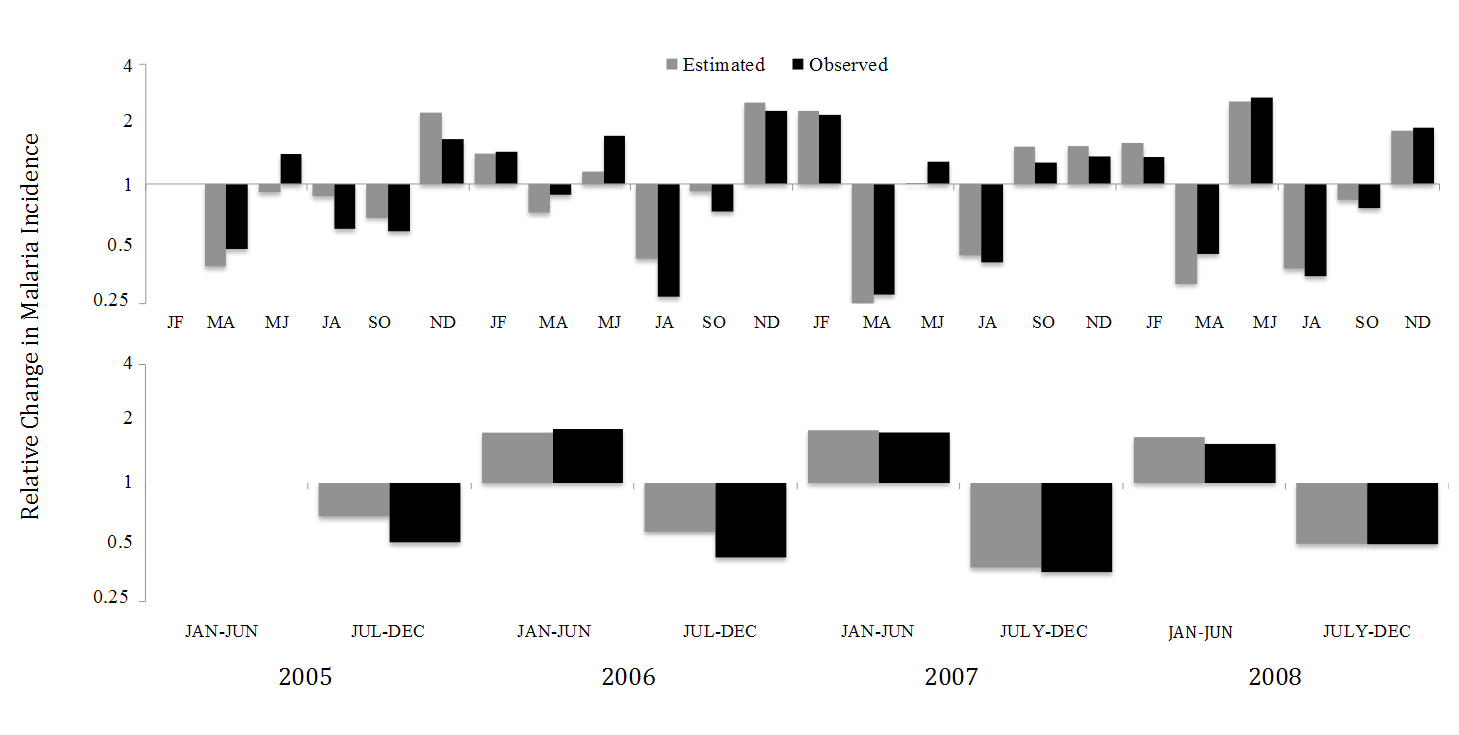
C.4 Inpatient Malaria Surveillance.** UMSP in collaboration with the Kenya Medical Research Institute carried out a retrospective review of inpatient records for children under 5 years of age for the year 2008 in the 6 district hospital which will be the focus of this proposal (Figure 6).

**Figure 6. Monthly average inpatient surveillance data for children < 5 years of age (2008)**

This retrospective review of hospital records allowed us to generated estimates needed for planning activities outlined in this proposal, but also indentified many limitations in the current system. Large numbers of children under 5 years of age were admitted to the hospital each month, and with the exception of our lowest transmission district (Kabale), the majority of under 5’s (71-81%) were given a diagnosis of malaria. However, for the vast majority of cases, the diagnosis of malaria was made without laboratory confirmation. The total number of deaths in children under 5 years admitted to the hospital ranged from 2-20 per month, with malaria accounting for 0-63% of deaths (malaria case fatality rate ranging from 0-4.2%). However, there were no standardized procedures in place for classifying the cause of death. In this proposal we plan to address many of these limitations by implementing standardized procedures for malaria diagnostics and classification of disease severity and outcomes (see section D.7)

**C.5 Measuring the Impact of Malaria Control Interventions.** There is an urgent need for data to assess the impact of malaria control interventions in operational settings. We measured the impact of IRS in 2 UMSP sentinel districts with very different transmission intensities using the slide positivity rate (SPR) in children under 5 years of age as our primary metric of malaria morbidity. In Kanungu, a district with a baseline EIR of < 10, indoor residual spraying (IRS) using lambda-cyhalothrin was implemented in February-March 2007 with 90% coverage of targeted households. IRS was associated with a substantial drop in SPR from 47% to < 15%, however after approximately 6 months the SPR began to increase, reaching pre-IRS levels after 2 years (Figure 7) [78]. In Apac, a district with a baseline EIR of > 1500, IRS using DDT was implemented in April-May 2008 with 92% coverage of targeted households. In contrast to Kanungu, there was no appreciable change in the SPR. Resistance phenotyping of the vectors in this area showed that they were highly resistant to DDT (John Morgan, personal communication). It should be noted that with the exception of the UMSP surveillance data there were no monitoring and evaluation programs in place to measure the clinical impact of IRS in these district due to a lack of resources, and data on the impact of IRS on measures of transmission intensity, infection and severe disease/mortality are lacking. These limited data highlight the need for comprehensive surveillance for measuring the impact of malaria control interventions and suggest that for IRS to be fully effective, a sustained program must be in place at lower transmission settings and repeated rounds of IRS may be needed in higher transmission settings before any clinical benefit becomes apparent. In addition, IRS programs will best pay attention to local insecticide resistance patterns.

**Figure 7. Changes in SPR following IRS in two districts with varying transmission intensity**

IRS

2009

2008

2007

2006

IRS

**C.6 Cost-Effective Analysis of Malaria Control Interventions.** Our group has previously collaborated with LSHTM on economic evaluations of malaria control interventions in Uganda, developing interactive models to evaluate use of rapid diagnostic tests (RDTs) for malaria diagnosis [65], and Uganda’s program of home-management of malaria (Lubell and Staedke, unpublished data) which are described below.

**C.6.1. Economic evaluation of RDTs.** Increasingly, RDTs for malaria are being considered for routine use in Africa. However, many RDTs are available and selecting the ideal test is challenging. The appropriateness of RDT implementation depends in part on patient population and epidemiological setting, and on policy makers’ priorities and preferences. We developed a model based on a decision-tree structure and cost-benefit framework, incorporating consequences of diagnosis and treatment to estimate the total costs, representing both expenditure and outcomes, for different management strategies. The interactive model is available online at <http://www.hefp.lshtm.ac.uk/publications/downloads/models/Lubell_RDT_MJ_online.xls>. Strategies compared in the model include case management based on the results of two available RDTs, one detecting histidine-rich protein 2 (HRP2) and one detecting plasmodium lactate dehydrogenase (pLDH), or presumptive treatment without a confirmed diagnosis. Data inputs were obtained from clinical evaluations of the two RDTs at seven sites in Uganda [79]. Applying the model in the Ugandan context demonstrates that when considering direct expenditure alone, the pLDH test is the preferred option for adult patients at all sites except in high transmission settings, while young children are best treated presumptively in all settings. When health outcomes are considered, the HRP2 test gains an advantage in almost all settings and for all age groups. When the model is adjusted to account for compromised adherence to test results, as has been observed in a number of studies [80, 81], the efficiency of using RDTs drops sharply. The outputs of the model vary by location, depending on factors such as transmission intensity and the particular costs and accuracies of the RDTs under consideration. Researchers and policy-makers can manipulate the values for the different variables in the model, to take account of local conditions, thus making the model relevant to their situation. Adaptable models such as this one can offer immediate policy recommendations that encourage the most efficient deployment of new technologies.

**C.6.2. Economic evaluation of home management of malaria (HMM).** To improve access to antimalarials, HMM programs are being promoted across Africa. However, data on the effectiveness of such community-based programs that distribute antimalarial drugs for presumptive treatment of malaria to febrile children are limited. The cost-effectiveness of HMM programs can be expected to vary widely with malaria transmission, access to medical facilities, and the cost and effectiveness of the antimalarial drugs delivered. In addition, health outcomes for children with other causes of illness who are incorrectly treated with antimalarials, and the societal costs associated with widespread use of antimalarials, such as the possible development of parasite resistance, must also be considered. We designed a Markov model to assess the cost-effectiveness of HMM programs in different transmission intensities and at varying levels of access to health care facilities. The data were obtained from Uganda where an HMM program has been in place since 2002. The preliminary analysis suggests that adoption of HMM programs is not always a cost-effective strategy. In some situations, where access to health facilities is greater or malaria transmission is lower, HMM is less effective and potentially more costly than delivery of care through health facilities. The model also suggests that delivery of ACTs through HMM in certain settings may have no advantage over the use of non-ACT regimens, despite their lower overall effectiveness. HMM can be cost-effective, but not in all settings. Decisions to deploy HMM should take into account the local variation in malaria transmission and alternative sources of health care. Strengthening diagnostic capacities both within health facilities and in HMM programs could enhance positive health outcomes in the management of febrile illness.

**C.7 Summary of Preliminary Studies.** This research proposal will greatly benefit from the existence of a sentinel site malaria surveillance system which represents a diversity of epidemiological settings and is strongly support by government programs. Existing strengths include health facility based outpatient surveillance, measuring malaria incidence in cohorts, and experience with economic evaluations as summarized above. Formal collaborations have been established by world-renowned groups already working in Uganda to expand our activities to include inpatient surveillance, population-level surveys, and entomological evaluations. This comprehensive approach to malaria surveillance will allow us to build capacity in Uganda and establish a research agenda designed to evaluate innovative approaches to malaria surveillance and improve our ability to measure the impact of key malaria control interventions.

**D. RESEARCH DESIGN AND METHODS**

**D.1 Overall Design.** We will collect comprehensive malaria surveillance data at multiple sites in Uganda with varying transmission intensity with the overall objective of optimizing surveillance methodology and measuring the impact and cost-effectiveness of control interventions. Comprehensive malaria surveillance will be defined as an approach to surveillance which simultaneously looks at transmission, infection, and disease. The project will initially compare different surveillance methodologies in 3 sentinel districts. In the first year of the project we will enumerate and map all household within the catchment areas of our 3 districts to generate a sampling frame for random sampling of households for surveillance studies. In years 2-3 we will undertake entomological surveys to measure transmission intensity using both CDC light traps and more extended evaluations using human-landing collections, perform repeated cross-sectional surveys at both the level of the household and primary school, and conduct longitudinal studies in cohorts of children to measure malaria incidence. Outpatient data will be collected at level IV government health facilities for measures of malaria morbidity. Inpatient data will be collected at district hospitals for measures of and mortality/severe disease. We will use this data to compare different methodologies for generating estimates of transmission, infection, and disease as outlined in specific aim 1. In years 4-6 we will expand the number of sentinel districts from 3 to 6 and streamline our surveillance activities if appropriate (see section D.9 for discussion of analytical plan for comparing different surveillance methodologies). Throughout the course of the project we will collect data on the cost and coverage levels of key malaria control inventions including IRS, ITNs, and ACTs. This data will be used to measure the impact and compare the cost-effectiveness of malaria control interventions as outlined in specific aims 2 and 3. A schematic of these surveillance activities is outlined in Figure 8 below.

**Figure 8. Summary and timeline of malaria surveillance activities.**

Enumeration of all households and primary schools in selected sub-counties

(3 districts)

Collection of outpatient data

(3 districts)

Cohort studies

100 households

~ 300 children age 0.5-10 years

(3 districts)

Biannual surveys

3 primary schools

300 children

(3 districts)

Collection of inpatient data

(3 districts)

Biannual surveys

200 households

~ 300 children age 6-14 years (3 districts)

Monthly extended entomology surveys (3 districts)

Continuous data collection

CDC light traps,

96 households

(3 districts)

End of year 1

End of year 3

End of year 6

Collection of inpatient data

(6 districts)

Continuous data collection

CDC light traps, 96 households

(6 districts)

Biannual surveys,

3 primary schools

300 children

(6 districts)

Collection of outpatient data

(6 districts)

End of year 7

DATA ANALYSIS AND INTERPRETATION

**D.2 Enumeration and mapping of households.** Target areas will include the sub-counties surrounding UMSP level IV health centers in the following 3 districts: Kabale (low transmission), Mubende (medium transmission) and Apac (high transmission). Each sub-county is estimated to have between 20,000-30,000 residents living in 4,000-6,000 households (see Appendix III for maps). Investigators will first meet with local officials and community representatives to discuss the study and plans for the household enumeration and mapping. Teams of study personnel will systematically cover the target areas to identify and enumerate all households. A household will be defined as any single permanent or semi-permanent structure acting as the primary residence for a person or group of people that cook and eat together. Households will be assigned sequential unique identification numbers and locations mapped using hand-held GPS receivers. Readings will be taken from the door of the household, if possible, or from a point that is most representative of the household. At each household, a reading will be taken every five seconds for 2 minutes, and the average values from these readings will be recorded (Easting, Northing, and Altitude) in UTM units. All primary schools within each sub-county will also be uniquely identified and mapped. Enumerations of households and primary schools will provide the sampling frames for the surveillance studies described below. The complete list of households from each sub-county will be stratified into 3 groups for random selection of households for participation in the following surveillance activities: 1) entomology surveys, 2) community surveys, and 3) cohort studies.

**D.3 Entomology surveys.** As part of our comprehensive surveillance studies we will perform two types of entomological surveys in each of our 3 sentinel districts: 1) Extended entomological surveys including man-landing collections, pyrethrum spay collections, and window trap collections. 2) CDC light trap collections.

**D.3.1 Extended entomological surveys.** Extended entomological surveys will be performed in each of our 3 sentinel sites in order to estimate the EIR and to collect data needed for the modeling exercise described in Research Project 2 such as the Basic Reproduction Number. Each month, catches will be made in and around eight randomly selected houses at each site using man-landing catches indoors and outdoors. Two different houses will be used each night, in each site, for four consecutive nights. At each house two collectors will be stationed indoors and two outdoors 5-10m from the house. Human baits will collect mosquitoes landing on their exposed legs using aspirators and flashlights. Collections will be from 6pm to 7am. Mosquitoes will be collected at hourly intervals through the night and processed the following morning. Concurrent indoor and outdoor hourly light trap catches will be made in nearby houses for comparison with night-biting collection. Ten randomly selected houses will be sprayed with an aerosol of non-residual pyrethroids with a piperonyl butoxide (PBO) synergist each month in each site. Collection will take place between 6am-8am. The number of children and adults who slept in the house the previous night will be determined and the presence of ITNs recorded. White sheets will be spread on the floor and over the furniture in the house. Two collectors, one inside the house and one outside, will spray around the eaves with 0.025% pyrethrum emulsifiable concentrate with 0.1% PBO in kerosene. The collector inside the house will then spray the roof and walls. The house will be closed for 10 minutes after which dead mosquitoes will be collected from the sheets and transferred to the field laboratory on moist filter papers in petri dishes. To collect house-leaving mosquitoes, window exit traps will be set at 6pm and collected between 6-7am the following morning. The mosquitoes from each trap will be put into paper cups separately and transferred to the field laboratory for processing. Parity dissections will be performed on 500 of each species each month at each site.

**D.3.2 CDC light trap collections.** At each of the 3 sentinel sites, 96 houses will be randomly selected for CDC light trap collections. Each night 12 traps will be set for 4 nights/week, such that the same 96 houses can be sampled every 2 weeks during the study. Mosquitoes will be sampled using miniature CDC light traps (Model 512; John W. Hock Company, Gainesville, Florida, USA) positioned 1m above the floor at the foot end of the bed where a person sleeps under an untreated bed net. Traps will be set at 7pm and collected at 7am the following morning. If the trap cannot be set-up in the intended house, the trap will be moved to the nearest similar house. If the occupant does not spend the night in the selected room or the trap is faulty, the data will be excluded from the analysis.

**D.3.3 Processing of mosquito specimens and estimation of entomological indicators.** A large number of specimens will be collected from the different sites and from the different collection methods. In order to process them for the different tests to be performed and the analysis and interpretation of the results we will implement a systematic procedure for labeling and recording the specimens which will include the following information: 1) area where the samples were collected, 2) house number (which will be linked to GIS data), 3) method of collection, 4) date of collection, and 4) serial number of the specimen. When processing the specimens, labels will be written in pencil and placed with the relevant specimens in eppendorf tubes and similar information recorded in a register for easy data entry and cross-checking. A list of entomological indicators that will be measured and the methods used to generate this data are summarized in Table 2 below.

Table 2. Entomological indicators measured by each collection method

| **Indicator** | **Extended surveys** | | | **CDC light trap collection** |
| --- | --- | --- | --- | --- |
|  | **Man-landing collections** | **Pyrethrum spray collection** | **Window trap collection** |  |
| Species* | X | X | X | X |
| Vector density | X |  | X |  |
| Biting rate | X | X |  | X |
| Physiological status | X | X | X | X |
| Parity rate | X | X | X | X |
| Sporozoite infection rate* | X | X |  | X |
| Human blood index* | X | X |  |  |
| Proportion resting indoors |  | X | X |  |
| Proportion exiting after blood meal |  | X | X |  |
| Proportion biting early or late | X |  |  |  |

* Laboratory methods for species identification, sporozoite detection, and identification of blood meal source described in the Laboratory Core

**D.4 Cross-sectional surveys.** Two survey methods will be used to collect cross-sectional data: 1) community surveys, and 2) primary school surveys. Both types of surveys will be done twice a year (once during the rainy season and once during the dry season) for two years (4 surveys total) in each of our three sentinel districts.

**D.4.1 Community surveys.** In each of our three sentinel districts, 200 households will be randomly selected to participate in each community survey from the list of households enumerated within the sub-county of interest. Random selection of households without replacement will be repeated for each survey. Selected households will be approached and project personnel will ask the head of the household or, if he/she is not available, another adult resident (18 years of age or older) for verbal consent to participate in the questionnaire part of the survey. If no adult resident is available, households will be revisited up to four times to complete the survey. The house will be considered vacant if no residents are present at the household after four visits. Information sheets will be used to describe the purpose of the survey. Using a standardized questionnaire, information will be gathered on all household members including demographics, socio-economic status, bednet use, and recent fever treatment practices. In addition, if any women living in the household has given birth, she will be questioned about the vital statistics of her children (date of birth of each child, whether the child is still alive, and the age of death for any child) supplemented by verbal autopsy. Written informed consent will be obtained from parents/guardians and assent from all children age 6-14 years living in the household for the collection of a finger-prick blood sample for testing for malaria parasitemia using a rapid diagnostic test (RDT)(ParaCheck, Span Diagnostics, India), preparation of thick and thin malaria blood smears, filter paper blood samples and to record hemoglobin concentration using a portable, battery-operated hemoglobinometer (Hemocue Ltd, Sheffield, UK). All children with a positive RDT and those identified as anemic (hemoglobin levels < 8 g/dL) will be referred to the nearest health facility for treatment according to national guidelines. Transportation costs will be covered by the project.

**D.4.2 Primary school surveys.** In each of our three sentinel districts, three primary schools in the sub-county of interest will be randomly selected to participate in each primary school survey from a complete listing of schools. Random selection of primary schools without replacement will be repeated for each survey. Prior to the onset of the study, staff from participating schools will be sensitized about the study and plans for recruitment, and a copy of the school registers will be obtained. In each school, 100 children plus 20 reserve children (12 boys and 12 girls in each of classes 2-6) will be randomly selected from the school registers using a computer-generated random number table. Prior to the day of the school survey, group meetings will be held with the parents/guardians of selected school children to review the selection criteria for study participation. Those parents who do not attend the group meetings will be visited at home. During the meetings or household visits, the purpose and procedures of the study will be discussed, an information sheet will be distributed, and written informed consent will be sought from the parents/guardians and assent from the children. Parents/guardians will be instructed to come to the school on the day of the survey.

On the day of the survey, consecutive children from the randomization list will be screened for the following study selection criteria: 1) enrollment at participating school in classes 2-6; 2) age 6-14 years; 3) living in the sub-county where the primary school is located for the last six months; 4) parent/guardian willing to give informed consent; 5) child willing to take part in the study and able to give informed assent. A total of 100 children who fulfill the study selection criteria will be enrolled. A standardized study questionnaire will be administered to all children and their parents/guardians to collect information on demographics, socio-economic status, bednet use, and recent fever treatment practices. Each child will be asked to provide a finger-prick blood sample for testing for malaria parasitemia using a RDT, preparation of thick and thin malaria blood smears, filter paper blood samples and to record hemoglobin concentration using a portable, battery-operated hemoglobinometer (Hemocue Ltd, Sheffield, UK). All children with a positive RDT will be treated on site with the nationally recommended age-specific drug dosing regimen by a qualified nurse. Children identified as severely anemic (hemoglobin levels < 8 g/dL) will be referred to the nearest health facility for treatment according to national guidelines. Transportation costs will be provided and costs of treatment will be covered by the project.

**D.4.3 Laboratory procedures for cross-sectional surveys.** Thick blood smears from all RDT-positive children and a random selection of RDT-negative children will be read by two independent microscopists with a third validation QA reading where discrepancies occur. Thick and thin blood smears will be stained with 2% Giemsa for 30 minutes. Parasite densities will be calculated by counting the number of asexual parasites per 200 leukocytes (or per 500 leukocytes, if the count is <10 asexual parasites/200 leukocytes), assuming a leukocyte count of 8000/µl. A blood smear will be considered negative when the examination of 100 high power fields does not reveal asexual parasites. Thin smears will also be read for identifying parasite species in a random sample of children with positive thick smears.

**D.4.4 Management of data collected from cross-sectional surveys.** Laboratory and epidemiological data will be entered into hand-held PDAs. For quality control, check programs will be written into the database to limit the entry of incorrect data and ensure entry of data into required fields. Data from these devices will be transferred monthly to our data core facility in Kampala and stored on a secure server (see Data Management and Statistical Core for further details).

**D.5 Cohort studies.** Based on our enumeration list of households, we will generate a random list of potential households for recruitment. Using door-to-door recruiting, households will be approached sequentially from our random list and those households with at least one child aged 6 months - 10 years will be identified. Interested parents or guardians and all children from the household between the ages of 6 months -10 years will be brought the study clinic or given an appointment to come to the study clinic for screening and enrollment. At the study clinic, interviews will be conducted in the appropriate language with parents or guardians by study physicians. Eligibility criteria will include (1) age 6 months-10 years, (2) lack of intention to move outside of the sub-county during the 2 year follow-up period, (3) agreement to come to the study clinic for any febrile episode or other illness, (4) agreement to avoid medications administered outside the study, (5) provision of informed consent by parents or guardians. All eligible children from the same household will be recruited. Study enrollment will begin the day informed consent is provided. On the day of enrollment children will undergo a history and physical examination and have blood collected by finger-prick for thick blood smear and filter paper storage. Children who have history of fever in the previous 24 hours or a temperature > 37.5ºC (axillary) will have their thick blood smear read urgently in the study clinic. Children with history of fever in the previous 24 hours or a temperature > 37.5ºC (axillary) and a positive blood smear will be diagnosed with malaria and treated as described in section D.2.5.2.

**D.5.1 Follow-up of cohorts.** Study participants will receive all routine medical care at a study clinic located within the UMSP government run level IV health center. Parents/guardians will be asked to bring their child to the study clinic for all medical care. Routine medical care will be provided free of charge and transport cost to and from the clinic will be reimbursed. At each visit for a new medical problem, subjects who are febrile (axillary temperature > 37.5˚C) or report history of fever in the past 24 hours will have blood obtained by finger-prick for a thick blood smear and collection of blood on filter paper. If the thick blood smear is positive, the patient will be diagnosed with malaria (see section D.5.2). If the thick blood smear is negative or not clinically indicated, the patient will be managed by study physicians following existing treatment algorithms designed to provide standardized guidelines for the treatment of common non-malarial illnesses. The local district hospitals will be available for inpatient care and urgent care outside of study clinic hours. Use of a similar approach to longitudinal follow-up in our cohorts in Kampala and Tororo has resulted in excellent cohort retention, with less than 5% of lost person time per year and minimal use of outside medications which could interfere with our ability to accurately estimate the incidence of malaria.

**D.5.2 Diagnosis and treatment of malaria.** We will utilize a passive case detection system to diagnose episodes of malaria based on study subjects presenting to the clinic with fever and/or signs of complicated malaria. All episodes of malaria will be classified as uncomplicated or complicated based on the following criteria: Uncomplicated malaria (all of the following): 1) fever (> 37.5ºC axillary) or history of fever in the previous 24 hours, 2) positive thick blood smear, and 3) absence of complicated malaria. Complicated malaria (any of the following): 1) evidence of severe malaria and parasitemia according to WHO criteria [82], or 2) danger signs (< 3 convulsions over 24 hour period, inability to sit up or stand, vomiting everything, unable to breastfeed or drink, or lethargy) and parasitemia, or 3) parasite density > 500,000/ul. On the day malaria is diagnosed, patients will undergo a standardized clinical history and physical examination. All patients diagnosed with uncomplicated malaria will be treated with artemether-lumefantrine, the recommended first-line treatment in Uganda (twice a day for 3 days according to weight-based dosing guidelines). Patients with complicated malaria will be treated with quinine (10 mg/kg three times a day x 7 days), either as an inpatient (I.V.) or outpatient (oral) in accordance with local guidelines. On the day malaria is diagnosed, patients will receive paracetamol (10mg/kg) to take every 8 hours until the resolution of fever. For patients with anemia (Hb < 10 gm/dL), we will follow Integrated Management of Childhood Illness (IMCI) guidelines: anemic children will be treated with iron sulfate (100 mg daily for 2 weeks) and mebendazole (only children > 1 year of age; 250 mg age 1-2 years; 500 mg > 2 years age; no more than every 6 months).

**D.5.3 Diagnosis and management of non-malarial illnesses.** Patients who are found to have illnesses other than malaria will receive standard-of-care treatment according to diagnostic and treatment algorithms that have been developed by our study team. During follow-up for non-malarial illnesses, blood smears will be done at the discretion of the study physician if the subjects are febrile or report history of fever. If the blood smear is positive, the patient will be diagnosed with a new episode of malaria and managed per study protocol. If a patient is diagnosed with a non-malarial illness at the same time as malaria or during malaria follow-up, treatment of the non-malarial illness will be at the discretion of the physician, but this will have no impact on the management of malaria.

**D.5.4 Routine evaluations.** Routine evaluations will be done in the study clinic every 3 months. Study subjects not seen in the clinic for any consecutive 3 month period will be visited at home and requested to come to the study clinic as soon as possible. Parents/guardians will be asked about visits to outside health facilities and the use of any medications outside the study protocol. The study protocol will be reinforced with discussion regarding the need to come to the study clinic promptly upon the onset of any illness and to avoid use of outside medications. Study subjects who are febrile (axillary temperature > 37.5˚C) or report history of fever in the past 24 hours at the time of their routine evaluation will have blood obtained by finger-prick for a thick blood smear and collection of blood on filter paper. If the thick blood smear is positive, the patient will be diagnosed and treated for malaria (see section D.5.2).

**D.5.5 Cohort study duration.** We will maintain dynamic cohorts based on limiting observation time to children between the ages of 0.5-10 years. Children will be removed from the cohort when they reach 11 years of age. Additional children from participating households will be added to the cohort when they reach 0.5 years of age. We have chosen this age range as it represents the highest risk group in terms of malaria incidence across our levels of transmission intensity. Additionally, cohort study participants will be prematurely withdrawn from follow-up if they meet any of the following criteria: 1) permanent movement out of the sub-county, 2) inability to be located for > 3 months, 3) withdrawal of informed consent, or 4) inability to comply with the study schedule and procedures

**D.6 Outpatient surveillance.** We will collect prospective data on all patients who presented to the outpatient department at one selected level IV government health facility in each of our 3 districts using the currently active UMSP system. A standardized case record form, which has been developed in collaboration with the Uganda MOH health management information system, will be completed for each patient who presents to the outpatient department (see Appendix IV). Patients will be assigned a unique ID number at registration and have demographic data recorded including age, gender, and location of residence. Patients will then be referred to clinicians who will record basic clinical information. Patients with suspected malaria (i.e. fever) will be referred to the laboratory for diagnostic testing and results will be recorded. Clinician will then record all diagnoses given and treatments prescribed and refer the patient back to the registration desk where one copy of the case record form will be retained. At the end of each day all case recorded forms will be entered into an Epi-Info database. At the end of each month databases will be remotely sent to our core facility in Kampala using cellular technology. At our core facility data will be cleaned and merged into a larger database, which will be web-accessible. Having an individual patient database will allow us to easily generate standardized metrics and stratify data across demographic variables (i.e. age and location).

**D.7 Inpatient surveillance.** We will collect prospective data on all children < 12 years of age who are admitted to the district hospitals in each of our 3 districts using a system analogous to the one described above. A standardized case record form will be used to collect data on clinical examination, residence, laboratory investigations, discharge diagnoses, and outcomes for each child and entered in a central database. Clinical examination will include an assessment of the level of consciousness and children will be given a Blantyre coma score. All children admitted to the hospital will have blood obtained by finger-prick for a thick blood smear and hemoglobin measurement. Hospitalized malaria will be defined as any admission with fever or history of fever and a thick blood smear positive for malaria parasites. Severe malarial anemia will defined as a positive thick smear and a hemoglobin concentration of less than 5 gm/dL. Cerebral malaria will defined as a positive thick smear and a Blantyre coma score of less than 3. These definitions will not be mutually exclusive. Although impaired consciousness might not be due to parasites in some patients, this is the most objective definition of cerebral malaria and can be uniformly applied to all admissions. Malaria-associated death will be defined as death during hospitalization of a patient given a diagnosis of hospitalized malaria.

**D.8 Streamlined surveillance.** In years 4-6 we will expand the number of sentinel districts to 6 by adding the following 3 districts: Kanungu (low transmission), Jinja (medium transmission) and Tororo (high transmission)(see maps of these districts in Appendix III). Pending the comparative evaluation of our different surveillance methodologies (see section 9.1), surveillance activities will be streamlined and limited to entomological surveys using CDC light traps, cross-sectional surveys using primary schools, outpatient surveillance, and inpatient surveillance as described above. We project that the estimates generated from these streamlined surveillance activities will provide accurate outcome measures to be used in specific aim 2 to assess the impact of malaria control interventions. Expanding from 3 to 6 sentinel districts will increase our statistical power and our ability to measure the impact of control interventions across a wider range of epidemiological settings and coverage levels.

**D.9 Analytic and statistical considerations.**

**D.9.1 Specific Aim 1. To identify optimal strategies for malaria surveillance in Uganda by comparing different methodologies in multiple sites with varied transmission intensity.** We will perform comprehensive malaria surveillance studies at 3 sites in Uganda to measure key metrics of malaria transmission (entomological inoculation rates), infection (parasite rates), and disease (measures of malaria morbidity and mortality). Surveillance methodologies using “streamlined” techniques designed to improve efficiency and feasibility will be compared with “gold standard” techniques with the goal of identifying optimal strategies for surveillance across a range of transmission intensities (Table 3).

**Table 3. Measurements for specific aim 1**

| **Category** | **Primary Metric** | **Age range of target population** | **Gold standard**  **method** | **Streamlined**  **method** |
| --- | --- | --- | --- | --- |
| Transmission | EIR | N/A | Human landing collections | CDC light traps |
| Infection | Parasite rate | 6-14 years | Community surveys | Primary school surveys |
| Morbidity | Changes in malaria incidence | 0.5-10 years | Cohort studies | Outpatient surveillance |
| Mortality | Changes in malaria mortality | < 12 years | Community surveys with VA | Inpatient surveillance |

**D.9.1.1 Transmission intensity.** The primary metric used to measure transmission intensity for both the gold standard method and streamlined method will be the EIR, defined as the number of infectious bites per person per unit time, or equivalently, the human biting rate multiplied by the sporozoite rate. For the gold standard method, the human biting rate will be measured directly from the human landing collections done in 8 houses each month. For the streamlined method, the human biting rate will be measured from the number of mosquitoes collected using CDC light traps in 96 houses done twice a month (192 collections per month). The sporozoite rate will be calculated as the number of mosquitoes infected with sporozoites divided by the total number of mosquitoes examined using each respective method of mosquito collection. Each month up to 500 randomly selected mosquitoes will be examined for sporozoites for each method used.

We will test the hypothesis that annual estimates of EIR will be similar using the two methodologies. Point estimates of EIR using both methods will be calculated at monthly intervals and compared annually using the non-parametric Wilcoxon matched-pairs signed-ranks test. Sample sizes will be determined by the human biting rate, number of mosquitoes tested for sporozoites, and the sporozoite rate. Power calculations based on published estimates of the human biting rate and sporozoite rate in high and low transmission intensity settings [4] are presented in Table 4. In our high transmission setting, we will have 80% power (one-sided significance level of 0.05) to reject the alternative hypothesis that there is a difference between our estimates assuming that the annual EIR using the gold standard method is 1586 and the annual EIR using the streamlined methods is within the range of 1205-1964. In our low transmission setting, we will have 80% power (one-sided significance level of 0.05) to reject the alternative hypothesis that there is a difference between our estimates assuming that the annual EIR using the gold standard method is 4 and the annual EIR using the streamlined methods is within the range of 0–24.

Table 4. Power calculations for comparing estimates of EIR

| Level of transmission intensity | Gold standard method estimates | | | Streamlined method |
| --- | --- | --- | --- | --- |
|  | Average nightly human biting rate | Average sporoziote rate | Annual EIR | Range of annual EIRs for which no significant difference detected |
| High | 108 | 0.0402 | 1586 | 1205 - 1964 |
| Low | 7 | 0.00157 | 4 | 0 - 24 |

Whether or not these analyses show any evidence of significant differences between the methods, we will explore whether there is any systematic bias in the estimate of the streamlined method compared to the gold standard method (e.g. the streamlined method consistently over- or underestimates the EIR). If systematic bias is found, ecological log-linear regression models will be used to predict the result of the gold standard method using data from the streamlined method, allowing adjustment for bias. Final models will then be prospectively validated in independent test sets to insure their accuracy.

**D.9.1.2 Infection.** The primary metric used to measure infection for both the gold standard method and streamlined method will be the parasite rate (PR), defined as the number of positive blood smears divided by the number of smears examined. For the gold standard method, PR will be measured using cross-sectional surveys of an estimated 300 children (200 households x 1.5 children per household) aged 6-14 years from our community surveys. For the streamlined method, PR will be estimated using cross-sectional surveys of 300 children (3 schools x 100 children per school) aged 6-14 years from our primary school surveys.

We will test the hypothesis that estimates of PR will be similar using the two methodologies. Point estimates of PR will be calculated for each biannual survey and compared using the McNemar’s test of paired proportions. Assuming that we will have a sample size of 300 children per survey for each methodology, we will have 80% power (one-sided significance level of 0.05) to reject the alternative hypothesis that there is a difference in estimates of PR assuming that the absolute difference in PR is no greater than 10% across a range of PRs in the community surveys of 20-80%. Whether or not these analyses show any evidence of significant differences between the methods, we will explore whether there is any systematic bias in the estimate of the streamlined method compared to the gold standard method (e.g. the streamlined method consistently over- or underestimates PR). Ecological logistic regression models will be used to predict the result of the gold standard method using data from the streamlined method, allowing adjustment for bias. Final models will then be prospectively validated in independent test sets to insure their accuracy.

**D.9.1.3 Morbidity.** It will not be possible to compare point estimates of malaria morbidity using our different methodologies due to differences in the units of measurement. Therefore, the primary metric for malaria morbidity will be the relative change in the incidence of malaria over time (r∆Im) defined as an incidence rate ratio = Im*_i+1_* / Im*_i_ ,* where Im*_i_* represents the incidence of malaria during the baseline time interval and Im*_i+1_* represents the incidence of malaria during interval immediately following using two month time intervals. For example if the Im*_i_* = 2 episodes per person year for Jan-Feb and Im*_i+1_* = 1 episode per person year for Mar-Apr, the r∆Im would equal 0.5 (e.g. 2-fold decrease in the incidence of malaria). For the gold standard method, r∆Im will be measured directly from our cohort studies in children 0.5-10 years of age where malaria incidence will be equal to the number of cases of malaria per person per unit time. For the streamlined method, r∆Im will be estimated using the SPR among children 0.5-10 years of age captured in our outpatient surveillance system by the formula described in section C.3.

We will test the hypothesis that estimates of r∆Im will be similar using the two methodologies. Point estimates of r∆Im from one 6 month time period to the next will be compared by calculating the variance of the streamlined method using the delta method and the variance of the gold standard method using a bootstrap test. Assuming that we will have a sample size of 1800 blood smears per 6 month period for SPR and 300 children in cohorts, and assuming baseline SPR’s of 20%, 40%, and 70%, and baseline malaria incidence rates of 2, 4, and 6 cases per person year for low, medium, and high transmission areas respectively, we will have 80% power (one-sided significance level of 0.05) to reject the alternative hypothesis that there is an absolute difference in estimates of r∆Im of 0.3, 0.23 and 0.22 for low, medium, and high transmission sites, respectively. Whether or not these analyses show any evidence of significant differences between the methods, we will explore whether there is any systematic bias in the estimate of the streamlined method compared to the gold standard method (e.g. the streamlined method consistently over- or underestimates r∆Im). Ecological log-linear regression models will be used to predict the result of the gold standard method using data from the streamlined method, allowing adjustment for bias. Final models will then be prospectively validated in independent test sets to insure their accuracy.

**D.9.1.4 Mortality.** It will not be possible to compare point estimates of malaria mortality using our different methodologies due to differences in the units of measurement. Therefore, the primary metric for malaria mortality will be the relative change in absolute measures of malaria mortality. For the gold standard method, the measure of malaria morality will be the number of malaria-associated deaths in children under 5 years of age per 1,000 live births. Data on malaria mortality using the gold standard method will come from biannual community surveys and verbal autopsy. For the streamlined method, the measure of malaria morality will be the number of malaria-associated deaths in children under 5 years of age captured by our inpatient surveillance system.

We will test the hypothesis that relative changes in malaria mortality will be similar using the two methodologies. Point estimates of relative mortality from one year to the next will be calculated and compared, by calculating the variance of the streamlined method using the delta method and the variance of the gold standard method using a bootstrap test. Assuming that we will have a sample size of 800 children per year from our community surveys with baseline mortality rates of 6.5, 12.9, and 19.4 per 1000 (based on 2006 Uganda DHS survey and assuming malaria-attributable mortality of 20%, 40%, and 60%), and baseline number of malaria-associated deaths from our inpatient surveillance of 12, 60, and 84 (based on preliminary inpatient surveillance data) for low, medium, and high transmission sites respectively, we will have 80% power (one-sided significance level of 0.05) to reject the alternative hypothesis that there is an absolute difference in estimates of relative mortality of 1.30, 0.50 and 0.41 for low, medium, and high transmission sites, respectively. Whether or not these analyses show any evidence of significant differences between the methods, we will explore whether there is any systematic bias in the estimate of the streamlined method compared to the gold standard method (e.g. the streamlined method consistently over- or underestimates relative mortality). Log-linear regression models will be used to predict the result of the gold standard method using data from the streamlined method, allowing adjustment for bias.

**D.9.1.5 Decision on the optimal methods for surveillance.** If any of the streamlined methods described above do not approximate the corresponding gold standard methods and attempts at correcting systematic bias are not reliable (e.g. unable to consistently predict estimates using the gold standard method) then we will conclude that the streamlined method is not a reasonable substitute for the gold standard method. If any of the streamlined methods are rejected then we will continue with the gold standard method when expanding from 3 to 6 sentinel sites for years 4-6 as described in Figure 8.

**D.9.2 Specific Aim 2. To estimate the impact of key malaria control interventions on measures of transmission intensity, infection, and disease using surveillance data at multiple sites in Uganda.** We will prospectively assess the coverage of key malaria control interventions (ITNs, IRS and ACTs) and measure metrics of malaria transmission, infection, and disease at multiple sites in Uganda. Statistical techniques will be used to estimate the impact of changing coverage levels of control interventions on our outcome measures. Over the course of the project we will expand the number of sentinel sites from 3 to 6 to increase the diversity of epidemiological settings and levels of control interventions.

**D.9.2.1 Measurement of coverage levels of key malaria control interventions.** Detailed data will be collected prospectively on coverage levels of key malaria control interventions as detailed in Table 5. Data will be collected from 3 sentinel districts in years 2-3 and then expanded to 6 districts in years 4-6. Data on IRS coverage will come from the Uganda MOH and implementing partners. Data on ITN coverage and ACT use will come from the cross sectional surveys and outpatient surveillance system as described above.

**Table 5. Malaria control intervention variables of interest**

| **Category** | **Metric** | **Source of data** |
| --- | --- | --- |
| IRS | Date, formulation, and proportion of households sprayed | MOH records |
| ITNs | Percentage of household with at least one bednet | Cross-sectional surveys |
|  | Percentage of household with at least one ITN |  |
|  | Average number of nets per household |  |
|  | Average number of ITNs per household |  |
|  | Percentage of children age who slept under any net the prior night |  |
|  | Percentage of children age who slept under an ITN the prior night |  |
| ACTs | Proportion of febrile episodes in children treated with an ACT | Cross-sectional surveys |
|  | Proportion of antimalarial doses prescribed that were ACTs | Outpatient surveillance |
|  | Number of ACT doses prescribed at health care facility per month | Outpatient surveillance |

**D.9.2.2. Measurement of malaria outcome variables of interest.** Detailed data on outcome variables of interest will come from the comprehensive surveillance studies described in sections D.3-D.7 above and summarized in Table 6 below.

**Table 6. Malaria outcome variables of interest**

| **Category** | **Metric** | **Source of data** |
| --- | --- | --- |
| Transmission | EIR | Entomology surveys |
| Infection | Parasite rate | Cross-sectional surveys |
| Morbidity | Slide positivity rate | Outpatient surveillance |
|  | Prevalence of anemia (Hb < 8gm/dL) | Cross-sectional surveys |
| Mortality and  severe disease | Number of deaths due to malaria | Inpatient surveillance |
|  | Number of cases of severe malaria |  |
|  | Age distribution of malaria deaths |  |
|  | Age distribution of severe malaria cases |  |

**D.9.2.3 Statistical modeling to measure the impact of changing coverage levels of malaria control interventions.** We will use observational data on intervention and outcome variables to estimate the causal effect of interventions. These analyses will be performed on a population rather than individual level, as many of the variables of interest (Tables 5 and 6) are only available at a population level (population here defined as a particular sentinel site). To model the population effect of interventions for infectious processes, such as malaria, such population-level analyses are in fact preferable to individual-level analyses, as standard assumptions about independent outcomes between subjects are violated when disease dynamics are nonlinear [83]. Indeed, population-level analyses may be more powerful as well as less biased in such situations [83].

To obtain appropriate causal inference on the effect of interventions on a population, we will use a counterfactual framework to estimate what the outcome variable of interest would be if the malaria control intervention were set to a specific level (e.g. what PR would be if ITN coverage was set to 25%). In this framework, the counterfactual or missing data are defined as the unobserved levels of the intervention for a given population at a certain time. To evaluate the effect of an intervention (i.e. different levels of the counterfactual), we will fit a statistical model to appropriate covariates, then use the model to predict different outcomes at different levels of the intervention for a given population by changing the value of the covariate of interest (e.g. how much would the PR change if ITN coverage increased from 25% to 50%).

The unit of analysis will be data from a single sentinel site during a 6-month period. An interval of 6 months was chosen as all intervention and exposure variables will be measured at least every 6 months. In addition, effects of interventions are expected to lag over the period of a few months or longer, so little information should be lost in aggregating variables collected more frequently than every 6 months. Analyses will be performed across all sentinel sites, allowing us to evaluate the potentially different effects of interventions when performed in areas of differing transmission intensity (Section C.5). However, estimates of the effect of interventions will only be evaluated for transmission intensities where these effects are identifiable (e.g. if no IRS is performed in medium transmission sites, we will not attempt to evaluate the effect of IRS in medium transmission sites even though our model could in theory compute an estimate).

Our dependent variables will be the outcome variables of interest (Table 6) for each site for each 6-month interval. Independent variables will include the outcome of interest from the prior interval (to assess change in the outcome), EIR and parasite rate from the prior time interval (to incorporate transmission intensity), and values of control intervention variables (Table 6) from appropriate prior intervals. Specifically, ITN use (proportion of households with at least one ITN) and ACT use (proportion of febrile episodes in children treated with an ACT) will be incorporated from only the prior interval, as any longer term effect of these interventions should be mediated through transmission intensity which is already parameterized. In contrast, IRS may have a longer term, cumulative effect (Section C.5) and will be parameterized as two covariates: (number of sprayings in the last 10 years) x (proportion of households sprayed); (months since last spraying)^-1^ x (proportion of households sprayed).

Relationships between interventions and outcomes are likely to be non-linear and non-independent. Therefore, the use of standard linear models is unlikely to approximate true relationships, and pre-specified parametric models are likely to result in biased estimates. Numerous data-adaptive prediction algorithms (e.g. generalized boosted regression [84], multivariate adaptive polynomial spline regression [85], or random forests [86]) exist to provide flexible estimates that may more closely reflect complex relationships found in nature, however it is difficult to predict a priori which of these algorithms will perform best with a particular type of data. Choosing the algorithm with the best prediction in a given data set (i.e. smallest residuals) is likely to result in over-fitting and produce a model which is not generalizable to other data sets. A data-adaptive approach that provides flexible estimates while avoiding over-fitting called Super Learner has recently been shown to possess strong theoretical advantages over other methods as well as providing excellent prediction in practice [87]. In this approach, numerous pre-specified prediction algorithms, which may include simple least-squares regression models and more data-adaptive approaches, are fit on the data set. V-fold cross validation is used to select a convex combination of algorithms which minimizes a specified loss function (in this case L2-loss), then uses this combination of predictors fit on the entire data set to produce a final prediction for each data point. Super Learner prediction models will be fit to the outcome of interest using the appropriate model family for the dependent variable (e.g. Poisson for counts, binomial for proportions). The effect of an intervention on a given population will then be assessed by setting the counterfactual to desired levels and using the Super Learner to predict the outcome given the covariates for that population. Confidence intervals for each prediction will be calculated using a bootstrap procedure. Plots of outcomes versus levels of a given intervention will be produced to visualize the predicted effects of interventions for a given population. Specific parameters of interest (e.g. change in parasite prevalence for population “A” if ITN use goes from 25% to 50%) and confidence intervals associated with those parameters can be performed using the estimates and bootstrap-derived inference of the parameters.

The accuracy of predictions using past data will be validated prospectively as interventions are performed. Of note, predictions of outcomes given interventions will also be produced in research project 2 (modeling) using a very different, parametric approach as opposed to the more empirical one outlined here. These analyses will thus be complementary, and can both be prospectively validated to compare accuracy of predictions as well as used to improve the other. For example, parametric transmission models from research project 2 may be incorporated into the Super Learner as one of a number of a priori specified prediction algorithms.

**D.9.3 Specific Aim 3. To conduct an economic evaluation of malaria control interventions to identify the optimal coverage levels and mix of interventions at multiple sites in Uganda.** We will prospectively collect data on the coverage and costs of key malaria control interventions at multiple sites in Uganda with variable transmission intensity. We will then model the cost-effectiveness of different interventions and their combinations at selected coverage levels as measured by various key impact indicators and disability adjusted life-years (DALYs) averted. This will allow us to determine the optimal level of coverage mix of interventions that maximizes health outcomes in different transmission settings.

**D.9.3.1 Model design.** Two types of model structures are commonly used in economic evaluation, decision trees and Markov models [88, 89]. Although the design of the economic evaluation model in this study is expected to evolve during the data collection process, we anticipate developing a Markov model to compare the cost-effectiveness of malaria control interventions alone and in combination from a variety of perspectives. Markov models are useful for evaluating diseases or treatments that recur, including recurrent acute diseases, such as malaria. In constructing a Markov model, the disease is divided into distinct health states which are assigned transition probabilities that dictate the movement of patients between the states over discrete time periods, or cycles. After attaching estimates of cost and health outcome to the states, it is possible to evaluate the costs and outcomes associated with a disease and particular health care interventions by running the model over many cycles [88].

For example, five health states could be defined to represent malaria in children (Figure 9) including 1) State A - Healthy, in which a child is afebrile and is susceptible to illness; 2) State B - Fever/malaria, representing a single episode of symptomatic malaria per cycle; 3) State C - Fever/malaria, representing two or more episodes of symptomatic malaria per cycle; 4) State D - Severe malaria; and 5) State E - Death. The arrows in the diagram in Figure 9 represent possible transitions which may occur between health states from one cycle to the next. The circular arrows alongside States A, B, C, and D indicate that a child may remain in the state s/he was in during the previous cycle. Death is referred to as an ‘absorbing state’, as it is impossible to transition out of this state. For each cycle of the model, it is assumed that each child will enter the model at time 0 in a healthy state and transition between the Markov states at pre-defined probabilities. To run the model, a hypothetical cohort of 1000 individuals would be evaluated over 12 one-month cycles, equivalent to one year of follow-up.

**Figure 9. Markov model structure**

**
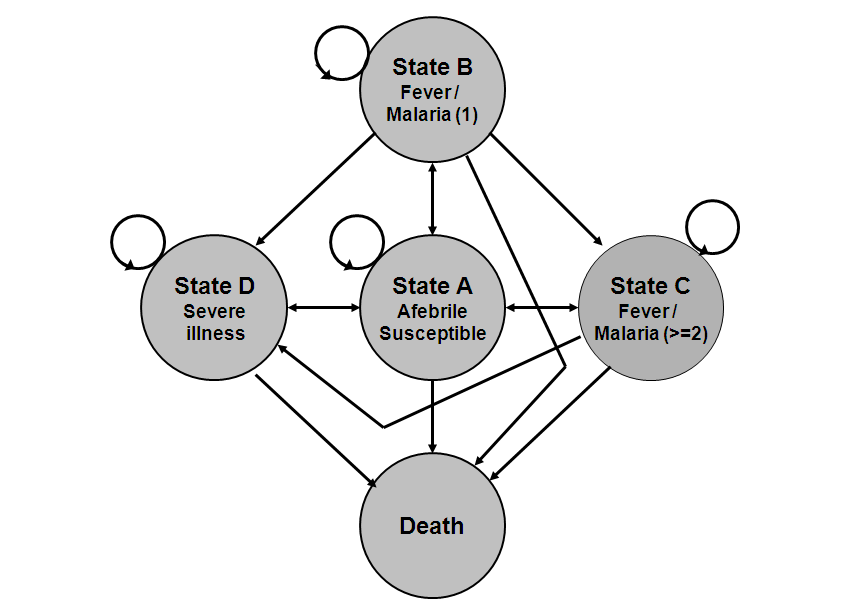
**

**D.9.3.2 Data collection.** The model will include the following parameters: coverage and costs of interventions, transition probabilities, and measures of effectiveness (Table 7). Detailed data will be collected prospectively on coverage levels of key malaria control interventions as detailed in Table 5. Data on the costs of interventions, including the capital and recurrent costs, and household costs will be collected using the ingredients approach. Capital costs will be annuitized where relevant and a discount rate of 3% will be applied. All costs will be converted into US$ based on official yearly average exchange rates for the period during which the costs were incurred and deflated to 2010 prices using the GDP deflator. The model will be designed to compare the interventions from both the provider perspective, which will include capital and recurrent costs, and the societal perspective, which will also include expenditures at the household level. All costs directly related to the research will be excluded. Data on the direct measures of effectiveness, including number of persons and children under five protected by ITNs and IRS, clinical cases averted, and child deaths averted will be collected through the proposed surveillance activities. A DALY will be calculated by considering the years of life lived with a disability and the years of life lost, using standard life tables and disability weights [59]. Weights are typically taken from the Global Burden of Disease study, which assigned specific weights, ranging from 0 (death) to 1 (perfect health), to six different disability classes in attempt to capture the preferences for a given health state [90]. Transition probabilities will be estimated using data collected on children enrolled in the cohort studies.

**Table 7. Model input parameters: costs and effectiveness measures of** malaria control interventions

|  | **ITNs** | **IRS** | **ACTs** |
| --- | --- | --- | --- |
| **Capital costs** | Buildings  Vehicles  ITNs  Other equipment  Start-up costs | Buildings  Vehicles  Sprayers  Other equipment  Start-up costs | Microscope  Start-up costs |
| **Recurrent costs** | Insecticide (if re-treating nets)  Personnel  Fuel/maintenance  Management costs  Training meetings  Office rental  Supplies  Recurrent building costs  Advertising  Basic monitoring and evaluation | Insecticide  Personnel  Fuel/maintenance  Management costs  Training meetings  Office rental  Supplies  Recurrent building costs  Basic monitoring and evaluation | Drugs  Staff salaries  Building rental  Utilities  Consumables  Rapid diagnostic tests  Basic monitoring and evaluation |
| **Household costs** | Bednets/ ITNs  Mosquito prevention costs  Medication  Clinic/hospital fees  Transport costs  Indirect costs | Bednets/ ITNs  Mosquito prevention costs  Medication  Clinic/hospital fees  Transport costs  Indirect costs | Medication  Clinic/hospital fees  Transport costs  Indirect costs |
| **Measures of effectiveness** | Number of persons protected  Number of children under five protected  Clinical case averted  Child death averted  DALYs averted | Number of persons protected  Number of children under five protected  Clinical case averted  Child deaths averted  DALYs averted | Clinical case averted  Child deaths averted  DALYs averted |

**D.9.3.3 Model outputs.** The model will be designed to evaluate the cost-effectiveness of malaria control interventions at various coverage levels, and in different combinations, in different transmission settings. Although the costs and effects of control interventions are not expected to be additive, the model will be designed to assess the impact of different mixes of interventions and to determine the optimal mix in various settings. Comparisons will be made between individual interventions and combinations of interventions, and between interventions at specified coverage levels. The methods for evaluating combinations of interventions will be developed as part of the work package; however, we will likely use regression analysis with the coverage levels of different interventions as covariates to explain differences in malaria related health outcomes**.** The final outcome of the model will include the incremental cost-effectiveness ratio (ICER), and the ceiling ratio (the threshold value that decision makers are willing to pay to avert a DALY). Interventions will be defined as cost-effective if they are less costly and more effective or have an incremental cost per DALY averted of less than a specified decision threshold equivalent to the Ugandan GDP/capita. Alternative scenarios will also be considered, and sensitivity analyses will be conducted, including one-way, multiway, and probabilistic sensitivity analyses to vary key input variables that are either uncertain, or likely to vary in other settings and test the robustness of our results.

**D.10 Timeline and Milestones.**

| **Activity** | **Year 1** | | **Year 2** | | **Year 3** | | **Year 4** | | **Year 5** | | **Year 6** | | **Year 7** | |
| --- | --- | --- | --- | --- | --- | --- | --- | --- | --- | --- | --- | --- | --- | --- |
| ***Research Project 1*** | | | | | | | | | | | | | | |
| Protocol development and approvals |  |  |  |  |  |  |  |  |  |  |  |  |  |  |
| Personnel and training |  |  |  |  |  |  |  |  |  |  |  |  |  |  |
| Household enumeration |  |  |  |  |  |  |  |  |  |  |  |  |  |  |
| Comprehensive surveillance |  |  |  |  |  |  |  |  |  |  |  |  |  |  |
| Streamlined surveillance |  |  |  |  |  |  |  |  |  |  |  |  |  |  |
| Data analysis and measures of impact |  |  |  |  |  |  |  |  |  |  |  |  |  |  |
| Data analysis and measures of cost-effectiveness |  |  |  |  |  |  |  |  |  |  |  |  |  |  |

**Milestones:**

Year 1

- Develop protocols and obtain IRB approvals
- Hire and train staff for the surveillance studies
- Complete the enumeration of households and generate randomization list for sampling

Years 2-3

- Conduct comprehensive surveillance studies in 3 districts
- Performed comparative analysis of different surveillance methodologies to determine whether streamline methods can be used to replace gold standard methods
- Measure the impact and cost-effectiveness of malaria control interventions
- Hold annual meetings with project investigators and policy makers to review data and make recommendations regarding control interventions

Years 4-6

- Begin surveillance studies at 3 additional sites
- Conduct streamlined surveillance studies (if determined to be appropriate) in 6 districts
- Measure the impact and cost-effectiveness of malaria control interventions
- Hold annual meetings with project investigators and policy makers to review data and make recommendations regarding control interventions

Year 7

- Complete the analysis of all surveillance data
- Hold annual meeting with project investigators and policy makers to review data and make recommendations regarding control interventions

**E. LITERATURE CITED**

1. Bhattarai A, Ali AS, Kachur SP, et al. Impact of artemisinin-based combination therapy and insecticide-treated nets on malaria burden in Zanzibar. PLoS Med 2007;4:e309

2. Ceesay SJ, Casals-Pascual C, Erskine J, et al. Changes in malaria indices between 1999 and 2007 in The Gambia: a retrospective analysis. Lancet 2008;372:1545-54

3. O'Meara WP, Bejon P, Mwangi TW, et al. Effect of a fall in malaria transmission on morbidity and mortality in Kilifi, Kenya. Lancet 2008;372:1555-62

4. Okello PE, Van Bortel W, Byaruhanga AM, et al. Variation in malaria transmission intensity in seven sites throughout Uganda. Am J Trop Med Hyg 2006;75:219-25

5. Organization WH. World malaria report 2008, 2008

6. Lynch KI, Beach R, Asamoa K, Adeya G, Nambooze J and Janowsky E. President's Malaria Initiative, Rapid Assessment Report - Uganda, 2005

7. Lalloo DG, Olukoya P and Olliaro P. Malaria in adolescence: burden of disease, consequences, and opportunities for intervention. Lancet Infect Dis 2006;6:780-93

8. Sachs J, Malaney P. The economic and social burden of malaria. Nature 2002;415:680-5

9. Rogier C. Natural history of Plasmodium falciparum malaria and determining factors of the acquisition of antimalaria immunity in two endemic areas, Dielmo and Ndiop (Senegal). Bull Mem Acad R Med Belg 2000;155:218-26

10. White NJ. Intermittent presumptive treatment for malaria. PLoS Med 2005;2:e3

11. Snow RW, Craig M, Deichmann U and Marsh K. Estimating mortality, morbidity and disability due to malaria among Africa's non-pregnant population. Bull World Health Organ 1999;77:624-40

12. Rogier C, Tall A, Diagne N, Fontenille D, Spiegel A and Trape JF. Plasmodium falciparum clinical malaria: lessons from longitudinal studies in Senegal. Parassitologia 1999;41:255-9

13. Clarke SE, Jukes MC, Njagi JK, et al. Effect of intermittent preventive treatment of malaria on health and education in schoolchildren: a cluster-randomised, double-blind, placebo-controlled trial. Lancet 2008;372:127-38

14. Koukounari A, Estambale BB, Njagi JK, et al. Relationships between anaemia and parasitic infections in Kenyan schoolchildren: a Bayesian hierarchical modelling approach. Int J Parasitol 2008;38:1663-71

15. Kurtzhals JA, Addae MM, Akanmori BD, et al. Anaemia caused by asymptomatic Plasmodium falciparum infection in semi-immune African schoolchildren. Trans R Soc Trop Med Hyg 1999;93:623-7

16. Snow RW, Marsh K. The consequences of reducing transmission of Plasmodium falciparum in Africa. Adv Parasitol 2002;52:235-64

17. Talisuna AO, Langi P, Bakyaita N, et al. Intensity of malaria transmission, antimalarial-drug use and resistance in Uganda: what is the relationship between these three factors? Trans R Soc Trop Med Hyg 2002;96:310-7

18. Lengeler C. Insecticide-treated bed nets and curtains for preventing malaria. Cochrane Database Syst Rev 2004:CD000363

19. Zurovac D, Tibenderana JK, Nankabirwa J, et al. Malaria case-management under artemether-lumefantrine treatment policy in Uganda. Malar J 2008;7:181

20. Breman JG, Holloway CN. Malaria surveillance counts. Am J Trop Med Hyg 2007;77:36-47

21. Bryce J, Roungou JB, Nguyen-Dinh P, Naimoli JF and Breman JG. Evaluation of national malaria control programmes in Africa. Bull World Health Organ 1994;72:371-81

22. Habicht JP, Victora CG and Vaughan JP. Evaluation designs for adequacy, plausibility and probability of public health programme performance and impact. Int J Epidemiol 1999;28:10-8

23. Chilundo B, Sundby J and Aanestad M. Analysing the quality of routine malaria data in Mozambique. Malar J 2004;3:3

24. Hay SI, Smith DL and Snow RW. Measuring malaria endemicity from intense to interrupted transmission. Lancet Infect Dis 2008;8:369-78

25. Fontenille D, Simard F. Unravelling complexities in human malaria transmission dynamics in Africa through a comprehensive knowledge of vector populations. Comp Immunol Microbiol Infect Dis 2004;27:357-75

26. Smith DL, Guerra CA, Snow RW and Hay SI. Standardizing estimates of the Plasmodium falciparum parasite rate. Malar J 2007;6:131

27. Brabin BJ. An analysis of malaria in pregnancy in Africa. Bull World Health Organ 1983;61:1005-16

28. Hviid L, Staalsoe T. Malaria immunity in infants: a special case of a general phenomenon? Trends Parasitol 2004;20:66-72

29. Clyde DF. Treatment of multi-drug resistant malaria. East Afr Med J 1967;44:231-7

30. Onori E. Distribution of Plasmodium ovale in the eastern, western and northern regions of Uganda. Bull World Health Organ 1967;37:665-8

31. Draper CC, Smith A. Malaria in the Pare area of Tanganyika. Part II. Effects of three years' spraying of huts with dieldrin. Trans R Soc Trop Med Hyg 1960;54:342-57

32. Smith DL, Dushoff J, Snow RW and Hay SI. The entomological inoculation rate and Plasmodium falciparum infection in African children. Nature 2005;438:492-5

33. Cibulskis RE, Bell D, Christophel EM, et al. Estimating trends in the burden of malaria at country level. Am J Trop Med Hyg 2007;77:133-7

34. Rowe AK, Steketee RW, Arnold F, et al. Viewpoint: evaluating the impact of malaria control efforts on mortality in sub-Saharan Africa. Trop Med Int Health 2007;12:1524-39

35. D'Alessandro U, Olaleye BO, McGuire W, et al. Mortality and morbidity from malaria in Gambian children after introduction of an impregnated bednet programme. Lancet 1995;345:479-83

36. Fegan GW, Noor AM, Akhwale WS, Cousens S and Snow RW. Effect of expanded insecticide-treated bednet coverage on child survival in rural Kenya: a longitudinal study. Lancet 2007;370:1035-9

37. Schellenberg JR, Abdulla S, Nathan R, et al. Effect of large-scale social marketing of insecticide-treated nets on child survival in rural Tanzania. Lancet 2001;357:1241-7

38. Organization wH. Indoor Residual Sprapy. In: Programme GM, ed. Use of indoor residual spraying for scaling up global control and elimination, 2006

39. De Zuleta J, Kafuko GW, McCrae A and al. E. A malaria eradication experiment in the highlands of Kigezi (Uganda). East African Medical Journal 1964;41:102-20

40. Garrett-Jones C. Prognosis for interruption of malaria transmission through assessment of the mosquito's vectorial capacity. Nature 1964;204:1173-74

41. Kouznetsov RI. Malaria control by application of indoor spraying of residual insecticides in Tropical Africa and its impact on population health. Tropical Doctor 1977;7:81-91

42. Barnes KI, Durrheim DN, Little F, et al. Effect of artemether-lumefantrine policy and improved vector control on malaria burden in KwaZulu-Natal, South Africa. PLoS Med 2005;2:e330

43. Guyatt HL, Corlett SK, Robinson TP, Ochola SA and Snow RW. Malaria prevention in highland Kenya: indoor residual house-spraying vs. insecticide-treated bednets. Trop Med Int Health 2002;7:298-303

44. Kleinschmidt I, Sharp B, Benavente LE, et al. Reduction in infection with Plasmodium falciparum one year after the introduction of malaria control interventions on Bioko Island, Equatorial Guinea. Am J Trop Med Hyg 2006;74:972-8

45. Curtis CF, Mnzava AE. Comparison of house spraying and insecticide-treated nets for malaria control. Bull World Health Organ 2000;78:1389-400

46. Kolaczinski K, Kolaczinski J, Kilian A and Meek S. Extension of indoor residual spraying for malaria control into high transmission settings in Africa. Trans R Soc Trop Med Hyg 2007;101:852-3

47. Sinclair D, Zani B, Donegan S, Olliaro P and Garner P. Artemisinin-based combination therapy for treating uncomplicated malaria. Cochrane Database Syst Rev 2009:CD007483

48. Nosten F, van Vugt M, Price R, et al. Effects of artesunate-mefloquine combination on incidence of Plasmodium falciparum malaria and mefloquine resistance in western Thailand: a prospective study. Lancet 2000;356:297-302

49. Bousema JT, Schneider P, Gouagna LC, et al. Moderate effect of artemisinin-based combination therapy on transmission of Plasmodium falciparum. J Infect Dis 2006;193:1151-9

50. Breman JG, Alilio MS and Mills A. Conquering the intolerable burden of malaria: what's new, what's needed: a summary. Am J Trop Med Hyg 2004;71:1-15

51. Creese A, Floyd K, Alban A and Guinness L. Cost-effectiveness of HIV/AIDS interventions in Africa: a systematic review of the evidence. Lancet 2002;359:1635-43

52. Goodman CA, Mills AJ. The evidence base on the cost-effectiveness of malaria control measures in Africa. Health Policy Plan 1999;14:301-12

53. Mulligan JA, Yukich J and Hanson K. Costs and effects of the Tanzanian national voucher scheme for insecticide-treated nets. Malar J 2008;7:32

54. Stevens W, Wiseman V, Ortiz J and Chavasse D. The costs and effects of a nationwide insecticide-treated net programme: the case of Malawi. Malar J 2005;4:22

55. Yukich JO, Lengeler C, Tediosi F, et al. Costs and consequences of large-scale vector control for malaria. Malar J 2008;7:258

56. Conteh L, Sharp BL, Streat E, Barreto A and Konar S. The cost and cost-effectiveness of malaria vector control by residual insecticide house-spraying in southern Mozambique: a rural and urban analysis. Trop Med Int Health 2004;9:125-32

57. Worrall E, Connor SJ and Thomson MC. Improving the cost-effectiveness of IRS with climate informed health surveillance systems. Malar J 2008;7:263

58. Coleman PG, Morel C, Shillcutt S, Goodman C and Mills AJ. A threshold analysis of the cost-effectiveness of artemisinin-based combination therapies in sub-saharan Africa. Am J Trop Med Hyg 2004;71:196-204

59. Goodman CA, Coleman PG and Mills AJ. Changing the first line drug for malaria treatment--cost-effectiveness analysis with highly uncertain inter-temporal trade-offs. Health Econ 2001;10:731-49

60. Morel CM, Lauer JA and Evans DB. Cost effectiveness analysis of strategies to combat malaria in developing countries. Bmj 2005;331:1299

61. Njau JD, Goodman CA, Kachur SP, et al. The costs of introducing artemisinin-based combination therapy: evidence from district-wide implementation in rural Tanzania. Malar J 2008;7:4

62. Wilkins JJ, Folb PI, Valentine N and Barnes KI. An economic comparison of chloroquine and sulfadoxine-pyrimethamine as first-line treatment for malaria in South Africa: development of a model for estimating recurrent direct costs. Trans R Soc Trop Med Hyg 2002;96:85-90

63. Wiseman V, Kim M, Mutabingwa TK and Whitty CJ. Cost-effectiveness study of three antimalarial drug combinations in Tanzania. PLoS Med 2006;3:e373

64. Tediosi F, Maire N, Smith T, et al. An approach to model the costs and effects of case management of Plasmodium falciparum malaria in sub-saharan Africa. Am J Trop Med Hyg 2006;75:90-103

65. Lubell Y, Hopkins H, Whitty CJ, Staedke SG and Mills A. An interactive model for the assessment of the economic costs and benefits of different rapid diagnostic tests for malaria. Malar J 2008;7:21

66. Lubell Y, Reyburn H, Mbakilwa H, et al. The cost-effectiveness of parasitologic diagnosis for malaria-suspected patients in an era of combination therapy. Am J Trop Med Hyg 2007;77:128-32

67. Lubell Y, Reyburn H, Mbakilwa H, et al. The impact of response to the results of diagnostic tests for malaria: cost-benefit analysis. Bmj 2008;336:202-5

68. Shillcutt S, Morel C, Goodman C, et al. Cost-effectiveness of malaria diagnostic methods in sub-Saharan Africa in an era of combination therapy. Bull World Health Organ 2008;86:101-10

69. Willcox ML, Sanogo F, Graz B, et al. Rapid diagnostic tests for the home-based management of malaria, in a high-transmission area. Ann Trop Med Parasitol 2009;103:3-16

70. Zurovac D, Larson BA, Akhwale W and Snow RW. The financial and clinical implications of adult malaria diagnosis using microscopy in Kenya. Trop Med Int Health 2006;11:1185-94

71. Zurovac D, Larson BA, Skarbinski J, Slutsker L, Snow RW and Hamel MJ. Modeling the financial and clinical implications of malaria rapid diagnostic tests in the case-management of older children and adults in Kenya. Am J Trop Med Hyg 2008;78:884-91

72. Goodman CA, Coleman PG and Mills AJ. The cost-effectiveness of antenatal malaria prevention in sub-Saharan Africa. Am J Trop Med Hyg 2001;64:45-56

73. Hutton G, Schellenberg D, Tediosi F, et al. Cost-effectiveness of malaria intermittent preventive treatment in infants (IPTi) in Mozambique and the United Republic of Tanzania. Bull World Health Organ 2009;87:123-9

74. Mbonye AK, Hansen KS, Bygbjerg IC and Magnussen P. Intermittent preventive treatment of malaria in pregnancy: the incremental cost-effectiveness of a new delivery system in Uganda. Trans R Soc Trop Med Hyg 2008;102:685-93

75. Temperley M, Mueller DH, Njagi JK, et al. Costs and cost-effectiveness of delivering intermittent preventive treatment through schools in western Kenya. Malar J 2008;7:196

76. Ssekabira U, Bukirwa H, Hopkins H, et al. Improved malaria case management after integrated team-based training of health care workers in Uganda. Am J Trop Med Hyg 2008;79:826-33

77. Clark TD, Greenhouse B, Njama-Meya D, et al. Factors determining the heterogeneity of malaria incidence in children in Kampala, Uganda. J Infect Dis 2008;198:393-400

78. Bukirwa H, Yau V, Kigozi R, et al. Assessing the impact of indoor residual spraying on malaria morbidity using a sentinel site surveillance system in Western Uganda. Am J Trop Med Hyg in press

79. Hopkins H, Bebell L, Kambale W, Dokomajilar C, Rosenthal PJ and Dorsey G. Rapid diagnostic tests for malaria at sites of varying transmission intensity in Uganda. J Infect Dis 2008;197:510-8

80. Hamer DH, Ndhlovu M, Zurovac D, et al. Improved diagnostic testing and malaria treatment practices in Zambia. Jama 2007;297:2227-31

81. Reyburn H, Mbakilwa H, Mwangi R, et al. Rapid diagnostic tests compared with malaria microscopy for guiding outpatient treatment of febrile illness in Tanzania: randomised trial. Bmj 2007;334:403

82. Organization WH. WHO guidelines for the treatment of malaria, 2006

83. Koopman JS, Longini IM, Jr. The ecological effects of individual exposures and nonlinear disease dynamics in populations. Am J Public Health 1994;84:836-42

84. Friedman JH. Greedy function approximation: A gradient boosting machine. Annals of Statistics 2001;29:1189-1232

85. Friedman JH. Multivariate Adaptive Regression Splines. Annals of Statistics 1991;19:1-67

86. Breiman L. Random forests. Machine Learning 2001;45:5-32

87. van der Laan MJ, Polley EC and Hubbard AE. Super learner. Stat Appl Genet Mol Biol 2007;6:Article25

88. Briggs A, Sculpher M. An introduction to Markov modelling for economic evaluation. Pharmacoeconomics 1998;13:397-409

89. Drummond MF, Richardson WS, O'Brien BJ, Levine M and Heyland D. Users' guides to the medical literature. XIII. How to use an article on economic analysis of clinical practice. A. Are the results of the study valid? Evidence-Based Medicine Working Group. Jama 1997;277:1552-7

90. Murray CJ, Lopez AD. Evidence-based health policy--lessons from the Global Burden of Disease Study. Science 1996;274:740-3

**F. PROTECTION OF HUMAN SUBJECTS**

**F.1 Risks to the Subjects**

**F.1.1 Human subjects involvement and characteristics.** All of the research involving human subjects described in this proposal will involve surveillance studies conducted in Uganda. Surveillance studies involving human subjects will include the following categories:

1) Outpatient and Inpatient surveillance at government health facilities - These activities will only involve the collection of routine surveillance data that is part on the Ugandan Health Management Information System and therefore is exempt from human subjects research. Therefore this category of surveillance studies will not be discussed further.

2) Community surveys – In each of three sentinel districts, 200 households will be randomly selected to participate in community surveys which will be performed twice a year for two years (2400 total household will be surveyed). Selected households will be approached and project personnel will ask the head of the household or, if not available, another adult resident (18 years of age or older) for verbal consent to participate in the questionnaire part of the survey. Questionnaire data will be collected on all household residents (4 residents estimated per household = data collected on a total of 9600 household residents). Written informed consent will be obtained from parents/guardians and assent from all children age 6-14 years living in the household for the collection of a finger-prick blood sample (1.5 children age 6-14 years estimated per household = finger-prick blood samples collected on a total of 3600 children).

3) Primary school surveys - In each of 6 sentinel districts, three primary schools will be randomly selected to participate in cross-sectional surveys. In each school, 100 children (equal numbers of boys and girls) will be enrolled in each survey if they meet the following inclusion/exclusion criteria: 1) enrollment at participating school in classes 2-6; 2) age 6-14 years; 3) living in the sub-county where the primary school is located for the last six months; 4) parent/guardian willing to give informed consent; 5) child willing to take part in the study and able to give informed assent. Primary school surveys will be conducted on a total of 14,400 children (300 children per district per survey x 2 surveys per year x 5 years x 3 districts; 300 children per district per survey x 2 surveys per year x 3 years x 3 districts).

3) Cohort studies - In each of 3 sentinel districts, 100 households with at least one child aged 6 months to 10 years will be identified. All eligible children from the same household will be recruited into the cohort studies if they meet the following inclusion/exclusion criteria: 1) age 6 months-10 years, 2) lack of intention to move outside of the sub-county during the follow-up period, 3) agreement to come to the study clinic for any febrile episode or other illness, 4) agreement to avoid medications administered outside the study, 5) provision of informed consent by parents or guardians. It is estimated that a total of 900 children will be enrolled in the cohort studies (3 children per household x 100 households x 3 districts).

**F.1.2 Sources of materials.**

1) Community surveys - Questionnaire date will be gathered on all household members including information on demographics, socio-economic status, bednet use, and recent fever treatment practices. In addition, if any women living in the household have given birth, they will be questioned about the vital statistics of their children (date of birth of each child, whether the child is still alive, and the age of death for any children) supplemented by verbal autopsy. All children age 6-14 years living in the household will have blood collected by finger-prick for testing for malaria parasitemia using a rapid diagnostic test, preparation of thick and thin malaria blood smears, filter paper blood samples and to record hemoglobin concentration.

2) Primary school surveys – Questionnaire data will be collected from all children and their parents/guardians including information on demographics, socio-economic status, bednet use, and recent fever treatment practices. Each child will be asked to provide a finger-prick blood sample for testing for malaria parasitemia using a rapid diagnostic test, preparation of thick and thin malaria blood smears, filter paper blood samples and to record hemoglobin concentration.

3) Cohort studies - On the day of enrollment children will undergo a history and physical examination using a standardized case record form and have blood collected by finger-prick for thick blood smear and filter paper storage. Study participants will receive all routine medical care at a study clinic. At each visit for a new medical problem a standardized case record form will be filled out and subjects who are febrile (tympanic temperature > 38.0˚C) or report history of fever in the past 24 hours will have blood obtained by finger-prick for a thick blood smear and collection of blood on filter paper.

All records will be kept as confidential as possible. Study participants will be identified only by a unique identification number. Patient names will not be entered into the computerized database. Cross-sectional survey data will be entered directly into handheld PDAs. Cohort study participant case record forms will be kept in individual files in a secured filing cabinet in the study clinics. Additional records will be kept in laboratory record books, which will be stored in the central study laboratory. No individual identities will be used in any reports or publications resulting from the study. Much of the data to be collected for this study will come from tests and procedures normally done in the care of infants and children.

**F.1.3 Potential risks.**

**Loss of Privacy.** Participation in research may involve a loss of privacy for research subjects. Personal and medical information will be collected from subjects and study staff will have access to such data.

**Drawing blood by fingerstick.** This may cause momentary discomfort or soreness when blood is drawn and bruising. Rarely, blood collection may cause local infection.

**F.2 Adequacy of Protection against Risks**

**F.2.1 Recruitment and informed consent.**

1) Community surveys - Selected households will be approached and project personnel will ask the head of the household or, if not available, another adult resident (18 years of age or older) for verbal consent to participate in the questionnaire part of the survey. Written informed consent will be obtained from parents/guardians and assent from all children age 6-14 years living in the household for the collection of a finger-prick blood sample

2) Primary school surveys – Prior to the onset of the study, staff from participating schools will be sensitized about the study and plans for recruitment, and a copy of the school registers will be obtained. Children will be randomly selected from the school registers using a computer-generated random number table. Prior to the day of the school survey, group meetings will be held with the parents/guardians of selected school children to review the selection criteria for study participation. Those parents who do not attend the group meetings will be visited at home. During the meetings or household visits, the purpose and procedures of the study will be discussed, an information sheet will be distributed, and written informed consent will be sought from the parents/guardians and assent from the children.

3) Cohort studies - Using door-to-door recruiting, households will be approached sequentially from a randomization list and those households with at least one child aged 6 months to 10 years will be identified. Interested parents or guardians and all children from the household between the ages of 0.5-10 years will be brought the study clinic or given an appointment to come to the study clinic for screening and enrollment. At the study clinic interviews will be conducted in the appropriate language with parents or guardians by study physicians. Written informed consent by parents or guardians and assent from children > 6 years of age will be obtained for all children who meet the eligibility criteria.

**F.2.2 Protection against risk.**

**Confidentiality.** Successful implementation of the study will require that the confidentiality of all study participants be strictly maintained. For all data collected as part of the studies, participants will be assigned a unique identification number. All data and information collected will be kept in password protected PDAs or locked file cabinets. All project staff will be trained on procedures for maintaining confidentiality and asked to sign a pledge of confidentiality.

**Collection of finger-prick blood samples.** Procedures will be put in place to minimize the risk of infection due to finger-prick blood collection through the use of individually packaged sterile alcohol swabs and gauze.

**F.3 Potential Benefits of the Proposed Research to the Subjects and Others.**

1) Community surveys and primary school surveys – Children who participate in these surveys may benefit from the identification and treatment of malaria parasitemia as part of the active screening process using rapid diagnostic tests.

2) Cohort studies – Study participants will likely benefit from the provision of all routine medical care at no cost. This is a benefit that has been greatly appreciated in our previous cohort studies, as the medical care provided in these studies is generally far superior to that routinely available and costs of medical care are a major difficulty for families in Uganda, where even routine care at public institutions often requires payment. In addition, subjects will be reimbursed for the costs of transport for all visits to our study clinics.

The societal benefit of these surveillance studies is potentially huge, as it is anticipated that the knowledge gained will improve the impact of malaria control interventions.

**F.4 Importance of the Knowledge to be Gained.**

Uganda is a country with an extremely high burden of malaria, but also a wide range of transmission intensities and diverse epidemiological settings. Recent increases in funding from international donors have provided Uganda with the opportunity to greatly expand coverage of malaria control interventions. However, there is currently limited capacity to conduct the high quality research needed to maximize the impact of interventions through an evidence-based approach. The objectives of this project are to establish and validate simplified, reliable, and cost-effective surveillance tools at various sites across Uganda. This work aims to inform the evaluation of malaria control efforts and to provide a model for malaria surveillance in other malaria endemic countries.

**G. INCLUSION OF WOMEN AND MINORITIES.** We will recruit both genders into our study. We do not plan specific efforts to encourage or discourage the enrollment of either sex. All subjects in this study will likely be of African heritage (Ugandan) and we do not plan specific efforts to encourage or discourage the enrollment of persons of any heritage.

**H. TARGETED/PLANNED ENROLLMENT TABLE.** See attached sheet on following page.

**I. INCLUSION OF CHILDREN.** Several aspects of this research proposal will include children. In highly endemic areas such as sub-Saharan Africa, malaria is primarily a disease of children. With advancing age and multiple episodes of malaria, partial immunity develops, such that malarial incidence decreases. The decreased incidence of malaria in older children and adults and the increased antimalarial immunity of these individuals makes them less useful subjects for study, as malaria episodes are less common. In addition, malaria control efforts in Africa are appropriately focused on young children. Specifically our cohorts will enroll children 6 months to 10 years of age, who will be followed for 2 year. Our cross-sectional surveys will focus on children 6-14 years of age. Inpatient surveillance will only be done for children 12 years of age or younger admitted to our sentinel hospitals.

**J. VERTEBRATE ANIMALS.** Not applicable.

**K. SELECTED AGENT RESEARCH.** Not applicable.

**L. MULTIPLE PD/PI LEADERSHIP PLAN.** Not applicable.

#### M. CONSORTIUM/CONTRACTURAL ARRANGEMENTS

A memorandum of understanding is in place between UCSF and Makerere University for scientific collaboration. Dr. Moses Kamya is the director of the Infectious Disease Research Collaboration (IDRC) which has a memorandum of understanding in place with Makerere University for this organization to operate as the legal management center for our Makerere University research and training projects. The IDRC is a registered NGO and is recognized as such by the Republic of Uganda. It is located on the Mulago Hospital campus in Kampala. The IDRC will be responsible for personnel management; purchasing equipment and supplies in Uganda; purchasing, insuring, and maintaining research vehicles at local cost and adhering to UCSF and NIH guidelines; creating and upholding agreements for space for research activities in Uganda; and maintaining and ensuring financial transparency of grants through multiple funding sources including the NIH. IDRC is registered with the Office of Human Research Protections and has been issued a Federal Wide Assurance number (FWA00013482) and DUNS Number (850452797). Funds for collaborators at the LSHTM and KEMRI will be transferred by sub-contract between UCSF and these institutions.

**N. LETTERS OF SUPPORT.** None.

**O. RESOURCE SHARING PLAN.** Not applicable.
